# Supplementary material for: Nucleus accumbens D1- and D2-expressing neurons control the balance between feeding and activity-mediated energy expenditure
Source: Nat Commun. 2024 Mar 21;15:2543. doi: 10.1038/s41467-024-46874-9 (PMC10958053; doi:10.1038/s41467-024-46874-9)

Supplementary Information for

Nucleus accumbens D1- and D2-expressing neurons control the balance between feeding and activity-mediated energy expenditure

Roman Walle [1,*], Anna Petitbon [1], Giulia R. Fois [2], Christophe Varin[3], Enrica Montalban [1], Lola Hardt [1], Andrea Contini [1], Maria Florencia Angelo [1], Mylène Potier [1,4], Rodrigue Ortole [1], Asma Oummadi [1], Véronique De Smedt-Peyrusse [1], Roger A Adan [5], Bruno Giros [6,7], Francis Chaouloff [8], Guillaume Ferreira [1], Alban de Kerchove d'Exaerde[3], Fabien Ducrocq [1], François Georges [2] and Pierre Trifilieff [1,*]

[1] Université de Bordeaux, INRAE, Bordeaux INP, NutriNeuro, 33000, Bordeaux, France

[2] Univ. Bordeaux, CNRS, IMN, UMR5293 F-33000 Bordeaux

[3] Laboratory of Neurophysiology, ULB Neuroscience Institute, WELBIO, Université Libre de Bruxelles (ULB), Brussels, Belgium

[4] Bordeaux Sciences Agro, F-33175 Gradignan, France

[5] Department of Translational Neuroscience, UMC Utrecht Brain Center, University Medical Center Utrecht, Heidelberglaan 100, 3584CG Utrechtthe Netherlands; Altrecht Eating Disorders Rintveld, Zeist, the Netherlands.

[6] Department of Psychiatry, Douglas Hospital, McGill University; Montreal, QC, Canada.

[7] Université de Paris Cité, INCC UMR 8002, CNRS ; F-75006 Paris, France.

[8] Endocannabinoids and NeuroAdaptation, NeuroCentre INSERM U1215, 33077 Bordeaux, France; Université de Bordeaux, 33077 Bordeaux, France

*Correspondence to Pierre Trifilieff (pierre.trifilieff@inrae.fr) or Roman Walle (roman.walle@live.fr)

Contact information: Pierre Trifilieff (Lead contact) or Roman Walle

INRAE, UMR 1286, Laboratoire NutriNeuro, Université de Bordeaux, Batiment UFR Pharmacie - 2eme Tranche - 2eme Etage – CC34, 146 rue Léo Saignat, 33076 BORDEAUX Cedex - France.

pierre.trifilieff@inrae.fr; roman.walle@live.fr

Phone: +33557571248

Supplementary Figures

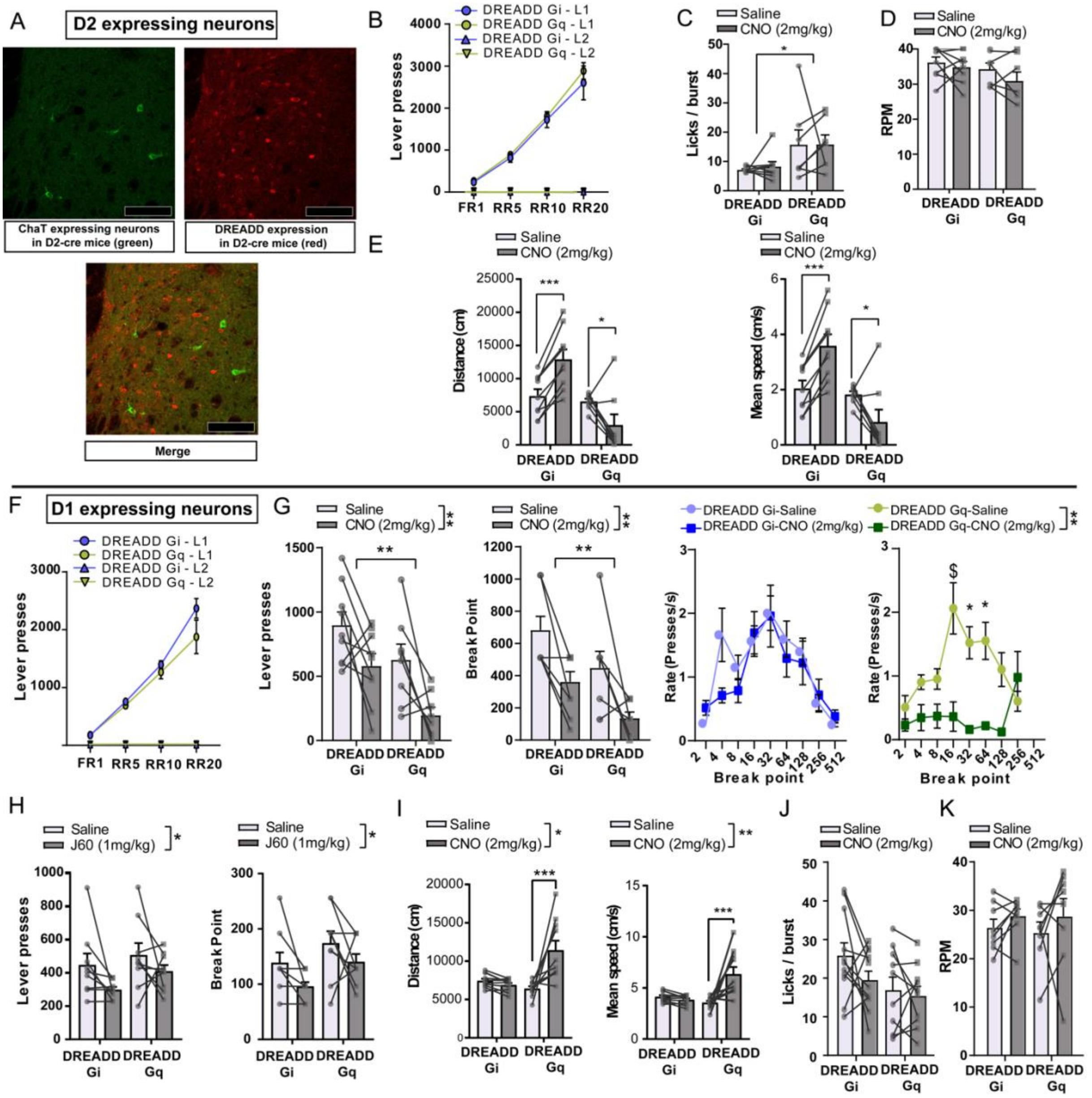

Suppl. Figure 1 (relates to Figure 1):

(A) Representative picture of NAc coronal section with immuno-labeled ChAT (Choline acetyl transferase) in green, (Top left), immuno-labeled DREADD-mCherry in red (Top right) and merge (Bottom). (B) Number of lever presses on the reinforced (L1) and non-reinforced (L2) levers across the different schedules of reinforcement in D2-cre mice expressing either DREADD Gi (n=9) or DREADD Gq (n=7) in the NAc, in the absence of CNO administration. (C) Licking microstructures (number of licks per burst) during consumption of a palatable solution (milk) in D2-cre mice expressing DREADD Gi (n=8) or Gq (n=7). (D) Rotation per min (RPM) in a rotarod in D2-cre mice under inhibition (DREADD Gi, n=8) or activation (DREADD

Gq, n=7) of D2-neurons. (E) Distance (left) and mean speed (right) in an open field in D2-cre mice expressing DREADD Gi (n=9) or Gq (n=8). (F) Number of lever presses on the reinforced (L1) and non-reinforced (L2) levers across the different schedules of reinforcement in D1-cre mice expressing either DREADD Gi (n=9) or DREADD Gq (n=8) in the NAc, in the absence of CNO administration. (G) Effect of 2 mg/kg CNO in D1-cre mice expressing DREADDs Gi (n=9) or Gq (n=8) on the number of lever presses (left), breakpoint (middle) and ratio requirement (right) in the PR task. (H) Effect of the DREADD ligand JHU37160 (J60) in D1-cre mice expressing DREADDs Gi (n=9) or Gq (n=9) on the number of lever presses (left) and breakpoint (right) in the PR task. (I) Distance (left) and mean speed (right) in an open field in D1-cre mice expressing DREADD Gi (n=11) or Gq (n=10). (J) Licking microstructures (number of licks per burst) during consumption of a palatable solution (milk) under inhibition (DREADD Gi, n=11) or activation (DREADD Gq, n=10) of D1-neurons. (K) Rotation per min (RPM) in a rotarod in D1-cre mice under inhibition (DREADD Gi, n=8) or activation (DREADD Gq, n=8) of D2-neurons. $: $0.05<p<0.1$; * : $p<0.05$; ** : $p<0.01$; *** : $p<0.001$; Error bars = s.e.m. Detailed statistics are displayed in Supplementary Table 2. Source data are provided as a Source Data file.

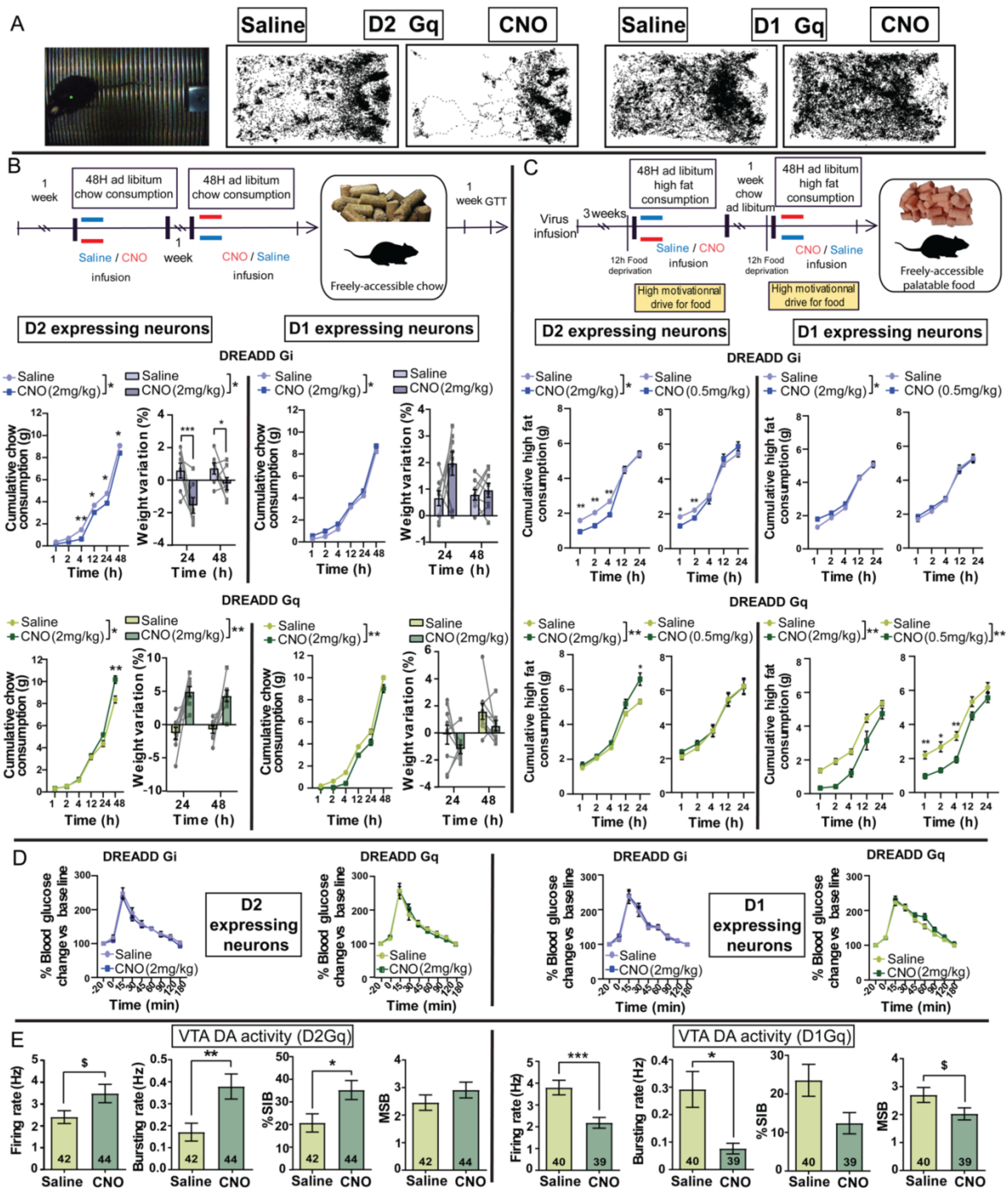

Suppl. Figure 2 (relates to Figure 2):

(A) Representative image for the tracking of animal trajectories (left) and examples of trajectories under activation of D2- and D1-neurons comparing saline and CNO conditions from the same animal during pavlovian conditioning. (B) Experimental design (top), and graphs plotting cumulative chow consumption over 48 hours (left column) and weight variations (right column) under inhibtion (DREADD Gi, n=7 for D2-neurons, n=9 for D1-neurons) or activation (DREADD Gq, n=8 for D2-neurons, n=9 for D1-neurons) of D2- or D1-neurons. (C) Fasting-

refeeding procedure with high fat diet (top) and graphs plotting cumulative food consumption over 24 hours under inhibition (DREADD Gi, n=7 for D2-neurons, n=9 for D1-neurons) or activation (DREADD Gq, n=7 for D2-neurons, n=9 for D1-neurons) of D2- or D1-neurons, using either 2 mg/kg or 0.5 mg/kg of CNO. (D) Glucose tolerance test under acute chemogenetic inhibition (DREADD Gi, n=7 for D2-neurons, n=9 for D1-neurons) or activation (DREADD Gq, n=8 for D2-neurons, n=9 for D1-neurons) of NAc D2-neurons (left) and D1-neurons (right). (E) Firing rate, bursting rate, % spike in burst (SIB) and mean spike in burst (MIB) were assessed through in vivo recording of dopaminergic neurons of the VTA under chemogenetic activation of either D2-neurons (n=6 animals) or D1-neurons (n=4 animals) in saline or CNO conditions under anesthesia. Numbers in bars indicate the number of neurons recorded. $: $0.05<p<0.1$; * : $p<0.05$; ** : $p<0.01$; *** : $p<0.001$; Error bars = s.e.m. Detailed statistics are displayed in Supplementary Table 2. Source data are provided as a Source Data file.

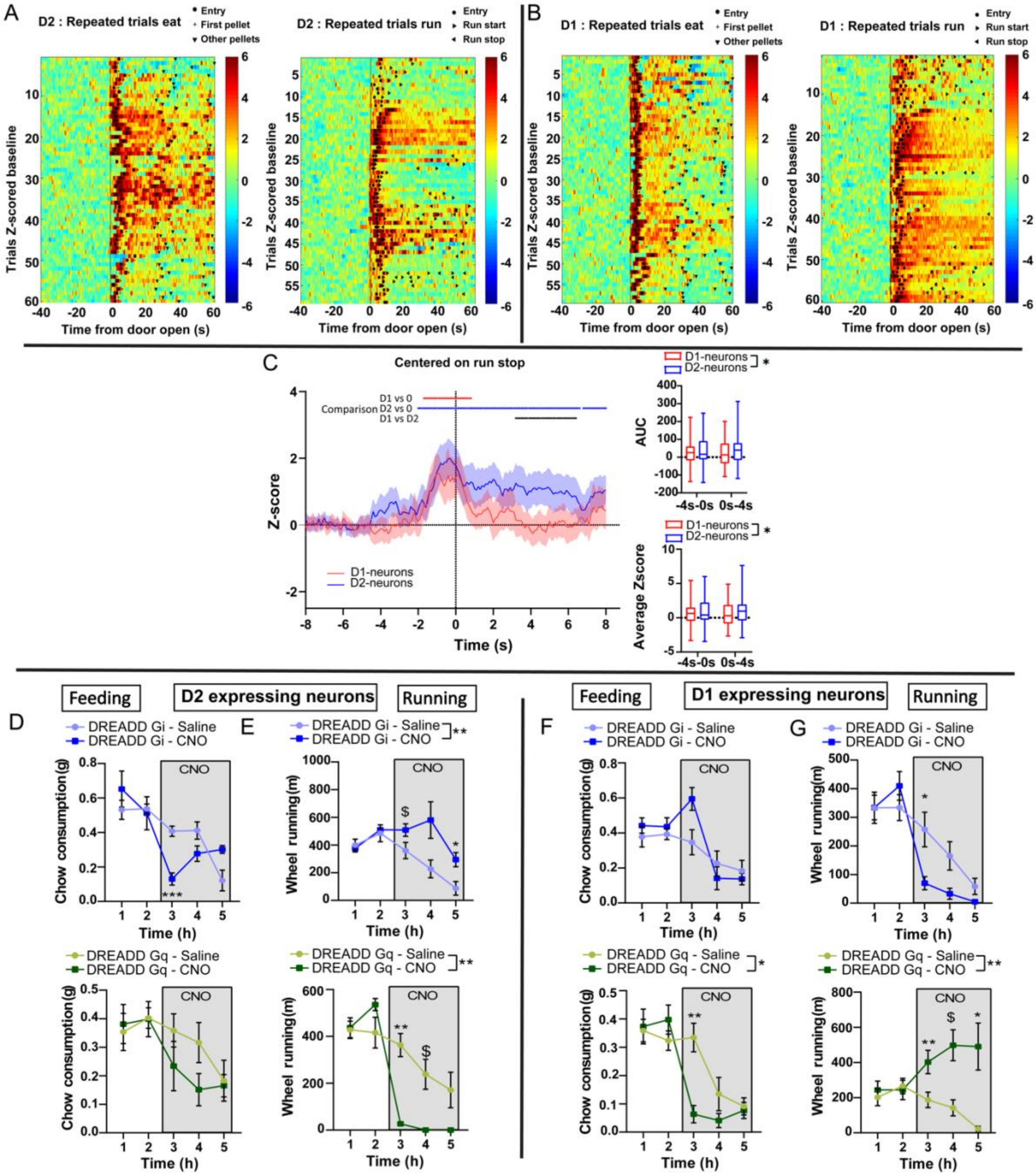

Suppl. Figure 3 (relates to Figure 3):

(A) Heat maps of changes in calcium signal (Z score) in D2-neurons (n=5 animals, 12 trials per animal), for feeding (left) or wheel running (right) trials. Events are aligned to door opening. (B) Heat maps of changes in calcium signal (Z score) in D1-neurons (n=5 animals, 12 trials per animal), for feeding (left) or wheel running (right) trials. Events are aligned to door opening. (C) Peri-event analysis of voluntary run stop and analysis of AUC and average z-score. (D-E) Chow consumption (D) and running (E) under chemogenetic inhibition (DREADD Gi, n=7) or activation (DREADD Gq, n=8) of D2-neurons. (F-G) Chow consumption (D) and running (E) under chemogenetic inhibition (DREADD Gi, n=9) or activation (DREADD Gq, n=9) of D1-

neurons. * : $p<0.05$; ** : $p<0.01$; Error bars = s.e.m. Detailed statistics are displayed in Supplementary Table 2. Source data are provided as a Source Data file.

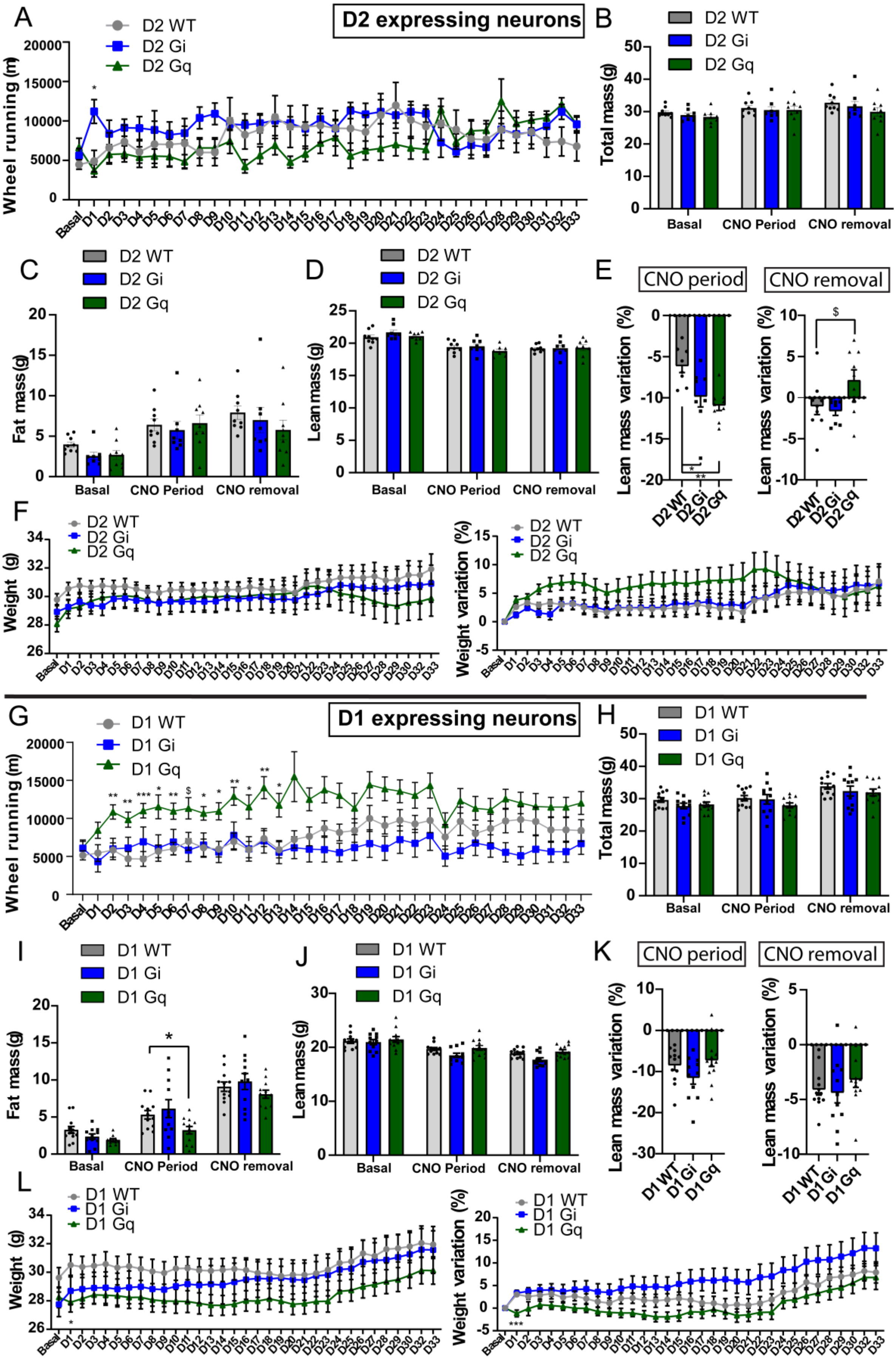

A
D2 expressing neurons
D2 WT
D2 Gi
D2 Gq
Wheel running (m)
B
Total mass (g)
Basal
CNO Period
CNO removal
C
Fat mass(g)
D
Lean mass (g)
E
CNO period
CNO removal
Lean mass variation (%)
F
Weight (g)
Weight variation (%)
G
D1 expressing neurons
D1 WT
D1 Gi
D1 Gq
Wheel running (m)
H
Total mass (g)
I
Fat mass(g)
J
Lean mass (g)
K
CNO period
CNO removal
Lean mass variation (%)
L
Weight (g)
Weight variation (%)

Suppl. Figure 4 (relates to Figure 4):

(A-F) Distance ran (A), total mass at the end of the CNO period (B), fat mass at the end of the CNO period (C), lean mass at the end of the CNO period (D), lean mass variations at the end of the CNO period and after CNO removal (E), weight and weight variation (F) for chemogenetic manipulations of D2-neurons (WT n=7, D2 Gi n=7, D2 Gq n=8). (G-L) Distance ran (G), total mass at the end of the CNO period (H), fat mass at the end of the CNO period (I), lean mass at the end of the CNO period (J), lean mass variations at the end of the CNO period and after CNO removal (K), weight and weight variation (L) for chemogenetic manipulations of D1-neurons (WT n=9, D1 Gi n=11, D1 Gq n=11). $: $0.05<p<0.1$; * : $p<0.05$; ** : $p<0.01$; Error bars = s.e.m. Detailed statistics are displayed in Supplementary Table 2. Source data are provided as a Source Data file.

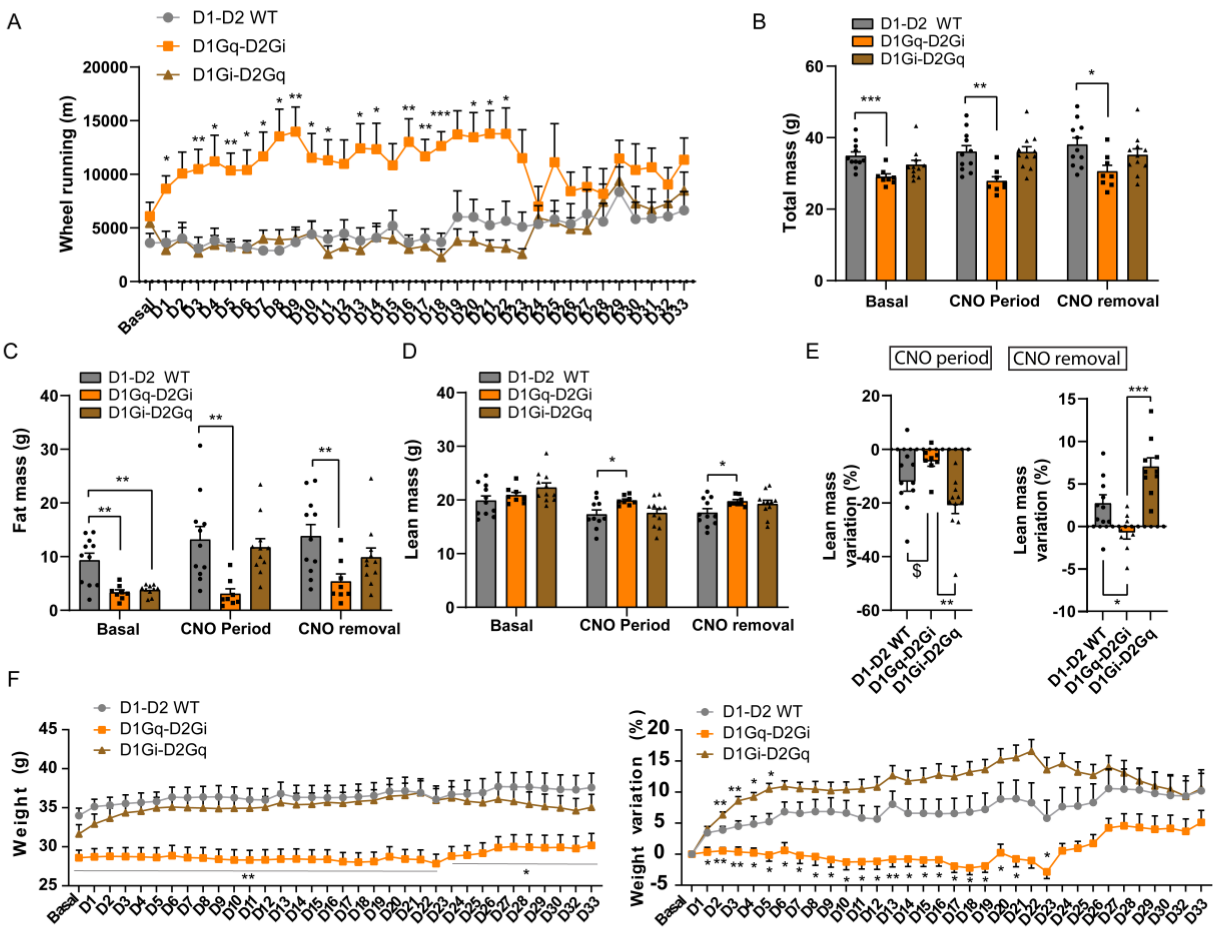

Suppl. Figure 5 (relates to Figure 5):

Distance ran (A), mass at the end of the CNO period (B), fat mass at the end of the CNO period (C), lean mass at the end of the CNO period (D), lean mass variations at the end of the CNO period and after CNO removal (E), weight and weight variation (F) for concomitant chemogenetic manipulations of D1- and D2-neurons (WT n=9, D1Gq-D2Gi n=8, D1Gi-D2Gq n=9). $: 0.05<p<0.1; * : p<0.05 ; ** : p<0.01; Error bars = s.e.m. Detailed statistics are displayed in Supplementary Table 2. Source data are provided as a Source Data file.

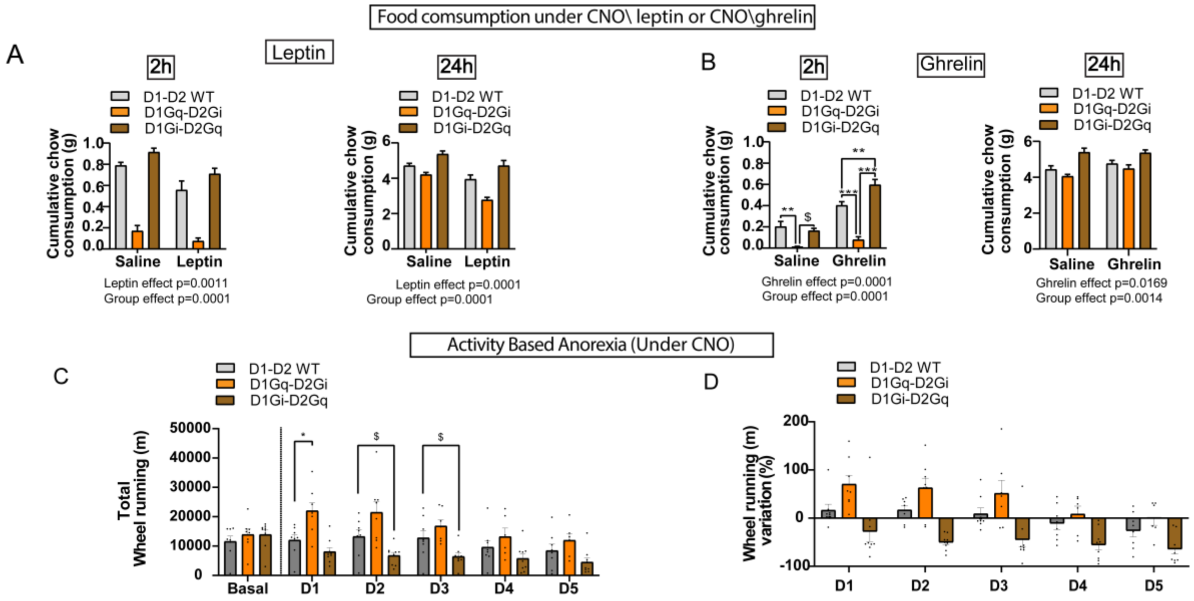

Suppl. Figure 6 (relates to Figure 6):

(A) Cumulative chow consumption under leptin administration in saline or CNO conditions under concomitant chemogenetic manipulations of D1- and D2-neurons measured after 2 hours (left) or 24 hours (right) (WT n=8, D1Gq-D2Gi n=7, D1Gi-D2Gq n=7). (B) Cumulative chow consumption under ghrelin administration in saline or CNO conditions under concomitant chemogenetic manipulations of D1- and D2-neurons measured after 2 hours (left) or 24 hours (right) (WT n=8, D1Gq-D2Gi n=7, D1Gi-D2Gq n=7). (C-D) total distance ran (C) and wheel running variation (D) over the ABA procedure under concomitant chemogenetic manipulations of D1- and D2-neurons (WT n=8, D1Gq-D2Gi n=8, D1Gi-D2Gq n=9). $: 0.05<p<0.1; * : p<0.05 ; ** : p<0.01; Error bars = s.e.m. Detailed statistics are displayed in Supplementary Table 2. Source data are provided as a Source Data file.

Supplementary table 1: Key resources

REAGENT or RESOURCE	SOURCE	IDENTIFIER
**Antibodies**		
Primary antibody anti-DS red (rabbit)	Takara Bio	632496
Primary antibody anti-GFP (mouse)	Clontech	632280
Primary antibody anti-Choline acetyl transferase (goat)	Merck Millipore	AB144P
Secondary antibody anti-rabbit alexa 568 (donkey)	ThermoFisher	A-10042
Secondary antibody anti-rabbit alexa 488 (donkey)	ThermoFisher	A-21206
Secondary antibody anti-goat alexa 568 (donkey)	ThermoFisher	A-11057
Secondary antibody anti-goat alexa 488 (donkey)	ThermoFisher	A-11055
**Bacterial and Virus Strains**		
AAV8-hSyn1-DIO-EGFP	University of Zurich	v115
AAV8-hSyn1-dFRT-mCherry	University of Zurich	v188
AAV8-hSyn-DIO-hM4D(Gi)-mCherry	Addgene	44362-AAV8
AAV8-hSyn-DIO-hM4D(Gi)-mCherry	University of Zurich	V84
AAV8-hSyn-DIO-hM3D(Gq)-mCherry	Addgene	44361-AAV8
AAV8-hSyn-DIO-hM3D(Gq)-mCherry	University of Zurich	V89
AAV8-hSyn1-dFRT-hM4D(Gi)-mCherry(rev)-dFRT-WPRE-hGHp(A)	University of Zurich	V189
AAV8-hSyn1-dFRT-hM3D(Gq)-mCherry(rev)-dFRT-WPRE-hGHp(A)	University of Zurich	V190
AAV8-hSyn1-dlox-jGCaMP8m(rev)-dlox-WPRE-SV40p(A)	University of Zurich	V628
AAV9-hSyn1-chl-dLight1.3b-WPRE-bGHp(A)	University of Zurich	v565
**Chemicals, Peptides, and Recombinant Proteins**		
Clozapine-N-Oxide (CNO)	ENZO	BML-NS105
Recombinant Mouse Leptin Protein, CF	Bio-Techne	498-OB-05M
Ghrelin	Bachem	H-4862.1000
JHU37160 dihydrochloride (J60 DREADD ligand)	hellobio	HB6261
**Critical Commercial Assays**		
ACCU-CHEK Performa	Roche	N/A
Fiber Optic Cannulae 0,5 NA, 400µm flat tip, L:6mm	RWD	N/A
Acylated Ghrelin (mouse, rat) Express ELISA kit	Bertin Bioreagent	A05117
Mouse Leptin ELISA Kit - 96-Well Plate	Sigma Aldrich - Merck	EZML-82K
**Experimental Models: Organisms/Strains**		
Mouse: C57BL/6J	Janvier Labs	N/A
Mouse: Tg(Drd1-cre)FK150Gsat/Mmucd	GENSAT	RRID: MMRRC_029178-UCD
Mouse: Tg(Drd2-cre)ER44Gsat/Mmucd	GENSAT	RRID: MMRRC_017263-UCD
Mouse D1Flp (D1 expressing flippase and YFP)	Produced by the Laboratory of B. Giros	N/A

Supplementary table 2: Summary of statistical analyses

Data	Statistical test	n	Outcome measure	P value
Figure 1				
Figure 1C	Two-way RM ANOVA	D2 cre mice:<br>Dreadd Gi = 9<br>Dreadd Gq = 7	Lever presses	Virus effect F(1, 14)=6.604 p=0.0222<br>CNO effect F(1, 14)=0.3902 p=0.5423<br>Interaction F(1, 14)=19.29 p=0.0006<br>Bonferroni's multiple comparisons test:<br>DREADD Gi: Sal vs CNO p=0.0258<br>DREADD Gq: Sal vs CNO p=0.0096
Figure 1D	Two-way RM ANOVA	D2 cre mice:<br>Dreadd Gi = 9<br>Dreadd Gq = 7	Breakpoint	Virus effect F(1, 14)=6.773 p=0.0209<br>CNO effect F(1, 14)=0.04977 p=0.8267<br>Interaction F(1, 14)=14.92 p=0.0017<br>Bonferroni's multiple comparisons test:<br>DREADD Gi: Sal vs CNO p=0.0310<br>DREADD Gq: Sal vs CNO p=0.0327
Figure 1E	Log-rank (Mantel-Cox) test	D2 cre mice:<br>Dreadd Gi = 9	Survival Curve	χ2=4.511 ; p value=0.0337
Figure 1E	Gehan-Breslow-Wilcoxon test	D2 cre mice:<br>Dreadd Gq = 7	Survival Curve	χ2=6.338 ; p value=0.0118
Figure 1F	Two-way Mixed-effects model ANOVA (Geisser-Greenhouse's epsilon correction)	D2 cre mice:<br>Dreadd Gi = 9	Rate (presses/s)	Ratio effect F(3.138, 25.10)=20.67 p=0.0001<br>(ε correction: 0.3922)<br>CNO effect F(1.00, 8.00)=0.1515 p=0.7073<br>(ε correction: 1.000)<br>Interaction F(3.379, 27.03)=1.024 p=0.4040<br>(ε correction: 0.4224)
Figure 1F	Two-way Mixed-effects model ANOVA (Geisser-Greenhouse's epsilon correction)	D2 cre mice:<br>Dreadd Gq = 7	Rate (presses/s)	Ratio effect F(1.245, 7.469)=4.953 p=0.0539<br>(ε correction: 0.1556)<br>CNO effect F(1.000, 6.000)=2.183 p=0.1900<br>(ε correction: 1.000)<br>Interaction F(2.468, 12.34)=0.7316 p=0.5278<br>(ε correction: 0.3085)
Figure 1H	Two-way RM ANOVA	D1 cre mice:<br>Dreadd Gi = 9<br>Dreadd Gq =8	Lever presses	Virus effect F(1, 15)=1.992 p=0.1785<br>CNO effect F(1, 15)=28.22 p=0.0001<br>Interaction F(1, 15)=1.658 p=0.2174
Figure 1I	Two-way RM ANOVA	D1 cre mice:<br>Dreadd Gi = 9<br>Dreadd Gq =8	Breakpoint	Virus effect F(1, 15)=0.06963 p=0.7955<br>CNO effect F(1, 15)=25.29 p=0.0001<br>Interaction F(1, 15)=0.2090 p=0.6541
Figure 1J	Log-rank (Mantel-Cox) test	D1 cre mice:<br>Dreadd Gi = 9	Survival Curve	χ2=8.910 ; p value=0.0028
Figure 1J	Log-rank (Mantel-Cox) test	D1 cre mice:<br>Dreadd Gq = 8	Survival Curve	χ2=3.652 ; p value=0.0560
Figure 1K	Two-way Mixed-effects model ANOVA (Geisser-Greenhouse's epsilon correction)	D1 cre mice:<br>Dreadd Gi = 9	Rate (presses/s)	Ratio effect F(2.171, 17.37)=17.20 p=0.0001<br>(ε correction: 0.2713)<br>CNO effect F(1, 8)=1.422 p=0.2673<br>(ε correction: 1.000)<br>Interaction F(2.167, 14.90)=1.135 p=0.3517<br>(ε correction: 0.2709)

Figure 1K	Two-way Mixed-effects model ANOVA (Geisser-Greenhouse's epsilon correction)	D1 cre mice:<br>Dreadd Gq = 8	Rate (presses/s)	Ratio effect F(1.361, 9.525)=9.687 p=0.0001<br>(ε correction: 0.1944)<br>CNO effect F(1, 7)=0.3425 p=0.5768<br>(ε correction: 1.000)<br>Interaction F(2.055, 13.80)=1.131 p=0.3605<br>(ε correction: 0.2936)
Figure 2				
Figure 2B	Paired T-test (two-tailed)	D2 cre mice<br>Dreadd Gi = 7	Number of licks	t=3.232, df=6, p=0.0179
Figure 2B	Paired T-test (two-tailed)	D2 cre mice<br>Dreadd Gq = 8	Number of licks	t=2.761, df=7, p=0.0281
Figure 2B	Paired T-test (two-tailed)	D1 cre mice<br>Dreadd Gi = 6	Number of licks	t=3.798, df=5, p=0.0127
Figure 2B	Paired T-test (two-tailed)	D1 cre mice<br>Dreadd Gq = 9	Number of licks	t=5.236, df=8, p=0.0008
Figure 2C	Two-way ANOVA	D1 cre mice = 5<br>(events =528)<br>D2 cre mice = 5<br>(events =591)	GCaMP CS Area under the curve	Time effect F(1, 2234)=35.43 p<0.0001<br>Group effect F(1, 2234)=2.236 p=0.1349<br>Interaction F(1, 2234)=1.342 p=0.2468
Figure 2C	Two-way ANOVA	D1 cre mice = 5<br>(events =528)<br>D2 cre mice = 5<br>(events =591)	GCaMP CS Average Zscore	Time effect F(1, 2234)=35.21 p=0.0001<br>Group effect F(1, 2234)=2.404 p=0.1211<br>Interaction F(1, 2234)=1.450 p=0.2286
Figure 2D	Two-way ANOVA	D1 cre mice = 5<br>(events =593)<br>D2 cre mice = 5<br>(events =660)	GCaMP lick Area under the curve	Time effect F(1, 2502)=303.0 p=0.0001<br>Group effect F(1, 2502)=9.477 p=0.0021<br>Interaction F(1, 2502)=1.301 p=0.2542
Figure 2D	Two-way ANOVA	D1 cre mice = 5<br>(events =593)<br>D2 cre mice = 5<br>(events =660)	GCaMP lick Average Zscore	Time effect F(1, 2502)=302.5 p=0.0001<br>Group effect F(1, 2502)=4.301 p=0.0382<br>Interaction F(1, 2502)=5.141 p=0.0235<br>Bonferroni's multiple comparisons test:<br>-2-0s: D1 vs D2 p=0.0043<br>0-2s: D1 vs D2 p=0.9882
Figure 2E	Two-way RM ANOVA	D1 cre mice = 4<br>events Saline = 252<br>events CNO = 168	dLight CS Area under the curve	Time effect F(1, 418)=308.5 p=0.0001<br>CNO effect F(1, 418)=1.019 p=0.3134<br>Interaction F (1, 418) = 0.5410 p=0.4624
Figure 2E	Two-way RM ANOVA	D1 cre mice = 4<br>events Saline = 252<br>events CNO = 168	dLight CS Average Zscore	Time effect F(1, 418)=311.3 p=0.0001<br>CNO effect F(1, 418)=0.9687 p=0.3256<br>Interaction F(1, 418)=0.4511 p=0.5022
Figure 2F	Two-way RM ANOVA	D1 cre mice = 4<br>events Saline = 271<br>events CNO = 186	dLight lick Area under the curve	Time effect F(1, 455)=4.083 p=0.0439<br>CNO effect F(1, 455)=4.010 p=0.0458<br>Interaction F(1, 455)=2.429 p=0.1198
Figure 2F	Two-way RM ANOVA	D1 cre mice = 4<br>events Saline = 271<br>events CNO = 186	dLight lick Average Zscore	Time effect F(1, 455)=2.457 p=0.1177<br>CNO effect F(1, 455)=3.798 p=0.0519<br>Interaction F(1, 455)=2.241 p=0.1351
Figure 2G	Two-way RM ANOVA	D2 cre mice = 3<br>events Saline = 170<br>events CNO = 105	dLight CS Area under the curve	Time effect F(1, 273)=200.2 p=0.0001<br>CNO effect F(1, 273)=1.849 p=0.1751<br>Interaction F(1, 273)=2.151 p=0.1436
Figure 2G	Two-way RM ANOVA	D2 cre mice = 3<br>events Saline = 170	dLight CS Average Zscore	Time effect F(1, 273)=203.5 p=0.0001<br>CNO effect F(1, 273)=1.887 p=0.1706

		events CNO = 105		Interaction F(1, 273)=2.355 p=0.1260
Figure 2H	Two-way RM ANOVA	D2 cre mice = 3<br>events Saline = 192<br>events CNO = 112	dLight LICK Area under the curve	Time effect F(1, 302)=82.15 p=0.0001<br>CNO effect F(1, 302)=31.40 p=0.0001<br>Interaction F(1, 302)=35.76 p=0.0001<br>Bonferroni's multiple comparisons test:<br>-2 0s: Sal vs CNO p=0.9999<br>0 2s: Sal vs CNO p=0.0001
Figure 2H	Two-way RM ANOVA	D2 cre mice = 3<br>events Saline = 192<br>events CNO = 112	dLight LICK Average Zscore	Time effect F(1, 302)=79.18 p=0.0001<br>CNO effect F(1, 302)=32.06 p=0.0001<br>Interaction F(1, 302)=36.33 p=0.0001<br>Bonferroni's multiple comparisons test:<br>-2 0s: Sal vs CNO p=0.9999<br>0 2s: Sal vs CNO p=0.0001
Figure 3				
Figure 3A	Two-way Mixed-effect model (Satterthwaite correction for degrees of freedom)	D1 Cre = 5<br>D2 Cre = 5<br>12 trials per animals	GCaMP Calcium activity during feeding trials (Zscore; D1 vs D2)	Intercept effect F(1, 12.499)=197.66 p=4.97e-9<br>Genotype effect F(1, 12.499)=0.1867 p=0.6731<br>Time effect F(1, 37.133)=4.1772 p=0.0481<br>Genotype x Time F(1, 37.133)=10.665 p=0.0024 post hoc-permutation test between genotypes
Figure 3A	Two-way RM ANOVA	D1 Cre = 5<br>events = 59<br>D2 Cre = 5<br>events = 60	GCaMP Calcium activity during feeding trials Area under the curve	Interaction F(1, 117)=19.88 p=0.0001<br>Time effect F(1, 117)=56.85 p=0.0001<br>Group effect F(1, 117)=7.376 p=0.0076<br>-40-0s: D1 vs D2 p=0.9999<br>0-60s: D1 vs D2 p=0.0001
Figure 3A	Two-way RM ANOVA	D1 Cre = 5<br>events = 59<br>D2 Cre = 5<br>events = 60	GCaMP Calcium activity during feeding trials Average Zscore	Interaction F(1, 117)=22.11 p=0.0001<br>Time effect F(1, 117)=18.37 p=0.0001<br>Group effect F(1, 117)=5.489 p=0.0208<br>-40-0s: D1 vs D2 p=0.9999<br>0-60s: D1 vs D2 p=0.0001
Figure 3A	Two-way RM ANOVA	D1 Cre = 5<br>events = 59<br>D2 Cre = 5<br>events = 60	GCaMP Calcium activity during feeding trials Peak Zscore	Interaction F(1, 117)=31.07 p=0.0001<br>Time effect F(1, 117)=186.1 p=0.0001<br>Group effect F(1, 117)=0.07555 p=0.7839<br>-40 0s: D1 vs D2 p=0.0099<br>0 60s: D1 vs D2 p=0.0391
Figure 3A	Two-way RM ANOVA	D1 Cre = 5<br>events = 59<br>D2 Cre = 5<br>events = 60	GCaMP Calcium activity during feeding trials Transient rate	Interaction F(1, 117)=12.67 p=0.0005<br>Time effect F(1, 117)=47.33 p=0.0001<br>Group effect F(1, 117)=0.2608 p=0.6105<br>-40 0s: D1 vs D2 p=0.0089<br>0 60s: D1 vs D2 p=0.0654
Figure 3B	Mixed-effect model (Satterthwaite correction for degrees of freedom)	D1GCaMP = 5<br>D2GCaMP = 5<br>12 trials per animals<br>5 events per trials	GCaMP Calcium activity during wheel running trials (Zscore D1 vs D2)	Intercept effect F(1, 9.998)=138.675 p=6.15e-7<br>Genotype effect F(1, 9.998)=1.2689 p=0.2879<br>Time effect F(1, 20.362)=0.6199 p=0.4402<br>Genotype x Time F(1, 20.362)=0.4579 p=0.5062

				(post hoc-permutation test between genotypes)
Figure 3B	Two-way RM ANOVA	D1 Cre = 5<br>events = 60<br>D2 Cre = 5<br>events = 59	GCaMP Calcium activity during wheel running trials Area under the curve	Interaction F(1, 117)=16.73 p=0.0001<br>Time effect F(1, 117)=96.48 p=0.0001<br>Group effect F(1, 117)=7.219 p=0.0083<br>-40-0s: D1 vs D2 p=0.9999<br>0-60s: D1 vs D2 p=0.0001
Figure 3B	Two-way RM ANOVA	D1 Cre = 5<br>events = 60<br>D2 Cre = 5<br>events = 59	GCaMP Calcium activity during wheel running trials Average Zscore	Interaction F(1, 117)=18.22 p=0.0001<br>Time effect F(1, 117)=51.69 p=0.0001<br>Group effect F(1, 117)=5.598 p=0.0196<br>-40-0s: D1 vs D2 p=0.9999<br>0-60s: D1 vs D2 p=0.0001
Figure 3B	Two-way RM ANOVA	D1 Cre = 5<br>events = 60<br>D2 Cre = 5<br>events = 59	GCaMP Calcium activity during wheel running trials Peak Zscore	Interaction F(1, 117)=1.616 p=0.2062<br>Time effect F(1, 117)=56.99 p=0.0001<br>Group effect F(1, 117)=0.1438 p=0.7052
Figure 3B	Two-way RM ANOVA	D1 Cre = 5<br>events = 60<br>D2 Cre = 5<br>events = 59	GCaMP Calcium activity during wheel running trials Transient rate	Interaction F(1, 118)=3.456 p=0.0655<br>Time effect F(1, 118)=60.18 p=0.0001<br>Group effect F (1, 118)=0.004899 p=0.9443
Figure 3D	Two-way RM ANOVA	D2Gi = 7<br>D2Gq = 8	Chow consumption (ratio CNO/SAL)	Infusion effect F(1, 13)=20.70 p=0.0005<br>Virus effect F(1, 13)=0.0001217 p=0.9914<br>Interaction F(1, 13)=0.3641 p=0.5566
Figure 3D	Two-way RM ANOVA	D2Gi = 7<br>D2Gq = 8	Wheel running activity<br>(ratio CNO/SAL)	Infusion effect F(1, 13)=0.3494 p=0.5646<br>Virus effect F(1, 13)=25.27 p=0.0002<br>Interaction F(1, 13)=48.01 p=0.0001<br>Bonferroni's multiple comparisons test:<br>D2Gi vs D2Gq: Pre infusion p>0.9999; Post infusion p=0.0001<br>Pre infusion vs Post infusion : D2Gi p=0.0016 D2Gq p=0.0002
Figure 3E	Two-way RM ANOVA	D1Gi = 9<br>D1Gq = 9<br>1 D1Gi and 1 D2Gq outlier values were excluded with Grubbs' method (Alpha = 0.01)	Chow consumption (ratio CNO/SAL)	Infusion effect F(1, 14)=0.4893 p=0.4957<br>Virus effect F(1, 14)=9.012 p=0.0095<br>Interaction F(1, 14)=8.166 p=0.0127<br>Bonferroni's multiple comparisons test:<br>D1Gi vs D1Gq: Pre infusion p>0.9999; Post infusion p=0.0006<br>Pre infusion vs Post infusion : D1Gq p=0.0495
Figure 3E	Two-way RM ANOVA	D1Gi = 9<br>D1Gq = 9<br>1 D1Gi and 1 D2Gq outlier values were excluded with Grubbs' method (Alpha = 0.01)	Wheel running activity<br>(ratio CNO/SAL)	Infusion effect F(1, 14)=1.103 p=0.3115<br>Virus effect F(1, 14)=7.508 p=0.0160<br>Interaction F(1, 14)=15.14 p=0.0016<br>Bonferroni's multiple comparisons test:<br>D1Gi vs D1Gq: Pre infusion p=0.9766; Post infusion p=0.0001<br>Pre infusion vs Post infusion : D1Gq p=0.0072
Figure 4				
Figure 4B	Two-way Mixed-effects model ANOVA	D2 WT = 9<br>D2 GI = 8	Food consumption D1-9; D24-25	Time effect F(3.466, 79.71)=70.40 p=0.0001<br>(ε correction: 0.3851)

	(Geisser-Greenhouse's epsilon correction)	D2 Gq = 9		Virus effect F(2, 23)=0.6509 p=0.5309<br>Interaction F(18, 207)=4.489 p=0.0001<br>Bonferroni's multiple comparisons test:<br>D2Gq vs D2WT: Day3 p=0.0091; Day25 p=0.0330<br>D2Gq vs D2Gi: Day1 p=0.0256; Day24 p=0.0197; Day25 p=0.0312
Figure 4B	One-way ANOVA	D2 WT = 9<br>D2 GI = 8<br>D2 Gq = 9	Food consumption mean D10-D22	F(2, 23)=0.6708 p=0.5210
Figure 4B	One-way ANOVA	D2 WT = 9<br>D2 GI = 8<br>D2 Gq = 9	Food consumption mean D26-D33	F(2, 23)=1.567 p=0.2302
Figure 4C	Two-way Mixed-effects model ANOVA (Geisser-Greenhouse's epsilon correction)	D2 WT = 7<br>D2 GI = 7<br>D2 Gq = 8<br>(2 WT, 1 D1-Gi and 1 D1-Gq animals were exposed to a running wheel without recording)	Running wheel variation<br>D1-9; D24-25	Time effect F(3.868, 72.36)=4.052 p=0.0055 (ε correction: 0.2276)<br>Virus effect F(2, 19)=1.659 p=0.2167<br>Interaction F(34, 318)=3.536 p=0.0001<br>Bonferroni's multiple comparisons test:<br>D2Gi vs D2WT: Day1 p=0.0272<br>D2Gq vs D2WT: Day11 p=0.0305; Day13 p=0.0038; Day15 p=0.0208; Day19 p=0.0328 ; Day21 p=0.0145 ; Day23 p=0.0228
Figure 4D	One-way ANOVA	D2 WT = 9<br>D2 GI = 8<br>D2 Gq = 9	Total mass variation CNO period	F(2, 23)=0.3597 p=0.7017
Figure 4D	Brown-Forsythe One-way ANOVA test	D2 WT = 9<br>D2 GI = 8<br>D2 Gq = 9<br>1 D2 WT outlier value was removed with Grubbs' method (Alpha = 0.01)	Fat mass variation CNO period	F(2.00, 10.99)=5.966 p=0.0176<br>Dunnett's T3 multiple comparisons test<br>D1 WT vs. D1 Gi p=0.0026<br>D1 WT vs. D1 Gq p=0.0447
Figure 4D	One-way ANOVA	D2 WT = 9<br>D2 GI = 8<br>D2 Gq = 9	Total mass variation CNO removal	F(2, 23)=12.67 p=0.0002<br>Bonferroni's multiple comparisons test:<br>D2WT vs D2Gq p=0.0002<br>D2Gi vs D2Gq p=0.0040
Figure 4D	One-way ANOVA	D2 WT = 9<br>D2 GI = 8<br>D2 Gq = 9	Fat mass variation CNO removal	F(2, 23)=8.537 p=0.0017<br>Bonferroni's multiple comparisons test:<br>D2WT vs D2Gq p=0.0020<br>D2Gi vs D2Gq p=0.0184
Figure 4E	Two-way Mixed-effects model ANOVA (Geisser-Greenhouse's epsilon correction)	D1 WT = 12<br>D1 GI = 11<br>D1 Gq = 12<br>1 D1WT and 1D1Gq outlier values removed with Grubbs' method (Alpha = 0.01)	Food consumption D1-9; D24-25	Time effect F(3.418, 108.4)=34.19 p=0.0001 (ε correction: 0.4883)<br>Virus effect F(2, 32)=1.470 p=0.2451<br>Interaction F(14, 222)=6.702 p=0.0001<br>Bonferroni's multiple comparisons test:<br>D1Gq vs D1WT: Day1 p=0.0183; Day3 p=0.0771; Day5 p=0.0612; Day7 p=0.0411

				D1Gq vs D1Gi: Day1 p=0.0022; Day5 p=0.0096; Day24 p=0.0082; Day25 p=0.0558
Figure 4E	One-way ANOVA	D1 WT = 12<br>D1 GI = 11<br>D1 Gq = 12	Food consumption mean D10-D22	F(2, 32)=6.579 p=0.0040<br>Bonferroni's multiple comparisons test:<br>D1Gq vs D1WT: p=0.0152<br>D1Gq vs D1Gi: p=0.0084
Figure 4E	One-way ANOVA	D1 WT = 12<br>D1 GI = 11<br>D1 Gq = 12	Food consumption mean D26-D33	F(2, 32)=2.153 p=0.1327
Figure 4F	Two-way Mixed-effects model ANOVA (Geisser-Greenhouse's epsilon correction)	D1 WT = 9<br>D1 GI = 11<br>D1 Gq = 11<br>(3 WT and 1 D1-Gq animals were exposed to a running wheel without recording)	Running wheel variation<br>D1-9; D24-25	Time effect F(4.651, 128.9)=10.12 p=0.0001 (ε correction: 0.2736)<br>Virus effect F(2, 28)=11.07 p=0.0003<br>Interaction F(34, 471)=3.334 p=0.0001<br>Bonferroni's multiple comparisons test:<br>D1Gq vs D1WT: Day1 p=0.0531; Day3 p=0.0223; Day5 p=0.0129; Day7 p=0.0218; Day9 p=0.0213; Day11 p=0.0086; Day13 p=0.0290; Day15 p=0.0285<br>D1Gi vs D1WT: Day1 p=0.0299; Day17 p=0.0187; Day19 p=0.0703; Day21 p=0.0174; Day25 p=0.0223; Day27 p=0.0770; Day29 p=0.0090; Day31 p=0.0325; Day33 p=0.0808
Figure 4G	Brown-Forsythe ANOVA test	D1 WT = 12<br>D1 GI = 11<br>D1 Gq = 12	Total mass variation CNO period	F (2.00, 20.08) =3.861<br>P=0.0381<br>Dunnett's T3 multiple comparisons test<br>D1 WT vs. D1 Gi p=0.3297<br>D1 WT vs. D1 Gq p=0.4184<br>D1 Gi vs. D1 Gq p=0.0826
Figure 4G	One-way ANOVA	D1 WT = 12<br>D1 GI = 11<br>D1 Gq = 12	Fat mass variation CNO period	F (2, 32) =3.492 P=0.0425<br>Bonferroni's multiple comparisons test:<br>D1Gi vs D1Gq p=0.0463
Figure 4G	One-way Brown-Forsythe ANOVA test	D1 WT = 12<br>D1 GI = 11<br>D1 Gq = 12<br>1 D1 Gq and 1D1 GI outlier values were removed<br>with Grubbs' method (Alpha = 0.01)	Total mass variation CNO removal	F (2.00, 17.99) =6.066 P=0.0097<br>Dunnett's T3 multiple comparisons test<br>D1 WT vs. D1 Gi p=0.9612<br>D1 WT vs. D1 Gq p=0.0044<br>D1 Gi vs. D1 Gq p=0.0478
Figure 4G	One-way Kruskal-Wallis test	D1 WT = 12<br>D1 GI = 11<br>D1 Gq = 12<br>1 D1 Gq and 1D1 GI outlier values were removed<br>with Grubbs' method (Alpha = 0.01)	Fat mass variation CNO removal	Kruskal-Wallis statistic: 9.225; p=0.0099<br>Dunn's multiple comparisons test<br>D1WT vs D1Gq p=0.0139<br>D1Gi vs D1Gq p=0.0544

Figure 5				
Figure 5B	Two-way Mixed-effects model ANOVA (Geisser-Greenhouse's epsilon correction)	D1-D2 WT = 11<br>D1Gq-D2Gi = 8<br>D1Gi-D2Gq = 11	Food consumption D1-9; D24-25	Time effect F(4.272, 115.3)=32.84 p=0.0001 (ε correction: 0.6103)<br>Virus effect F(2, 27)=0.3648 p=0.6977<br>Interaction F(14, 189)=7.911 p=0.0001<br>Bonferroni's multiple comparisons test:<br>WT vs D1GqD2Gi: Day1 p=0.0498; Day24 p=0.0056; Day25 p=0.0769<br>WT vs D1GiD2Gq: Day3 p=0.0648; Day24 p=0.0967<br>D1GiD2Gq vs D1GqD2Gi: Day1 p=0.0057; Day3 p=0.0277; Day24 p=0.0004; Day25 p=0.0001
Figure 5B	One-way ANOVA	D1-D2 WT = 11<br>D1Gq-D2Gi = 8<br>D1Gi-D2Gq = 11	Food consumption mean D10-22	F(2, 27)=1.880 p=0.1720
Figure 5B	One-way ANOVA	D1-D2 WT = 11<br>D1Gq-D2Gi = 8<br>D1Gi-D2Gq = 11	Food consumption mean D26-33	F(2, 27)=1.425 p=0.2580
Figure 5C	Two-way Mixed-effects model ANOVA (Geisser-Greenhouse's epsilon correction)	D1-D2 WT = 9<br>D1Gq-D2Gi = 8<br>D1Gi-D2Gq = 9<br>(2 WT and 2 D1Gq-D2Gi animals were exposed to a running wheel without recording)	Running wheel variation	Time effect F(2.425, 53.64)=2.841 p=0.0573 (ε correction: 0.1427)<br>Virus effect F(2, 23)=8.839 p=0.0014<br>Interaction F(34, 376)=3.486 p=0.0001<br>Bonferroni's multiple comparisons test:<br>WT vs D1Gq-D2Gi: Day1 p=0.0791; Day3 p=0.0122; Day5 p=0.0013; Day7 p=0.0148; Day9 p=0.0105; Day13 p=0.0106; Day15 p=0.0164; Day17 p=0.0059; Day19 p=0.0010<br>D1Gi-D2Gq vs WT: Day1 p=0.0966; Day3 p=0.0600; Day5 p=0.0005; Day11 p=0.0336; Day13 p=0.0039; Day15 p=0.0331; Day17 p=0.0047; Day19 p=0.0282; Day21 p=0.0568; Day23 p=0.0443
Figure 5D	One-way ANOVA	D1-D2 WT = 11<br>D1Gq-D2Gi = 8<br>D1Gi-D2Gq = 11	Total mass variation CNO period (D1-D23)	F(2, 27)=9.421 p=0.0008<br>Bonferroni's multiple comparisons test:<br>WT vs D1Gi-D2Gq: p=0.0638<br>D1Gq-D2Gi vs D1Gi-D2Gq p=0.0006
Figure 5D	One-waye Kruskal-Wallis test	D1-D2 WT = 11<br>D1Gq-D2Gi = 8<br>D1Gi-D2Gq = 11<br>1 D1Gq-D2Gi outlier value was removed with Grubbs' method (Alpha = 0.01)	Fat mass variation CNO period (D1-D23)	Kruskal-Wallis statistic: 16.74 p=0.0002<br>Dunn's multiple comparisons test<br>WT vs D1Gq-D2Gi p=0.0408<br>D1Gq-D2G vs D1Gi-D2Gq p=0.0001
Figure 5D	One-way ANOVA	D1-D2 WT = 11<br>D1Gq-D2Gi = 8<br>D1Gi-D2Gq = 11	Total mass variation CNO removal (D23-D36)	F(2, 27)=19.79 p=0.0001<br>Bonferroni's multiple comparisons test:<br>WT vs D1Gi-D2Gq p=0.0004<br>D1Gq-D2Gi vs D1Gi-D2Gq p=0.0001

Figure 5D	One-way ANOVA	D1-D2 WT = 11<br>D1Gq-D2Gi = 8<br>D1Gi-D2Gq = 11	Fat mass variation CNO removal (D23-D36)	F(2, 27)=52.97 p=0.0001<br>Bonferroni's multiple comparisons test:<br>WT vs D1Gi-D2Gq p=0.0171<br>WT vs D1Gq-D2Gi p=0.0001<br>D1Gq-D2Gi vs, D1Gi-D2Gq p=0.0001
Figure 6				
Figure 6A	Two-way Mixed-effects model ANOVA (Geisser-Greenhouse's epsilon correction)	D1-D2 WT = 11<br>D1Gq-D2Gi = 8<br>D1Gi-D2Gq = 11	Total mass variation (Food restricted procedure)	Time effect F(1.421, 38.36)=348.2 p=0.0001<br>(ε correction: 0.2368)<br>Virus effect F(2, 27)=4.600 p=0.0191<br>Interaction F(12, 162)=10.63 p=0.0001<br>Bonferroni's multiple comparisons test:<br>WT vs D1Gq-D2Gi: 1.5g(Day5) p=0.0467; 1.5g(Day6) p=0.0212
Figure 6B	Two-way Mixed-effects model ANOVA (Geisser-Greenhouse's epsilon correction)	D1-D2 WT = 9<br>D1Gq-D2Gi = 8<br>D1Gi-D2Gq = 9<br>(2 WT and 2 D1Gq-D2Gi animals were exposed to a running wheel without recording)<br>1 outlier value removed<br>with Grubbs' method (Alpha = 0.01)	Running wheel variation<br>(Food restricted procedure)	Time effect F(3.503, 79.39)=1.644 p=0.1784<br>(ε correction: 0.5838)<br>Virus effect F(2, 23)=7.484 p=0.0031<br>Interaction F(12, 136)=5.156 p=0.0001<br>Bonferroni's multiple comparisons test:<br>WT vs D1Gq-D2Gi: 3g p=0.0027; 2.5g p=0.0085; 2g p=0.0429; 1.5(1) p=0.0318; 1.5(3) p=0.0648
Figure 6C	Brown-Forsythe ANOVA test	D1-D2 WT = 11<br>D1Gq-D2Gi = 8<br>D1Gi-D2Gq = 11<br>1 D1Gi-D2Gq outlier value was removed with Grubbs' method (Alpha = 0.01)	Peripheral Leptin	F(2.00, 15.65)=10.99 p=0.0010<br>Dunnett's T3 multiple comparisons test<br>WT vs D1Gq-D2Gi: p=0.0038<br>WT vs D1Gi-D2Gq: p=0.1179<br>D1Gq-D2Gi vs D1Gi-D2Gq p=0.0088
Figure 6C	One-way ANOVA	D1-D2 WT = 9<br>D1Gq-D2Gi = 8<br>D1Gi-D2Gq = 9<br>(2 D1-D2 WT and 2 D1Gi-D2Gq animals samples were not detected by Elisa kit)<br>1 D1Gq-D2Gi outlier value was removed with Grubbs' method (Alpha = 0.01)	Peripheral Ghrelin	F(2, 22)=4.870 p=0.0177<br>Bonferroni's multiple comparisons test:<br>WT vs D1Gi-D2Gq p=0.0142
Figure 6C	One-way ANOVA	D1-D2 WT = 11<br>D1Gq-D2Gi = 8<br>D1Gi-D2Gq = 11	Weight	F(2, 29)=7.048 p=0.0032<br>Bonferroni's multiple comparisons test:<br>WT vs D1Gi-D2Gq p=0.0029<br>D1Gq-D2Gi vs D1Gi-D2Gq p=0.0463
Figure 6D	Two-way Mixed-effects model ANOVA (Geisser-	D1-D2WT= 8<br>D1Gq-D2Gi = 8<br>D1Gi-D2Gq = 9	Weight variation during ABA protocol	Time effect F(5, 105)=324.2 p=0.0001<br>(ε correction: 0.6460)<br>group effect F(2, 22)=17.45 p=0.0001

	Greenhouse's epsilon correction			Interaction F(10, 105)=7.702 p=0.0001<br>Bonferroni's multiple comparisons test:<br>D1-D2WT vs D1Gq-D2Gi: D2 p=0.0046; D3 p=0.0312; D5 p=0.0220<br>D1-D2WT vs D1GI-D2Gq: D2 p=0.0051; D3 p=0.0038; D4 p=0.0006; D5 p=0.0001
Figure 6E	Two-way Mixed-effects model ANOVA (Geisser-Greenhouse's epsilon correction	D1-D2WT= 8<br>D1Gq-D2Gi = 8<br>D1Gi-D2Gq = 9	Wheel running during ABA protocol	Time effect F(2.906, 61.29)=53.66 P=0.0001 (ε correction: 0.2642)<br>group effect F(2, 22)=7.078 p=0.0042<br>Interaction F(22, 232)=9.935 p=0.0001<br>Bonferroni's multiple comparisons test:<br>D1-D2WT vs D1Gq-D2Gi: D1 night p=0.0004 D2 night p=0.0051<br>D1-D2WT vs D1GI-D2Gq: D1 night p=0.0023; D2 night p=0.0001; D3 night p=0.0001; D4 night p=0.0190; D5 night p=0.0381
Figure 6F	Two-way Mixed-effects model ANOVA (Geisser-Greenhouse's epsilon correction	D1-D2WT= 8<br>D1Gq-D2Gi = 8<br>D1Gi-D2Gq = 9	Chow consumption during ABA protocol	Time effect F(2.492, 52.96)=192.8 p=0.0001 (ε correction: 0.6230)<br>group effect F(2, 22)=2.496 p=0.1055<br>Interaction F(8, 85)=0.4240 p=0.9037
Figure 6G	Log-rank (Mantel-Cox) test	D1-D2WT= 8<br>D1Gq-D2Gi = 8<br>D1Gi-D2Gq = 9	Survival Curve	D1-D2WT vs D1Gq-D2Gi: $\chi 2$= 2.358; p=0.1247<br>D1-D2WT vs D1Gi-D2Gq: $\chi 2$=1.125; p=0.2888<br>D1Gi-D2Gq vs D1Gq-D2Gi: $\chi 2$=5.583; p=0.0189
Supplementary figure				
Supplementary Figure 1				
Figure Supp 1B	Two-way Mixed-effects model ANOVA (Geisser-Greenhouse's epsilon correction)	D2 GI = 9<br>D2 Gq = 7<br>1 D2Gi outlier value removed with Grubbs' method (Alpha = 0.01)	Lever press (Learning)	Time effect F(1.121, 8.972)=139.2 p=0.0001 (ε correction: 0.3738)<br>lever effect F(1.545, 12.36)=177.2 p=0.0001 (ε correction: 0.5151)<br>Interaction F(1.299, 7.508)=46.80 p=0.0001 (ε correction: 0.1444)<br>Bonferroni's multiple comparisons test:<br>Reinforced Lever (L1): D1 Gi vs D1 Gq p=0.7850<br>Non reinforced Lever (L2): D1 Gi vs D1 Gq p=0.5598
Figure Supp 1C	Two-way RM ANOVA	D2 Gi = 8<br>D2 Gq= 7	Lick/burst	Virus effect F(1, 13)=0.04570 p=0.8340<br>CNO effect F(1, 13)=6.896 p=0.0209<br>Interaction F(1, 13)=0.03268 P=0.8593
Figure Supp 1D	Two-way RM ANOVA	D2 GI =8<br>D2 Gq = 7	RPM	Virus effect F(1, 13)=1.871 p=0.1946<br>CNO effect F(1, 13)=1.976 p=0.1833<br>Interaction F(1, 13)=0.3563 p=0.5608
Figure	Two-way RM ANOVA	D2 GI =9	Distance	Virus effect F(1, 15)=11.92 p=0.0036

Supp 1E		D2 Gq = 8		CNO effect F(1, 15)=1.319 p=0.2687<br>Interaction F(1, 15)=28 p=0.0001<br>Bonferroni's multiple comparisons test:<br>D2Gi: Sal vs CNO p=0.0006<br>D2Gq: Sal vs CNO p=0.0245
Figure Supp 1E	Two-way RM ANOVA	D2 GI =9<br>D2 Gq = 8	Mean speed	Virus effect F(1, 15)=11.91 p=0.0036<br>CNO effect F(1, 15)=1.309 p=0.2704<br>Interaction F(1, 15)=28.02 p=0.0001<br>Bonferroni's multiple comparisons test:<br>D2Gi: Sal vs CNO p=0.0006<br>D2Gq: Sal vs CNO p=0.0243
Figure Supp 1F	Two-way Mixed-effects model ANOVA (Geisser-Greenhouse's epsilon correction)	D1 GI = 9<br>D1 Gq = 8	Lever press (Learning)	Time effect F(1.104, 8.833)=103.7 p=0.0001 (ε correction: 0.3680)<br>lever effect F(1.555, 12.44)=110.4 p=0.0001 (ε correction: 0.5184)<br>Interaction F(1.925, 13.69)=45.22 p<0.0001 (ε correction: 0.2139)<br>Bonferroni's multiple comparisons test:<br>Reinforced Lever (L1): D1 Gi vs D1 Gq p=0.3512<br>Non reinforced Lever (L2): D1 Gi vs D1 Gq p>0.9999
Figure Supp 1G	Two-way RM ANOVA	D1 GI = 9<br>D1 Gq = 8	Lever presses CNO (2mg/kg)	Virus effect F(1, 15)=11.59 p=0.0039<br>CNO effect F(1, 15)=13.59 p=0.0022<br>Interaction F(1, 15)=0.3135 p=0.5838
Figure Supp 1G	Two-way RM ANOVA	D1 GI = 9<br>D1 Gq = 8	Break point CNO (2mg/kg)	Virus effect F(1, 15)=9.109 p=0.0086<br>CNO effect F(1, 15)=17.29 p=0.0008<br>Interaction F(1, 15)=0.00277 p=0.9587
Figure Supp 1G	Two-way Mixed-effects model ANOVA (Geisser-Greenhouse's epsilon correction)	D1 Gi = 9	Rate (presses/s) CNO (2mg/kg)	Ratio effect F(2.634, 21.07)=13.85 p=0.0001 (ε correction: 0.3292)<br>CNO effect F(1.00, 8.00)=0.8356 p=0.3874 (ε correction: 1.000)<br>Interaction F(4.193, 32.49)=1.724 p=0.1664 (ε correction: 0.5241)
Figure Supp 1G	Two-way Mixed-effects model ANOVA (Geisser-Greenhouse's epsilon correction)	D1 Gq= 8	Rate (presses/s) CNO (2mg/kg)	Ratio effect F(2.371, 16.60)=4.209 p=0.0281 (ε correction: 0.3534)<br>CNO effect F(1.000, 7.000)=15.63 p=0.0055 (ε correction: 1.000)<br>Interaction F(2.449, 16.65)=7.832 p=0.0039 (ε correction: 0.2854)<br>Bonferroni's multiple comparisons test:<br>Saline vs CNO: ratio 16 p=0.0757; ratio 32 p=0.0165; ratio 64 p=0.0139
Figure Supp 1H	Two-way RM ANOVA	D1 GI = 9<br>D1 Gq = 9	Lever presses J60 (1mg/kg)	Virus effect F(1, 16)=2.623 p=0.1249<br>J60 effect F(1, 16)=5.102 p=0.0382<br>Interaction F(1, 16)=0.2318 p=0.6367
Figure Supp 1H	Two-way RM ANOVA	D1 GI = 9<br>D1 Gq = 9	Break point J60 (1mg/kg)	Virus effect F(1, 16)=5.789 p=0.0286<br>J60 effect F(1, 16)=5.869 p=0.0277<br>Interaction F(1, 16)=0.07715 p=0.7848
Figure Supp 1I	Two-way RM ANOVA	D1 GI =11<br>D1 Gq = 10	Distance	Virus effect F(1, 19)=9.705 p=0.0057<br>CNO effect F(1, 19)=9.692 p=0.0057

				Interaction F(1, 19)=14.98 p=0.0010<br>Bonferroni's multiple comparisons test:<br>D1Gi: Sal vs CNO p=0.9999<br>D1Gq: Sal vs CNO p=0.0002
Figure Supp 1I	Two-way RM ANOVA	D1 GI =11<br>D1 Gq = 10	Mean speed	Virus effect F(1, 19)=9.675 p=0.0058<br>CNO effect F(1, 19)=9.690 p=0.0057<br>Interaction F(1, 19)=14.96 p=0.0010<br>Bonferroni's multiple comparisons test:<br>D1Gi: Sal vs CNO p=0.9999<br>D1Gq: Sal vs CNO p=0.0002
Suppl. Figure 1J	Two-way RM ANOVA	D1 Gi = 11<br>D1 Gq=10	Lick/burst	Virus effect F(1, 19)=3.917 p=0.0625<br>CNO effect F(1, 19)=2.378 p=0.1395<br>Interaction F(1, 19)=0.9080 p=0.3526
Figure Supp 1K	Two-way RM ANOVA	D1 Gi = 8<br>D1 Gq = 8	RPM	Virus effect F(1, 14)=0.04791 p=0.8299<br>CNO effect F(1, 14)=1.774 p=0.2042<br>Interaction F(1, 14)=0.05004 p=0.8262
Supplementary Figure 2				
Suppl. Figure 2B	Two-way Mixed-effects model ANOVA (Geisser-Greenhouse's epsilon correction)	D2 cre mice<br>Dreadd Gi = 7	Cumulative Chow consumption (1h - 4h)	Time effect F(1.672, 10.03)=2065 p=0.0001 (ε correction: 0.3345)<br>CNO effect F(1.000, 6.000)=32.69 p=0.0012 (ε correction: 1.000)<br>Interaction F(2.306, 13.84)=5.830 p=0.0122 (ε correction: 0.4612)<br>Bonferroni's multiple comparisons test: DREADD Gi Sal vs CNO: 4h p=0.0084; 12h p=0.0171 24h p=0.0416; 48h p=0.0455
Figure Supp 2B	Two-way Mixed-effects model ANOVA (Geisser-Greenhouse's epsilon correction)	D2 cre mice<br>Dreadd Gi = 7	Weight variation	Time effect F(1.000, 6.000)=19.95 p=0.0043 (ε correction: 1.000)<br>CNO effect F(1.000, 6.000)=7.204 p=0.0363 (ε correction: 1.000)<br>Interaction F(1.000, 6.000)=13.50 p=0.0104 (ε correction: 1.000)<br>Šídák's multiple comparisons test: DREADD Gi Sal vs CNO: 24h p=0.0500; 48h p=0.2485
Suppl. Figure 2B	Two-way Mixed-effects model ANOVA (Geisser-Greenhouse's epsilon correction)	D2 cre mice<br>Dreadd Gq = 8	Cumulative Chow consumption (1h - 4h)	Time effect F(1.278, 8.944)=975.3 p=0.0001 (ε correction: 0.2555)<br>CNO effect F(1.000, 7.000)=9.034 p=0.0198 (ε correction: 1.000)<br>Interaction F(2.182, 15.28)=21.06 p=0.0001 (ε correction: 0.4365)<br>Bonferroni's multiple comparisons test:<br>DREADD Gq Sal vs CNO: 48h p=0.0043
Suppl. Figure 2B	Two-way Mixed-effects model ANOVA (Geisser-Greenhouse's epsilon correction)	D2 cre mice<br>Dreadd Gq = 8	Weight variation	Time effect F(1.0, 7.0)=0.008117 p=0.9307 (ε correction: 1.000)<br>CNO effect F(1.000, 7.000)=26.40 p=0.0013 (ε correction: 1.000)<br>Interaction F(1.000, 7.000)=1.854 p=0.2155 (ε correction: 1.000)

Suppl. Figure 2B	Two-way Mixed-effects model ANOVA (Geisser-Greenhouse's epsilon correction)	D1 cre mice<br>Dreadd Gi = 9	Cumulative Chow consumption (1h - 48h)	Time effect $F(1.874, 14.99)=1514$ $p=0.0001$ (ε correction: 0.3747)<br>CNO effect $F(1.000, 8.000)=7.473$ $p=0.0257$ (ε correction: 1.000)<br>Interaction $F(1.795, 14.36)=0.6985$ $p=0.4986$ (ε correction: 0.3590)
Suppl. Figure 2B	Two-way Mixed-effects model ANOVA (Geisser-Greenhouse's epsilon correction)	D1 cre mice<br>Dreadd Gi = 9	Weight variation	Time effect $F(1.00, 8.00)=0.8960$ $p=0.3716$ (ε correction: 1.000)<br>CNO effect $F(1.00, 8.00)=0.6924$ $p=0.4295$ (ε correction: 1.000)<br>Interaction $F(1.000, 8.000)=3.569$ $p=0.0955$ (ε correction: 1.000)
Suppl. Figure 2B	Two-way Mixed-effects model ANOVA (Geisser-Greenhouse's epsilon correction)	D1 cre mice<br>Dreadd Gq = 9	Cumulative Chow consumption (1h - 48h)	Time effect $F(1.994, 15.95)=1729$ $p=0.0001$ (ε correction: 0.3987)<br>CNO effect $F(1.000, 8.000)=21.63$ $p=0.0016$ (ε correction: 1.000)<br>Interaction $F(1.638, 13.10)=2.277$ $p=0.1473$ (ε correction: 0.3275
Figure 2B	Two-way Mixed-effects model ANOVA (Geisser-Greenhouse's epsilon correction)	D1 cre mice<br>Dreadd Gq = 9	Weight variation	Time effect $F(1.00, 8.00)= 42.30$ $p=0.0002$ (ε correction: 1.000)<br>CNO effect $F(1.00, 8.00)=2.831$ $p=0.1310$ (ε correction: 1.000)<br>Interaction $F(1.00, 8.00)=0.008763$ $p=0.9771$ (ε correction: 1.00)
Suppl. Figure 2C	Two-way Mixed-effects model ANOVA (Geisser-Greenhouse's epsilon correction)	D2 cre<br>Dreadd Gi = 7	Fasting refeeding: Food consumption (1h - 24h)<br>CNO: 2mg/kg	Time effect $F(1.652, 9.912)=899.7$ $p=0.0001$ (ε correction: 0.4130)<br>CNO effect $F(1.000, 6.000)=15.96$ $p=0.0072$ (ε correction: 1.000)<br>Interaction $F(1.969, 11.82)=16.90$ $p=0.0004$ (ε correction: 0.4923)<br>Bonferroni's multiple comparisons test:<br>DREADD GI Sal vs CNO: 1h $p=0.0012$; 2h $p=0.0027$; 4h $p=0.0057$
Suppl. Figure 2C	Two-way Mixed-effects model ANOVA (Geisser-Greenhouse's epsilon correction)	D2 cre<br>Dreadd Gi =7	Fasting refeeding: Food consumption (1h - 24h)<br>CNO: 0.5mg/kg	Time effect $F(1.515, 9.089)=484.68$ $p=0.0001$ (ε correction: 0.3787)<br>CNO effect $F(1.00, 6.00)=0.1468$ $p=0.7148$ (ε correction: 1.000)<br>Interaction $F(1.00, 7.00)=7.520$ $p=0.0210$<br>Bonferroni's multiple comparisons test:<br>DREADD GI Sal vs CNO: 1h $p=0.028$; 2h $p=0.0099$
Suppl. Figure 2C	Two-way Mixed-effects model ANOVA (Geisser-Greenhouse's epsilon correction)	D2 cre<br>Dreadd Gq = 8	Fasting refeeding: Food consumption (1h - 24h)<br>CNO: 2mg/kg	Time effect $F(1.251, 8.754)=201.0$ $p=0.0001$ (ε correction: 0.3126)<br>CNO effect $F(1.000, 7.000)=17.16$ $p=0.0043$ (ε correction: 1.000)<br>Interaction $F(1.152, 8.067)=6.396$ $p=0.00321$ (ε correction: 0.2881)<br>Bonferroni's multiple comparisons test:<br>DREADD Gq Sal vs CNO: 24h $p=0.0370$

Suppl. Figure 2C	Two-way Mixed-effects model ANOVA (Geisser-Greenhouse's epsilon correction)	D2 cre<br>Dreadd Gq = 7<br>1 outlier value removed<br>with Grubbs' method (Alpha = 0.01)	Fasting refeeding:<br>Food consumption (1h - 24h)<br>CNO: 0.5mg/kg	Time effect F(1.246, 7.478)=104.9 p=0.0001 (ε correction: 0.3116)<br>CNO effect F(1.000, 6.000)=0.5173 p=0.4990 (ε correction: 1.000)<br>Interaction F(2.218, 13.31)=1.949 p=0.1787 (ε correction: 0.5545)
Suppl. Figure 2C	Two-way Mixed-effects model ANOVA (Geisser-Greenhouse's epsilon correction	D1 cre<br>Dreadd Gi = 9	Fasting refeeding:<br>Food consumption (1h - 24h)<br>CNO: 2mg/kg	Time effect F(1.181, 9.451)=414.5 p=0.0001 (ε correction: 0.2953)<br>CNO effect F(1.00, 8.00)=0.3249 p=0.5843 (ε correction: 1.000)<br>Interaction F(2.622, 20.32)=3.468 p=0.0400 (ε correction: 0.6556)
Suppl. Figure 2C	Two-way Mixed-effects model ANOVA (Geisser-Greenhouse's epsilon correction	D1 cre<br>Dreadd Gi = 8<br>1 outlier value removed<br>with Grubbs' method (Alpha = 0.01)	Fasting refeeding:<br>Food consumption (1h - 24h)<br>CNO: 0.5mg/kg	Time effect F(1.309, 9.163)=813.7 p=0.0001 (ε correction: 0.3272)<br>CNO effect F(1.000, 7.000)=9.101 p=0.0195 (ε correction: 1.000)<br>Interaction F(1.5658, 10.95)=3.844 p=0.0623 (ε correction: 0.3911)<br>Bonferroni's multiple comparisons test:<br>DREADD Gq Sal vs CNO: 1-24h p>0.9999
Suppl. Figure 2C	Two-way Mixed-effects model ANOVA (Geisser-Greenhouse's epsilon correction	D1 cre<br>Dreadd Gq = 8<br>2 outlier values removed<br>with Grubbs' method (Alpha = 0.01)	Fasting refeeding:<br>Food consumption (1h - 24h)<br>CNO: 2mg/kg	Time effect F(1.407, 9.851)=195.6 p=0.0001 (ε correction: 0.3518)<br>CNO effect F(1.000, 7.000)=16.21 p=0.0050 (ε correction: 1.000)<br>Interaction F(1.548, 10.06)=2.039 p=0.1836 (ε correction: 0.3870)
Suppl. Figure 2C	Two-way Mixed-effects model ANOVA (Geisser-Greenhouse's epsilon correction	D1 cre<br>Dreadd Gq = 9	Fasting refeeding:<br>Food consumption (1h - 24h)<br>CNO: 0.5mg/kg	Time effect F(1.938, 15.50)=606.0 p=0.0001 (ε correction: 0.4844)<br>CNO effect F(1.000, 8.000)=14.46 p=0.0052 (ε correction: 1.000)<br>Interaction F(1.642, 13.14)=8.142 p=0.1406 (ε correction: 0.4106)<br>Bonferroni's multiple comparisons test:<br>DREADD Gq Sal vs CNO: 1h p=0.0080; 2h p=0.0111; 4h p=0.0072
Suppl. Figure 2D	Two-way Mixed-effects model ANOVA (Geisser-Greenhouse's epsilon correction)	D2 cre mice<br>Dreadd Gi = 7	% Blood glucose change<br>vs baseline	Time effect F(2.576, 15.46)=104.2 p=0.0001 (ε correction: 0.3220)<br>CNO effect F(1.00, 6.00)=1.070 p=0.3409 (ε correction: 1.000)<br>Interaction F(3.077, 18.46)=0.4382 p=0.7330 (ε correction: 0.3846)
Suppl. Figure 2D	Two-way Mixed-effects model ANOVA (Geisser-Greenhouse's epsilon correction)	D2 cre mice<br>Dreadd Gq = 8	% Blood glucose change<br>vs baseline	Time effect F(1.698, 11.89)=55.82 p=0.0001 (ε correction: 0.2123)<br>CNO effect F(1.00, 7.00)=0.01822 p=0.8964 (ε correction: 1.000)<br>Interaction F(2.704, 18.93)=1.226 p=0.3380 (ε correction: 0.3380)
Suppl. Figure 2D	Two-way Mixed-effects model ANOVA (Geisser-	D1 cre mice<br>Dreadd Gi = 9	% Blood glucose change<br>vs baseline	Time effect F(1.901, 15.21)=74.77 p=0.0001 (ε correction: 0.2376)<br>CNO effect F(1.00, 8.00)=0.1887 p=0.6754

	Greenhouse's epsilon correction)			(ε correction: 1.000)<br>Interaction F (2.197, 17.58)=0.3944 p=0.6987<br>(ε correction: 0.2747)
Suppl. Figure 2D	Two-way Mixed-effects model ANOVA (Geisser-Greenhouse's epsilon correction)	D1 cre mice<br>Dreadd Gq = 9	% Blood glucose change<br>vs baseline	Time effect F(2.193, 17.54)=130.8 p=0.0001<br>(ε correction: 0.2741)<br>CNO effect F(1.00, 8.00) = 2.419 p=0.1585<br>(ε correction: 1.000)<br>Interaction F(3.290, 26.32) = 1.257 p=0.3601<br>(ε correction: 0.4113)
Figure Supp 2E	Mann Whitney test	D2cre mice (n=6), Neurons: Saline:42 / CNO:44	Firing rate (Hz)	U=712, p= 0.0612
Figure Supp 2E	Mann Whitney test	D2cre mice (n=6), Neurons: Saline:42 / CNO:44	Bursting rate (Hz)	U=579.5, p=0.0026
Figure Supp 2E	Mann Whitney test	D2cre mice (n=6), Neurons: Saline:42 / CNO:44	%SIB	U=627, p=0.0097
Figure Supp 2E	Mann Whitney test	D2cre mice (n=6), Neurons: Saline:42 / CNO:44	MSB	U=791, p=0.2369
Figure Supp 2E	Unpaired T-test	D1cre mice (n=4), Neurons: Saline:40 / CNO:39	Firing rate (Hz)	t(77)=3.888, p=0.0002
Figure Supp 2E	Mann Whitney test	D1cre mice (n=4), Neurons : Saline:40 / CNO:39	Bursting rate (Hz)	U=530.5, p=0.0133
Figure Supp 2E	Mann Whitney test	D1cre mice (n=4), Neurons : Saline:40 / CNO:39	%SIB	U=634.5, p= 0.1534
Figure Supp 2E	Unpaired T-test	D1cre mice (n=4), Neurons : Saline:40 / CNO:39	MSB	t(77)=1.953, p=0.0545
Supplementary Figure 3				
Suppl. Figure 3C	Two-way Mixed-effects model ANOVA (Satterthwaite correction for degrees of freedom)	D1GCamp = 5<br>D2GCamp = 5<br>12 trials per animals	Peri-event (Stop Run) (Zscore ; D1 vs D2)	Intercept effect F(1, 8.560)=5.056 p=0.0526<br>Genotype effect F(1, 8.560)=0.486 p=0.5044<br>Time effect F(1, 10.113)=2.3046 p=0.1596<br>Genotype x Time F(1, 10.11)=3.172 p=0.105<br>post hoc-permutation test between genotypes
Suppl. Figure 3D	Two-way Mixed-effects model ANOVA (Geisser-Greenhouse's epsilon correction	D2 cre mice<br>Dreadd Gi = 7	Chow consumption	Time effect F(1.782, 26.72)=15.45 p=0.0001<br>(ε correction: 0.4454)<br>CNO effect F(1.00, 60.00)=0.5417 p=0.4646<br>(ε correction: 1.000)<br>Interaction F(1.533, 22.99)=4.968 p=0.0227<br>(ε correction: 0.3632)<br>Bonferroni's multiple comparisons test:<br>DREADD Gi Sal vs CNO: 3h p=0.0006

Suppl. Figure 3D	Two-way Mixed-effects model ANOVA (Geisser-Greenhouse's epsilon correction	D2 cre mice<br>Dreadd Gq = 8	Chow consumption	Time effect F(1.922, 13.45)=3.763 p=0.0517 (ε correction: 0.4805)<br>CNO effect F(1.000, 7.000)=2.435 p=0.1626 (ε correction: 1.000)<br>Interaction F(1.647, 11.53)=1.052 p=0.3664 (ε correction: 0.4118)
Suppl. Figure 3E	Two-way Mixed-effects model ANOVA (Geisser-Greenhouse's epsilon correction	D2 cre mice<br>Dreadd Gi = 7	Wheel running activity	Time effect F(1.894, 11.36)=11.19 p=0.0023 (ε correction: 0.4735)<br>CNO effect F(1.000, 6.000)=17.85 p=0.0055 (ε correction: 1.000)<br>Interaction F(1.582, 9.490)=4.728 p=0.0438 (ε correction: 0.3954)<br>Bonferroni's multiple comparisons test:<br>DREADD Gi Sal vs CNO: 3h p=0.0871; 5h p=0.0231
Suppl. Figure 3E	Two-way Mixed-effects model ANOVA (Geisser-Greenhouse's epsilon correction	D2 cre mice<br>Dreadd Gq = 8<br>2 outlier values removed<br>with Grubbs' method (Alpha = 0.01)	Wheel running activity	Time effect F(2.084, 14.59)=35.25 p=0.0001 (ε correction: 0.5210)<br>CNO effect F(1.00 , 7.00)=14.38 p=0.0068 (ε correction: 1.000)<br>Interaction F(1.762, 11.45)=9.475 p=0.0045 (ε correction: 0.4404)<br>Bonferroni's multiple comparisons test:<br>DREADD Gq Sal vs CNO: 3h p=0.0011; 4h p=0.0726
Suppl. Figure 3F	Two-way Mixed-effects model ANOVA (Geisser-Greenhouse's epsilon correction	D1 cre<br>Dreadd Gi = 9	Chow consumption	Time effect F(1.856, 14.84)=10.76 p=0.0015 (ε correction: 0.4639)<br>CNO effect F(1.00, 8.00)=1.739 p=0.2238 (ε correction: 1.000)<br>Interaction F(2.506, 20.06)=3.931 p=0.0287 (ε correction: 0.6269)<br>Bonferroni's multiple comparisons test:<br>DREADD Gq Sal vs CNO: 1-24h p>0.9999
Suppl. Figure 3F	Two-way Mixed-effects model ANOVA (Geisser-Greenhouse's epsilon correction	D1 cre<br>Dreadd Gq = 9<br>2 outlier values removed<br>with Grubbs' method (Alpha = 0.01)	Chow consumption	Time effect F(2.569, 20.55)=21.55 p=0.0001 (ε correction: 0.6422)<br>CNO effect F(1.00, 8.00)=6.794 p=0.0313 (ε correction: 1.000)<br>Interaction F(2.150, 16.12)=6.184 p=0.0091 (ε correction: 0.5374)<br>Bonferroni's multiple comparisons test:<br>DREADD Gq Sal vs CNO: 3h p=0.0043
Suppl. Figure 3G	Two-way Mixed-effects model ANOVA (Geisser-Greenhouse's epsilon correction	D1 cre<br>Dreadd Gi = 9<br>2 outlier values removed<br>with Grubbs' method (Alpha = 0.01)	Wheel running activity	Time effect F(2.393, 19.15)=48.19 p=0.0001 (ε correction: 0.5983)<br>CNO effect F(1.000, 8.000)=2.106 p=0.1848 (ε correction: 1.000)<br>Interaction F(2.518, 18.89)=5.949 p=0.0067 (ε correction: 0.6295)<br>Bonferroni's multiple comparisons test:<br>DREADD Gi Sal vs CNO: 3h p=0.0328

Suppl. Figure 3G	Two-way Mixed-effects model ANOVA (Geisser-Greenhouse's epsilon correction	D1 cre<br>Dreadd Gq = 9	Wheel running activity	Time effect F(1.858, 14.86)=1.387 p=0.2787 (ε correction: 0.4644)<br>CNO effect F(1.000, 8.000)=12.14 p=0.0083 (ε correction: 1.000)<br>Interaction F(1.420, 11.36)=10.31 p=0.0048 (ε correction: 0.3551)<br>Bonferroni's multiple comparisons test:<br>DREADD Gq Sal vs CNO: 3h p=0.0097; 4h p=0.0536; 5h p=0.0450
Supplementary Figure 4				
Suppl. Figure 4A	Two-way Mixed-effects model ANOVA (Geisser-Greenhouse's epsilon correction)	D2 WT = 7<br>D2 GI = 7<br>D2 Gq = 8<br>(2 WT, 1 D1-Gi and 1 D1-Gq animals were exposed to a running wheel without recording)	Running wheel variation D1-33	Time effect F(7.220, 136.5)=3.247 p=0.0029 (ε correction: 0.2188)<br>Virus effect F(2, 19)=0.6835 p=0.5168<br>Interaction F(66, 624)=2.884 p=0.0001<br>Bonferroni's multiple comparisons test:<br>D2Gi vs D2WT: Day1 p=0.0278;
Suppl. Figure 4B	Two-way RM ANOVA (Geisser-Greenhouse's epsilon correction)	D2 WT = 9<br>D2 GI = 8<br>D2 Gq = 9	Total Mass	Time effect F(1.15, 26.42)=16.03 p=0.0003 (ε correction: 0.5744)<br>Virus effect F(2, 23)=0.8456 p=0.4422<br>Interaction F(4, 46)=1.247 p=0.3047
Suppl. Figure 4C	Two-way RM ANOVA (Geisser-Greenhouse's epsilon correction)	D2 WT = 9<br>D2 GI = 8<br>D2 Gq = 9	Fat Mass	Time effect F(1.216, 27.96)=40.10 p=0.0001 (ε correction: 0.5744)<br>Virus effect F(2, 23)=0.5713 p=0.5726<br>Interaction F(4, 46)=1.448 p=0.2336
Suppl. Figure 4D	Two-way RM ANOVA (Geisser-Greenhouse's epsilon correction)	D2 WT = 9<br>D2 GI = 8<br>D2 Gq = 9	Lean Mass	Time effect F(1.525, 35.08)=116.5 p=0.0001 (ε correction: 0.7625)<br>Virus effect F(2, 23)=0.8900 p=0.4243<br>Interaction F(4, 46)=2.186 p=0.0853
Suppl. Figure 4E	One-way ANOVA	D2 WT = 8<br>D2 GI = 8<br>D2 Gq = 9<br>1 outlier value removed<br>with Grubbs' method (Alpha = 0.01)	Lean mass variation (mean D1-D23)	F(2, 22)=7.281 p=0.0037<br>Bonferroni's multiple comparisons test:<br>D2 WT vs D2 Gi p=0.0342<br>D2 WT vs D2 Gq p=0.0039
Suppl. Figure 4E	One-way ANOVA	D2 WT = 9<br>D2 GI = 8<br>D2 Gq = 9	Lean mass variation (mean D23-D36)	F(2, 23)=3.737 p=0.0393<br>Bonferroni's multiple comparisons test:<br>D2 WT vs D2 Gq p=0.0825
Suppl. Figure 4F	Two-way RM ANOVA (Geisser-Greenhouse's epsilon correction)	D2 WT = 9<br>D2 GI = 8<br>D2 Gq = 9	Body Weight (g)	Time effect F(1.744, 40.12)=4.475 p=0.0001 (ε correction: 0.05286)<br>Virus effect F(2, 23)=0.3639 p=0.6989<br>Interaction F(66, 759)=1.206 p=0.1340
Suppl. Figure 4F	Two-way RM ANOVA (Geisser-Greenhouse's epsilon correction)	D2 WT = 9<br>D2 GI = 8<br>D2 Gq = 9	Body Weight variation (%)	Time effect F(1.743, 40.77)=4.313 p=0.0238 (ε correction: 0.05371)<br>Virus effect F(2, 23)=0.8304 p=0.4485<br>Interaction F(66, 759)=1.225 p=0.1225

Suppl. Figure 4G	Two-way Mixed-effects model ANOVA (Geisser-Greenhouse's epsilon correction)	D1 WT = 9<br>D1 GI = 11<br>D1 Gq = 11<br>(3 WT and 1 D1-Gq animals were exposed to a running wheel without recording)	Running wheel variation D1-33	Time effect F(8.351, 228.5)=5.553 p=0.0001 (ε correction: 0.2531)<br>Virus effect F(2, 28)=0.0087 p=0.0087<br>Interaction F 66, 903)=2.061 p=0.0001<br>Bonferroni's multiple comparisons test: D1Gq vs D1WT: Day2 p=0.0042; Day3 p=0.0045; Day4 p=0.0006; Day5 p=0.0151; Day6 p=0.0054; Day8 p=0.0197; Day9 p=0.0132; Day10 p=0.0059; Day11 p=0.0273; Day12 p=0.0052;
Suppl. Figure 4H	Two-way RM ANOVA (Geisser-Greenhouse's epsilon correction)	D1 WT = 12<br>D1 GI = 11<br>D1 Gq = 12	Total Mass	Virus effect F(2, 32)=1.123 p=0.3377<br>Time effect F(1.534, 49.10)=52.54 p=0.0001 (ε correction: 0.7672)<br>Interaction F 4, 64)=1.275 p=0.2891
Suppl. Figure 4I	Two-way RM ANOVA (Geisser-Greenhouse's epsilon correction)	D1 WT = 12<br>D1 GI = 11<br>D1 Gq = 12	Fat Mass	Virus effect F 2, 32)=2.059 p=0.1441<br>Time effect F(1.366, 43.71)=223.4 p=0.0001 (ε correction: 0.6830)<br>Interaction F(4, 64)=3.723 p=0.0087<br>Bonferroni's multiple comparisons test: D1Gq vs D1WT: D23 p=0.0345;
Suppl. Figure 4J	Two-way RM ANOVA (Geisser-Greenhouse's epsilon correction)	D1 WT = 12<br>D1 GI = 11<br>D1 Gq = 12	Lean Mass	Virus effect F(2, 32) = 2.498 p=0.0982<br>Time effect F(1.262, 40.40)=122.9 p=0.0001 (ε correction: 0.6312)<br>Interaction F(4, 64)=1.547 p=0.1994
Suppl. Figure 4K	One-way ANOVA	D1 WT = 12<br>D1 GI = 11<br>D1 Gq = 12	Lean mass variation (mean D1-D23)	F(2, 32)=2.193 p=0.1280
Suppl. Figure 4K	One-way ANOVA	D1 WT = 12<br>D1 GI = 11<br>D1 Gq = 12	Lean mass variation (mean D23-D36)	F(2, 32)=0.7046 p=0.5018
Suppl. Figure 4L	Two-way RM ANOVA (Geisser-Greenhouse's epsilon correction)	D1 WT = 12<br>D1 GI = 11<br>D1 Gq = 12	Body Weight (g)	Virus effect F(2, 32)=1.231 p=0.3053<br>Time effect F(2.025, 64.80)=25.71 p=0.0001 (ε correction: 0.06328)<br>Interaction F(64, 1024)=1.559 p=0.0040<br>Bonferroni's multiple comparisons test: D1Gq vs D1WT: D1 p=0.0465;
Suppl. Figure 4L	Two-way RM ANOVA (Geisser-Greenhouse's epsilon correction)	D1 WT = 12<br>D1 GI = 11<br>D1 Gq = 12	Body Weight variation (%)	Virus effect F(2, 32)=3.227 p=0.0529<br>Time effect F(1.956, 66.60)=25.98 p=0.0001 (ε correction: 0.06113)<br>Interaction F(64, 1024)=1.497 p=0.0082<br>Bonferroni's multiple comparisons test: D1Gq vs D1WT: D1 p=0.0003;
Supplementary Figure 5				
Suppl. Figure 5A	Two-way Mixed-effects model ANOVA (Geisser-	D1-D2 WT = 9<br>D1Gq-D2Gi = 8<br>D1Gi-D2Gq = 9	Running wheel variation D1-33	Time effect F(5.387, 120.0)=3.491 p=0.0045 (ε correction: 0.1633)<br>Virus effect F(2, 23)=7.604 p=0.0029

	Greenhouse's epsilon correction)	(2 WT and 2 D1Gq-D2Gi animals were exposed to a running wheel without recording)		Interaction F(66, 735)=3.960 p=0.0001<br>Bonferroni's multiple comparisons test:<br>D1-D2 WT vs. D1Gq-D2Gi: Day1 p=0.00152; Day3 p=0.0096; Day4 p=0.0410; Day5 p=0.0052; Day6 p=0.0111; Day7 p=0.0121; Day8 p=0.0113; Day9 p=0.0047; Day10 p=0.0379; Day11 p=0.0120; Day13 p=0.0140; Day14 p=0.0221; Day16 p=0.0055; Day17 p=0.0028; Day18 p=0.0002; Day20 p=0.0413; Day21 p=0.0149; Day22 p=0.0380
Suppl. Figure 5B	Two-way RM ANOVA (Geisser-Greenhouse's epsilon correction)	D1-D2 WT = 11<br>D1Gq-D2Gi = 8<br>D1Gi-D2Gq = 11	Total Mass	Virus effect F(2, 27)=5.959 p=0.0072<br>Time effect F(1.313, 35.45)=14.92 p=0.0002 (ε correction: 0.6564)<br>Interaction F(4, 54)=5.141 p=0.0014<br>Bonferroni's multiple comparisons test:<br>D1-D2 WT vs. D1Gq-D2Gi: D0 p=0.0010; D23 p=0.0023<br>D34 p=0.0176
Suppl. Figure 5C	Two-way RM ANOVA (Geisser-Greenhouse's epsilon correction)	D1-D2 WT = 11<br>D1Gq-D2Gi = 8<br>D1Gi-D2Gq = 11	Fat Mass	Virus effect F(2, 27)=6.471 p=0.0051<br>Time effect F(1.279, 33.88)=18.28 p=0.0001 (ε correction: 0.6393)<br>Interaction F(4, 53)=4.210 p=0.0049<br>Bonferroni's multiple comparisons test:<br>D1-D2 WT vs. D1Gq-D2Gi: D0 p=0.0022; D23 p=0.0040<br>D34 p=0.0096<br>D1-D2 WT vs. D1Gi-D2Gq: D0 p=0.0038;
Suppl. Figure 5D	Two-way RM ANOVA (Geisser-Greenhouse's epsilon correction)	D1-D2 WT = 11<br>D1Gq-D2Gi = 8<br>D1Gi-D2Gq = 11	Lean Mass	Virus effect F(2, 27)=2.368 p=0.1129<br>Time effect F(1.260, 33.39)=32.66 p=0.0001 (ε correction: 0.6301)<br>Interaction F(4, 53)=4.563 p=0.0031<br>Bonferroni's multiple comparisons test:<br>D1-D2 WT vs. D1Gq-D2Gi: D23 p=0.0308; D34 p=0.0437
Suppl. Figure 5E	One-way ANOVA	D1-D2WT= 10<br>D1Gi-D2Gq = 8<br>D1Gq-D2Gi = 11<br>1 outlier values removed<br>with Grubbs' method (Alpha = 0.01)	Lean mass variation (mean D1-D23)	F(2, 32)=6.872 p=0.0039<br>Bonferroni's multiple comparisons test:<br>D1-D2 WT vs D1Gi-D2Gq p=0.0948<br>D1Gq-D2Gi vs D1Gi-D2Gq p=0.0034
Suppl. Figure5 E	One-way ANOVA	D1-D2WT= 10<br>D1Gi-D2Gq = 8<br>D1Gq-D2Gi = 10<br>2 outlier values removed<br>with Grubbs' method (Alpha = 0.01)	Lean mass variation (mean D23-D36)	F(2, 25)=15.07 p=0.0001<br>Bonferroni's multiple comparisons test:<br>D1-D2 WT vs D1Gi-D2Gq p=0.0104

Suppl. Figure5 F	Two-way RM ANOVA (Geisser-Greenhouse's epsilon correction)	D1 WT = 12<br>D1 GI = 11<br>D1 Gq = 12	Body Weight (g)	Virus effect F(2, 27)=7.241 p=0.0030<br>Time effect F(1.901, 51.53)=12.34 p=0.0001 (ε correction: 0.6301)<br>Interaction F(66, 891)=3.816 p=0.0001<br>Bonferroni's multiple comparisons test:<br>D1Gq-D2Gi vs D1-D2 WT D1-27 p=0.01<br>D1Gq-D2Gi vs D1-D2 WT D28-33 p=0.05
Suppl. Figure5 F	Two-way RM ANOVA (Geisser-Greenhouse's epsilon correction)	D1 WT = 12<br>D1 GI = 11<br>D1 Gq = 12	Body Weight variation (%)	Virus effect F(2, 27)=8.788 p=0.0011<br>Time effect F(1.985, 53.61)=12.24 p=0.0001 (ε correction: 0.06016)<br>Interaction F(66, 891)=4.310 p=0.0001<br>Bonferroni's multiple comparisons test:<br>D1Gq-D2Gi vs D1-D2 WT D1 p=0.0131, D2 p=0.0014, D3 p=0.0074 D4 p=0.0172 D5 p=0.0125, D6 p=0.0190, D7 p=0.0219, D8 p=0.046, D9 p=0.0240, D10 p=0.0135, D11 p=0.0238, D12 p=0.0311, D13 p=0.0071, D14 p=0.0391, D15 p=0.0284 , D16 p=0.0358, D17 p=0.0195, D18 p=0.0192, D19 p=0.0201, D20 p=0.0340, D21 p=0.0299<br>D1GI-D2GQ vs D1-D2 WT D2 p=0.0095, D3 p=0.0041 D4 p=0.0220 D5 p=0.0103
Supplementary Figure 6				
Suppl. Figure 6A	Two-way RM ANOVA	D1-D2WT= 8<br>D1Gq-D2Gi = 7<br>D1Gi-D2Gq = 7	Cumulative Chow consumption 2h under leptin or saline	Group effect F(2, 19)=92.61 p=0.0001<br>Leptin effect F(1, 19)=14.83 p=0.0011<br>Interaction F(2, 19)=0.8090 p=0.4601
Suppl. Figure 6A	Two-way RM ANOVA	D1-D2WT= 8<br>D1Gq-D2Gi = 7<br>D1Gi-D2Gq = 7	Cumulative Chow consumption 24h under leptin or saline	Group effect F(2, 19)=19.20 p=0.0543<br>Leptin effect F(1, 19)=54.72 p=0.0001<br>Interaction F(2, 19)=3.410 p=0.0001
Suppl. Figure 6B	Two-way Mixed-effects model ANOVA	D1-D2WT= 8<br>D1Gq-D2Gi = 7<br>D1Gi-D2Gq = 7<br>1 outlier value removed<br>with Grubbs' method (Alpha = 0.01)	Cumulative Chow consumption 2h under ghrelin or saline	Group effect F(1, 18)=30.26 p=0.0001<br>Ghrelin effect F(2, 19)=80.60 p=0.0001<br>Interaction F(2, 18)=16.60 p=0.0001<br>Bonferroni's multiple comparisons test:<br>Saline:<br>D1-D2 WT vs D1Gq-D2Gi p=0.0032<br>D1-D2 WT vs D1Gi-D2Gq p>0.9999<br>D1Gq-D2Gi vs D1Gi-D2Gq p=0.0547<br>Ghrelin:<br>D1-D2 WT vs D1Gq-D2Gi p=0.0001<br>D1-D2 WT vs D1Gi-D2Gq p=0.0025<br>D1Gq-D2Gi vs D1Gi-D2Gq p=0.0001
Suppl. Figure 6B	Two-way RM ANOVA	D1-D2WT= 8<br>D1Gq-D2Gi = 7<br>D1Gi-D2Gq = 7	Cumulative Chow consumption 24h under ghrelin or saline	Group effect F(2, 19)=9.446 p=0.0014<br>Ghrelin effect F(1, 19)=6.859 p=0.0169<br>Interaction F(2, 19)= 2.327 p=0.1247

Suppl. Figure 6C	Two-way Mixed-effects model ANOVA (Geisser-Greenhouse's epsilon correction	D1-D2WT= 8<br>D1Gq-D2Gi = 8<br>D1Gi-D2Gq = 9	Total Wheel running (m) activity during ABA protocol	Time effect F(3.201, 67.22)=10.35 p=0.0001<br>(ε correction: 0.6402)<br>group effect F(2, 22)=7.092 p=0.0042<br>Interaction F(10, 105)=7.672 p=0.0001<br>Bonferroni's multiple comparisons test:<br>D1-D2WT vs D1Gq-D2Gi: D1 night p=0.0196<br>D1-D2WT vs D1GI-D2Gq: D2 night p=0,0600; D3 night p=0,0900
Suppl. Figure 6D	Two-way Mixed-effects model ANOVA (Geisser-Greenhouse's epsilon correction	D1-D2WT= 8<br>D1Gq-D2Gi = 8<br>D1Gi-D2Gq = 9	Wheel running variation during ABA protocol	Time effect F(2.856, 59.27)=13.67 p=0.0001<br>(ε correction: 0.7140)<br>group effect F(2, 22)=13.22 p=0.0002<br>Interaction F(8, 83)=1.159 p=0.3336

Images of viral expression

D1 cre mice (Fig 1G-K ; Gi=9, Gq=8)

27209
D1gi

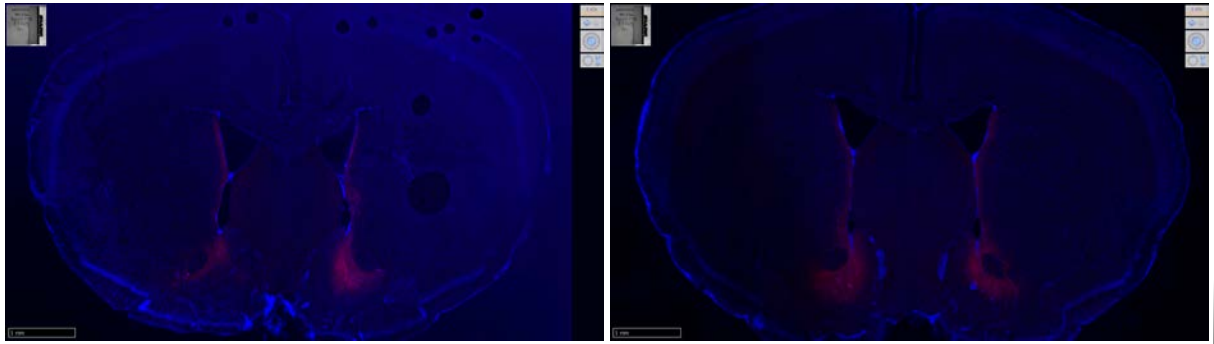

11048
D1gq

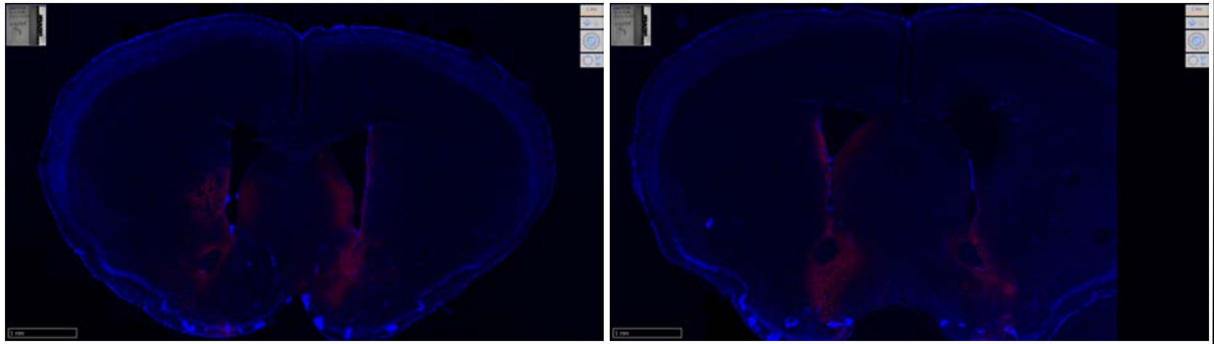

97517
D1gi

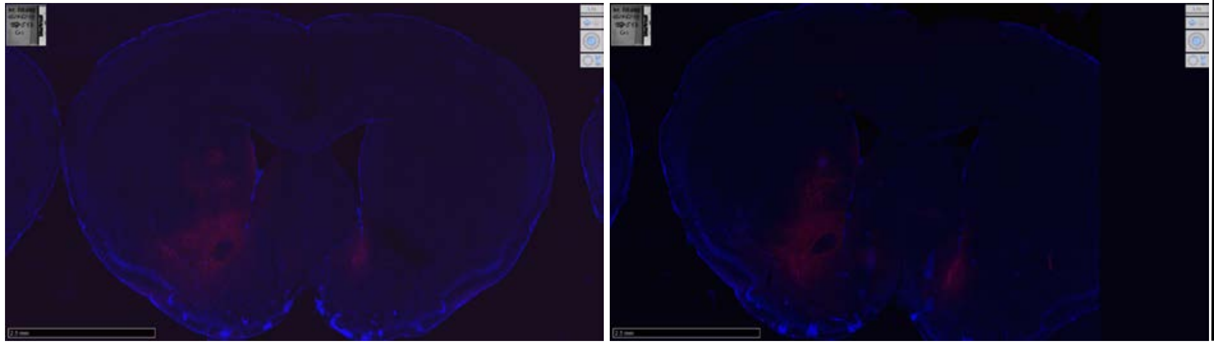

27534
D1gq

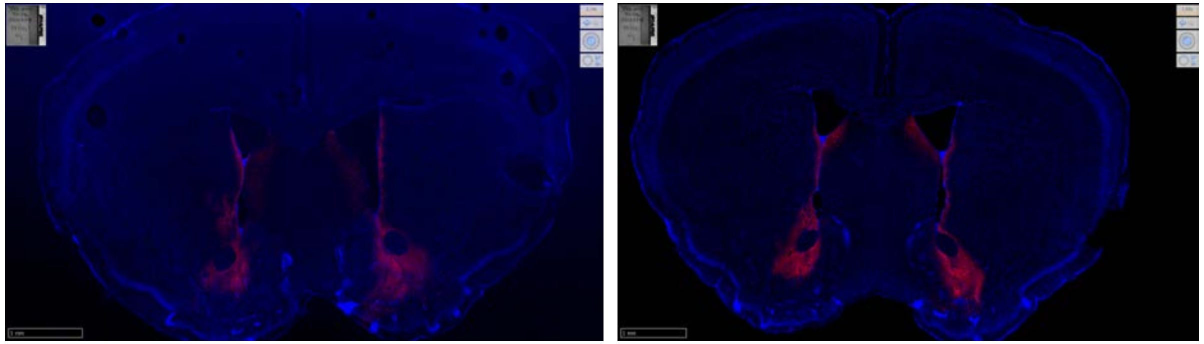

27348
D1gq

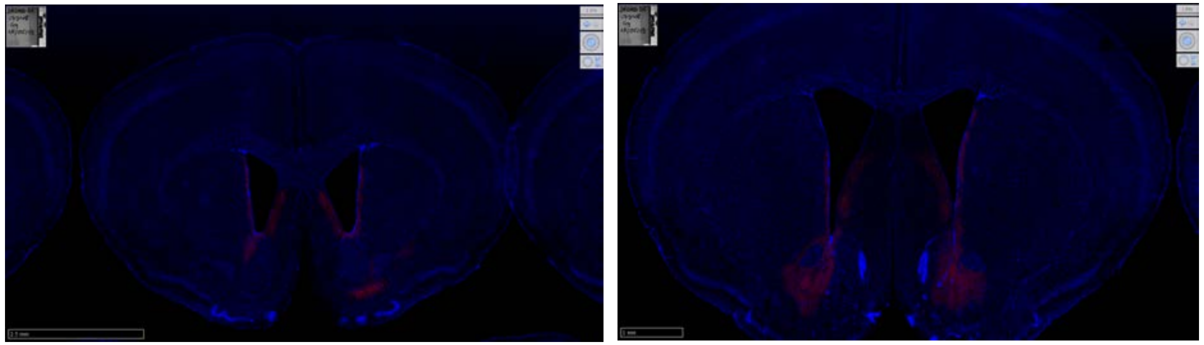

27420
D1gq

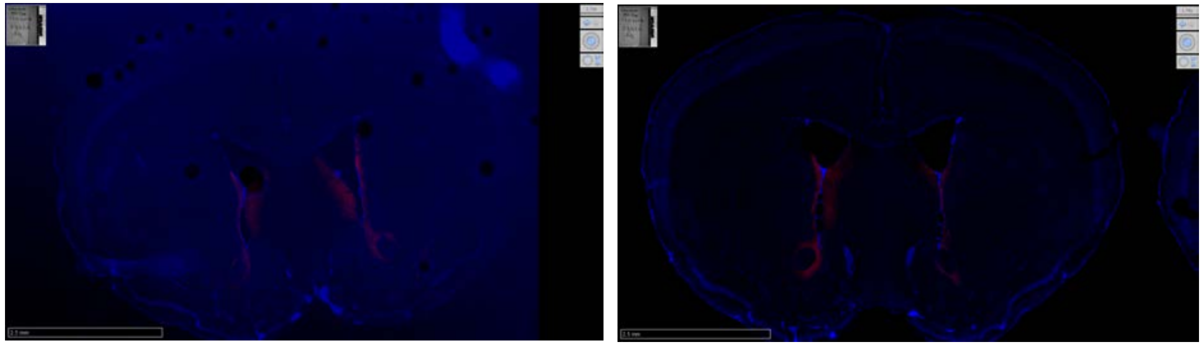

10658
D1gq

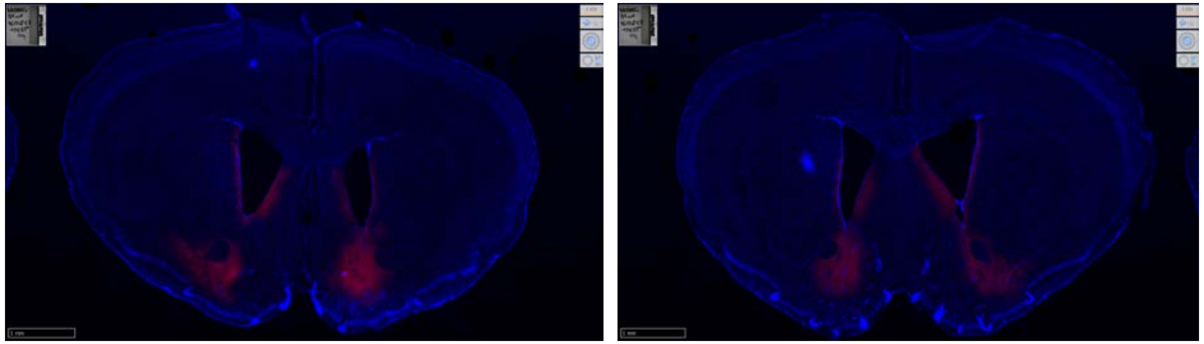

28711
D1gi

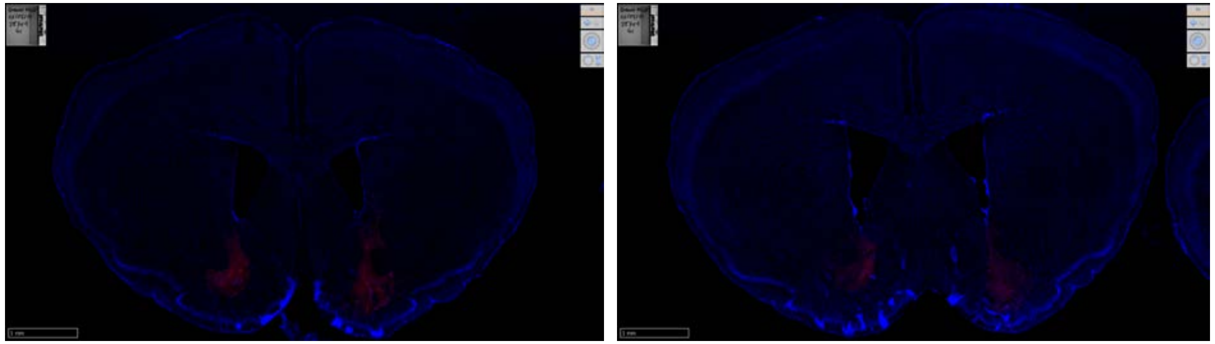

37101
D1gq

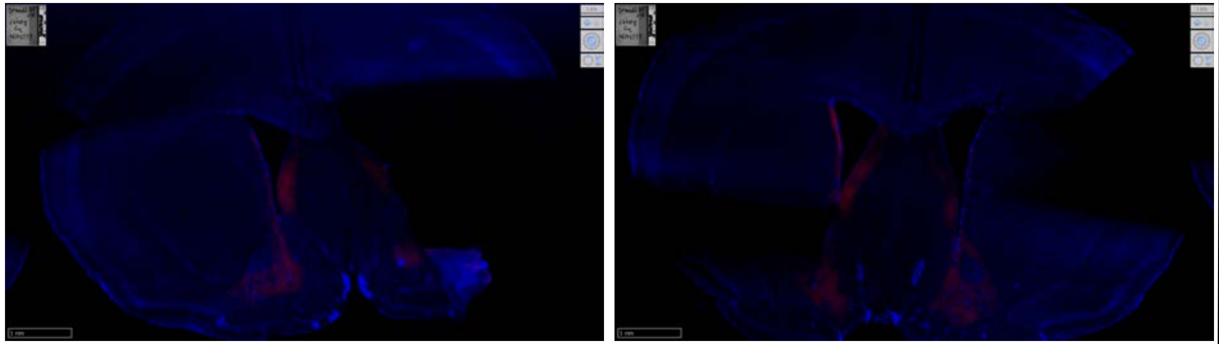

67191
D1gi

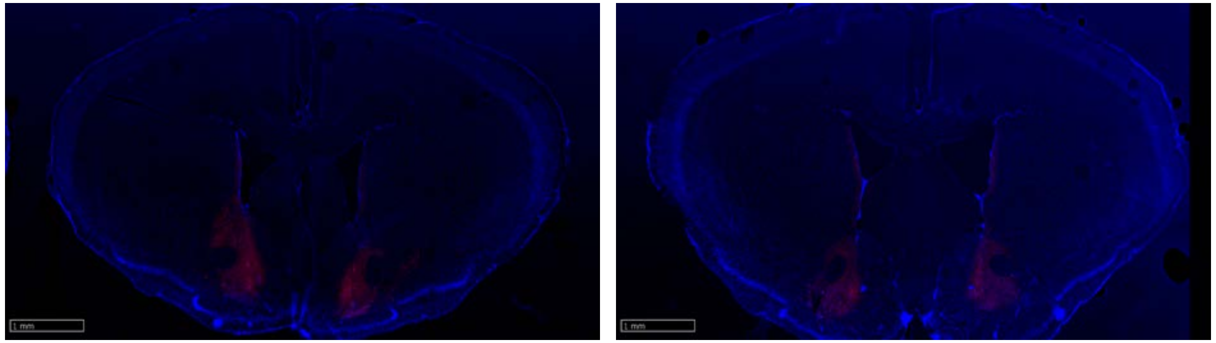

69846
D1gi

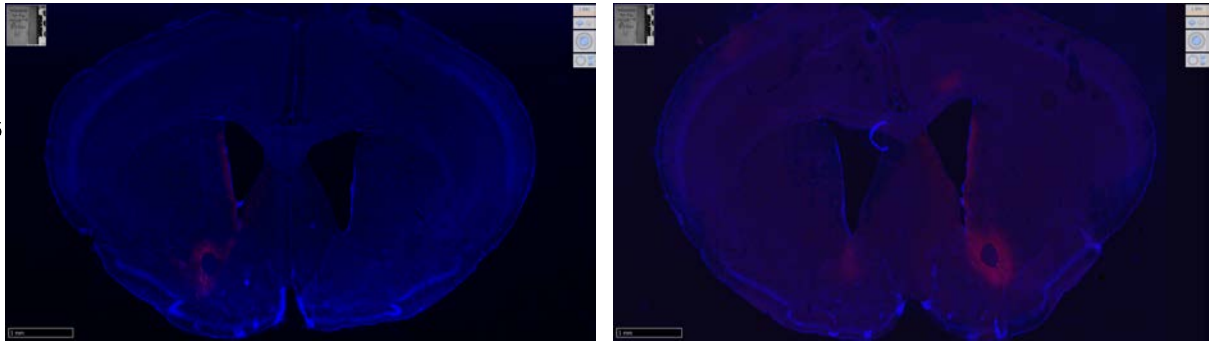

89116
D1gi

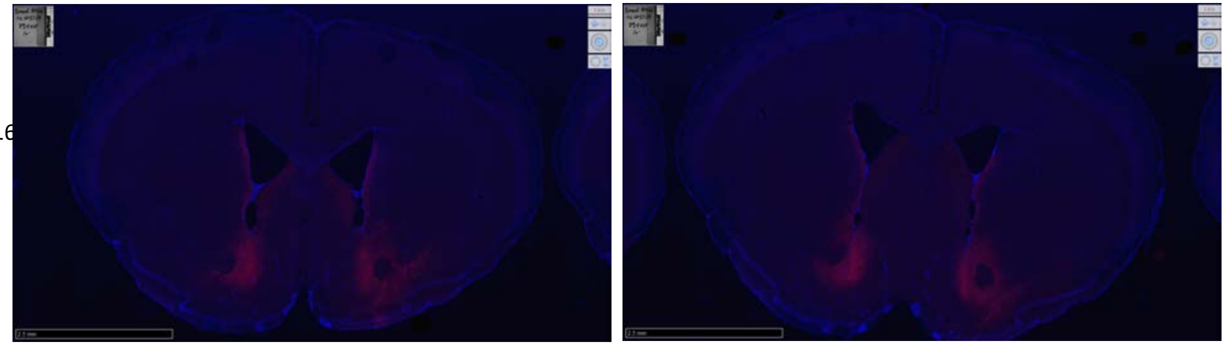

97141
D1gi

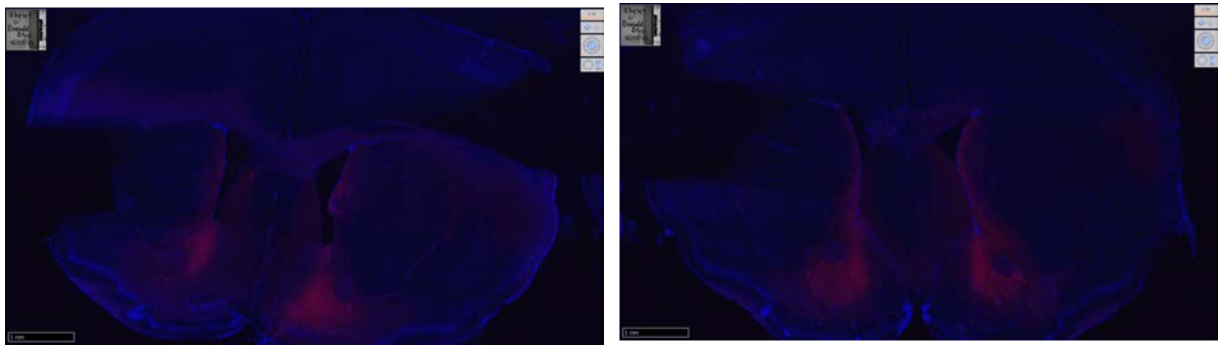

97236
D1gq

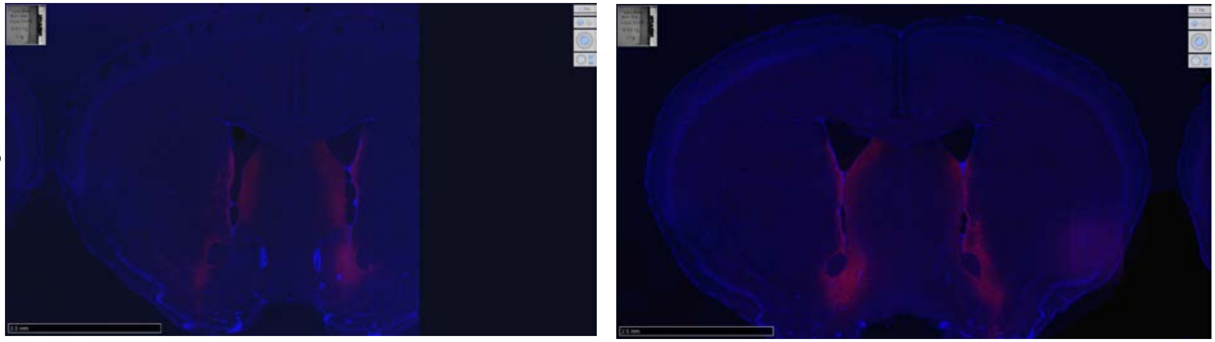

97358
D1gi

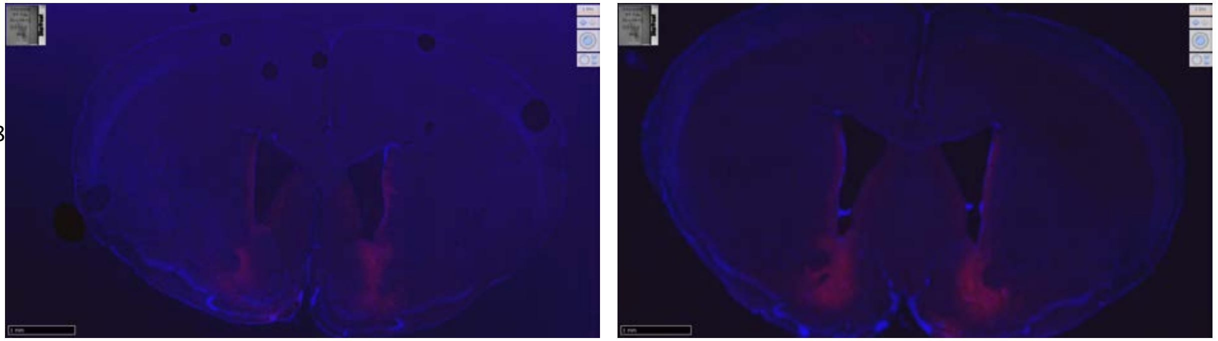

97485
D1gq

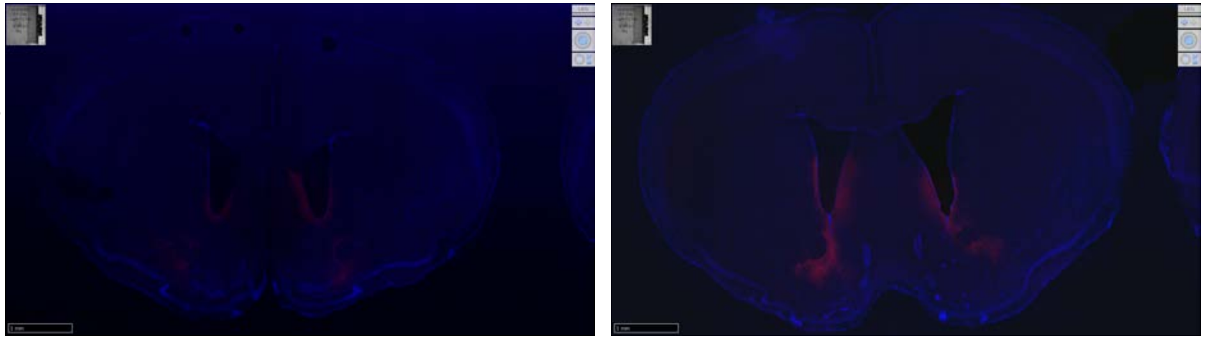

27302
D1gi

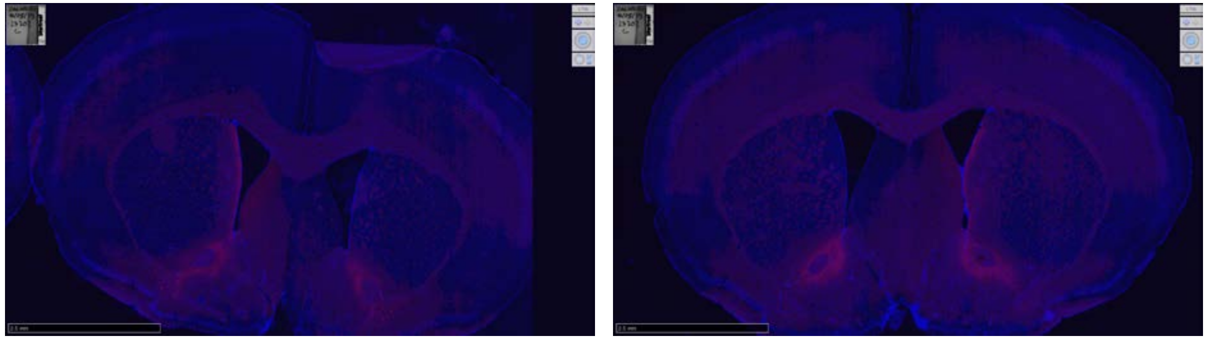

D2 cre mice (Fig 1B-F ; Gi=9, Gq=7)

10775
D2gi

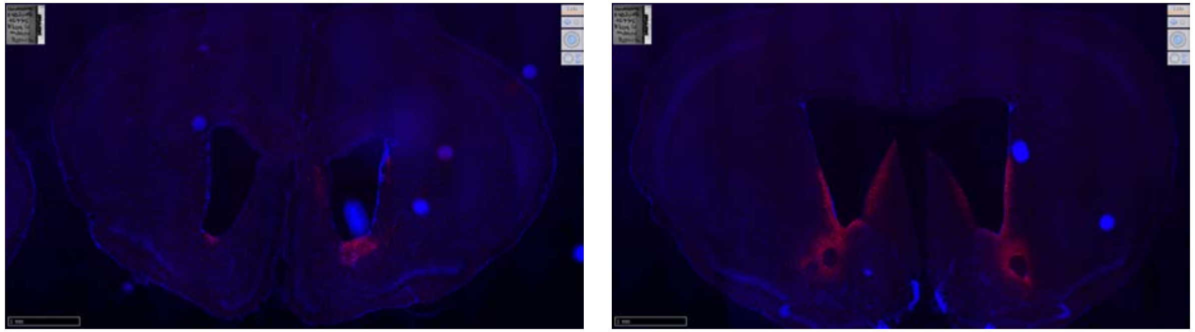

10937
D2gi

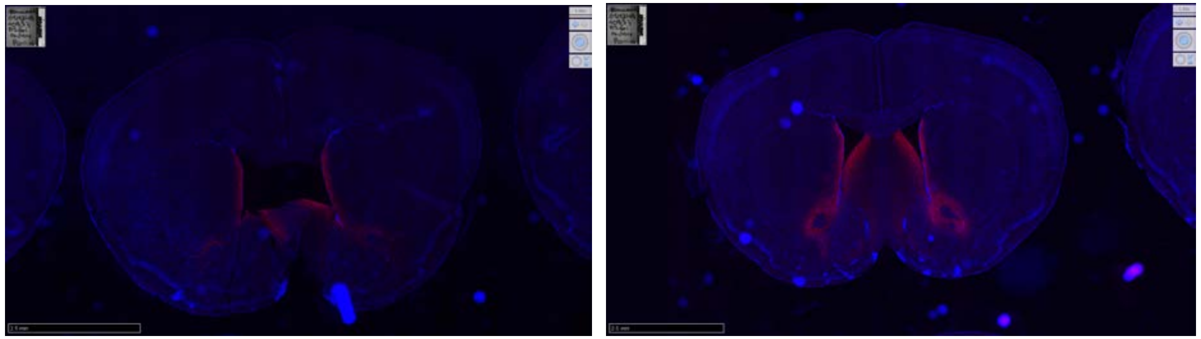

27460
D2gi

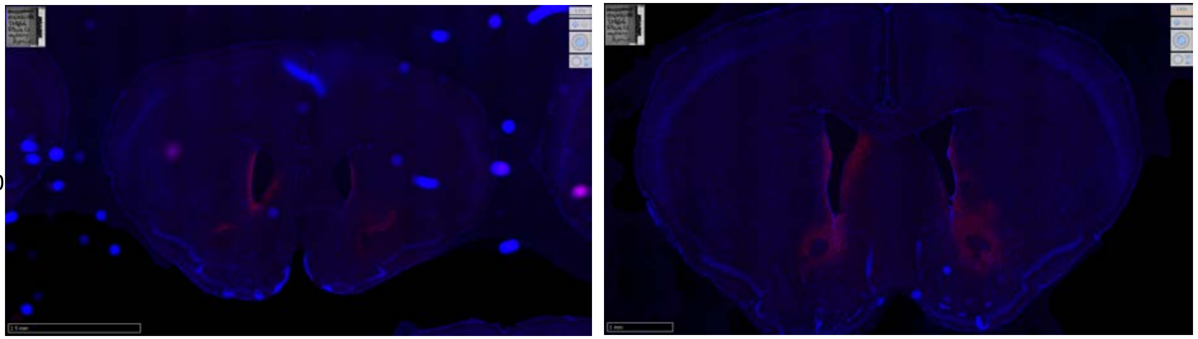

28510
D2gq

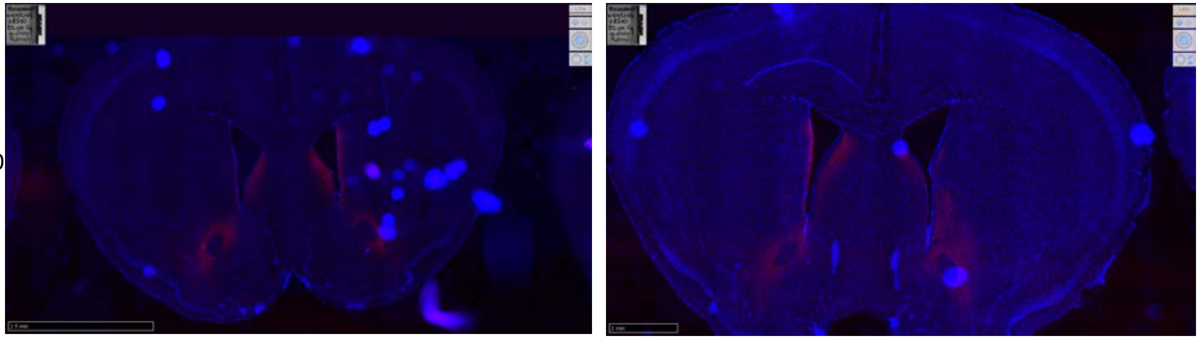

28888
D2gq

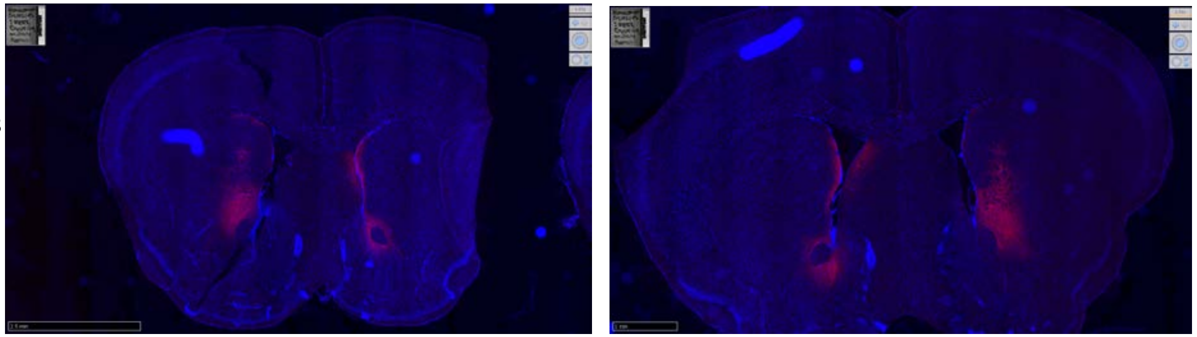

48991
D2gi

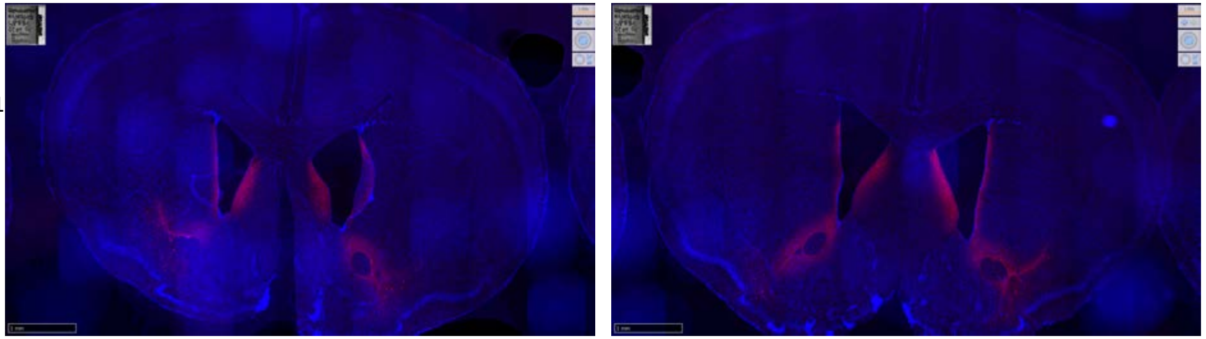

49019
D2gq

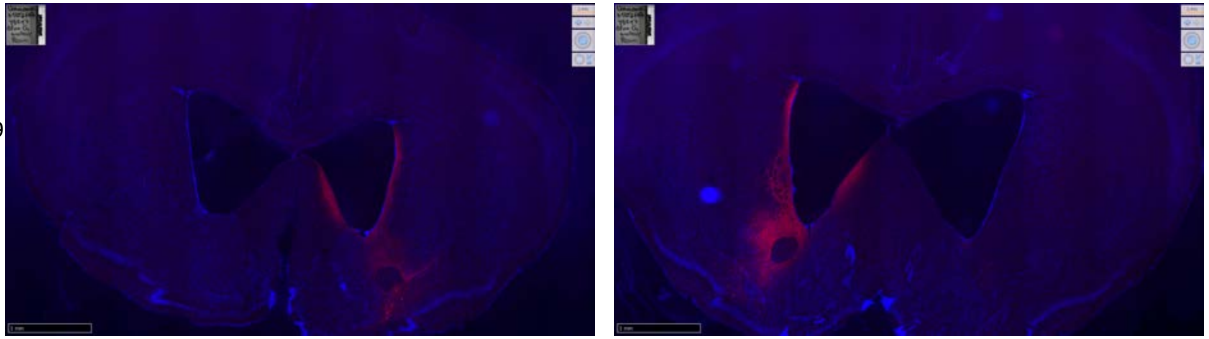

49117
D2gi

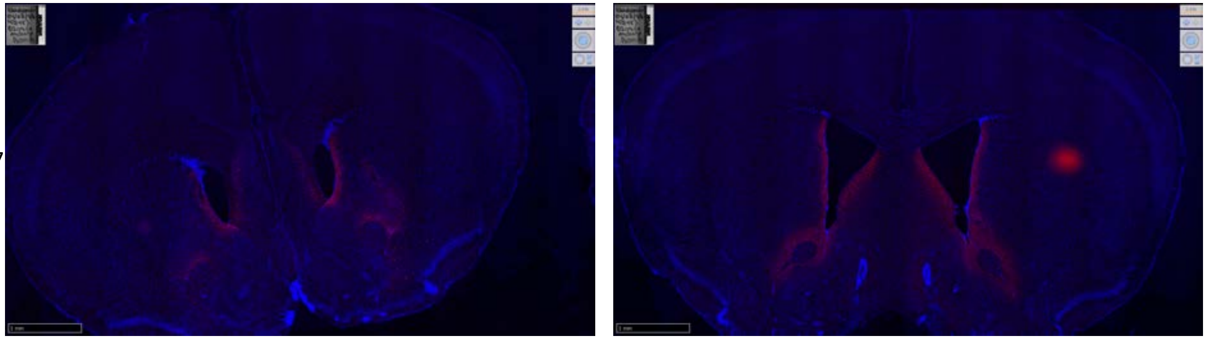

88698
D2gi

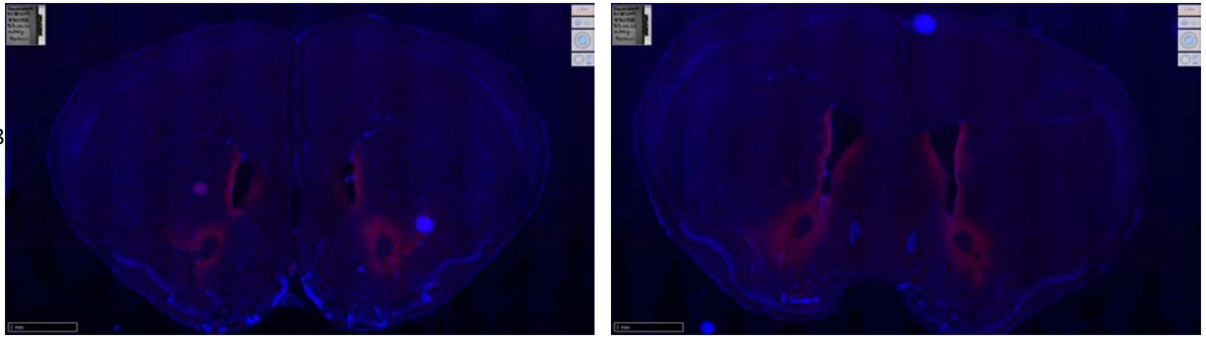

97163
D2gi

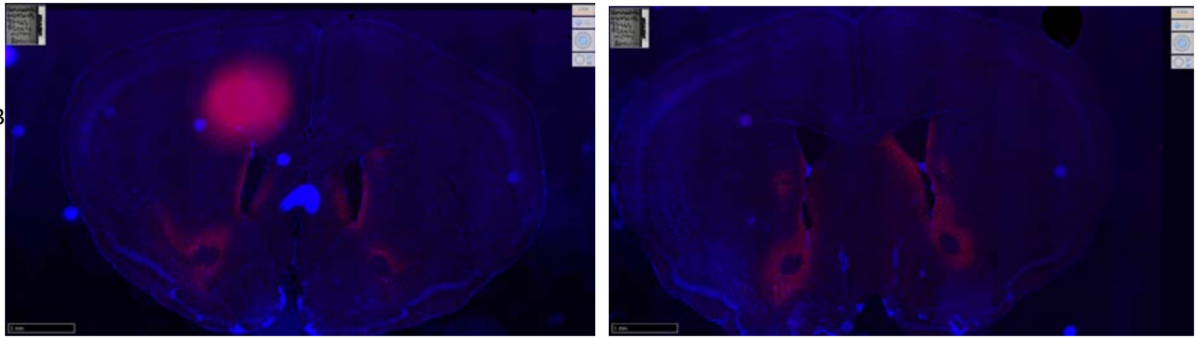

97317
D2gq

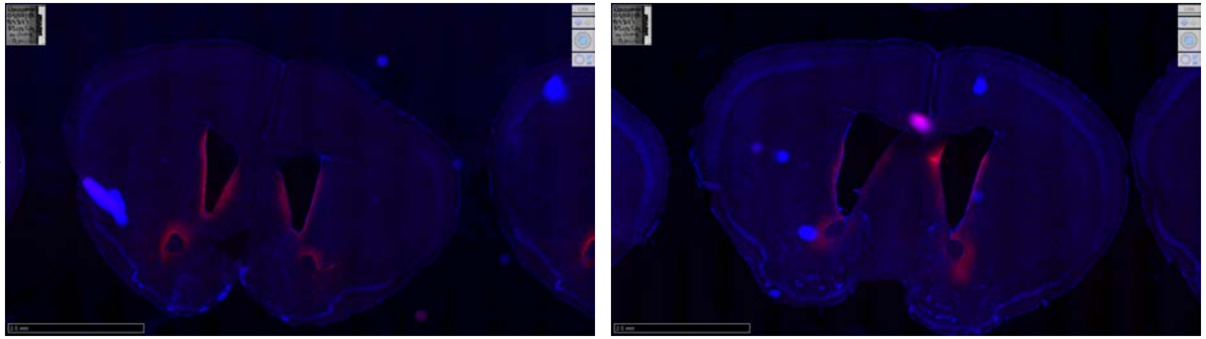

2 D2gi and 3 D2gq were used
for electrophysiology ex vivo
(Viral expression confirmed but not recorded)

D1 cre mice (Fig 2B Gi=6 , Gq=9)

Gq cohort include 4 animals implanted for fiberphotometry(Fig 2E-F)

79342
D1gi

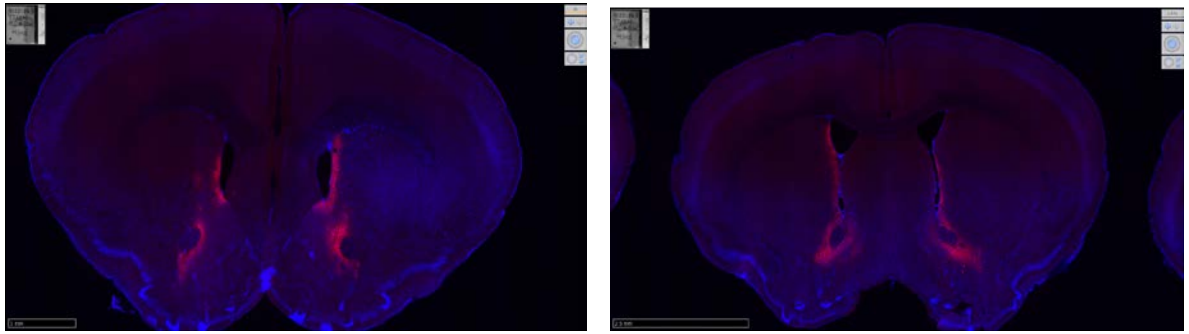

79480
D1gi

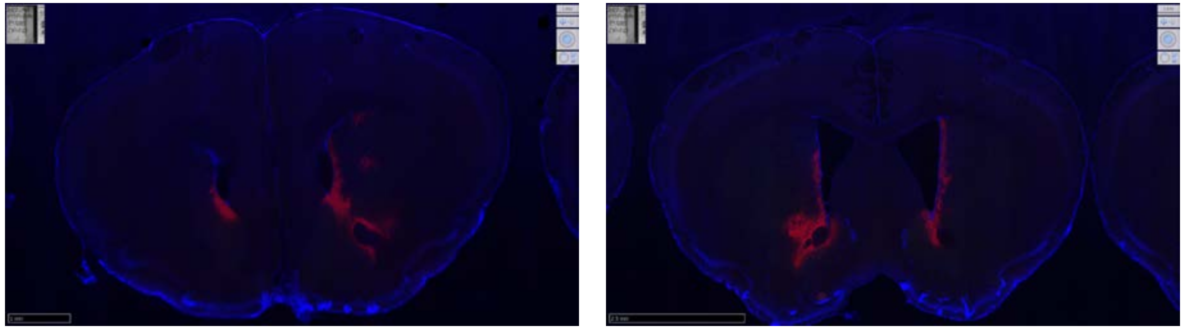

83272
D1gi

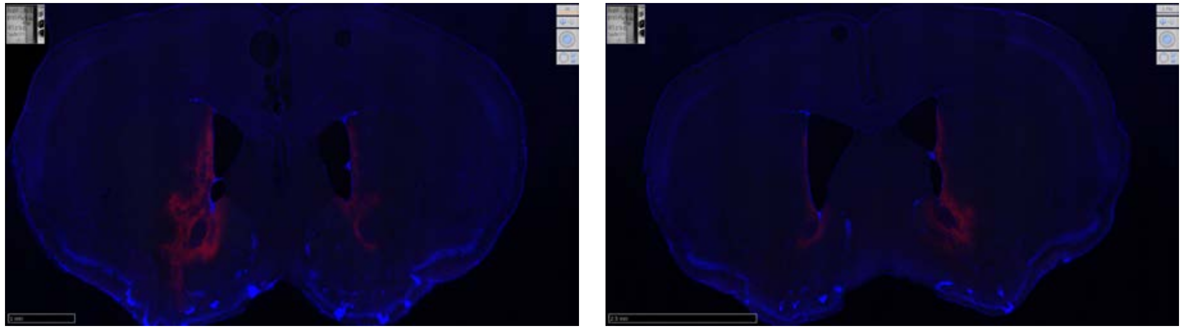

79327
D1gi

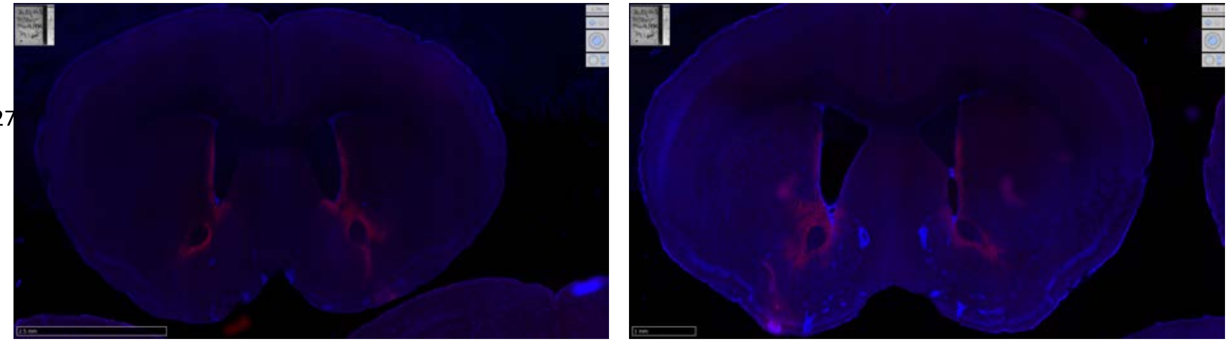

83733
D1gi

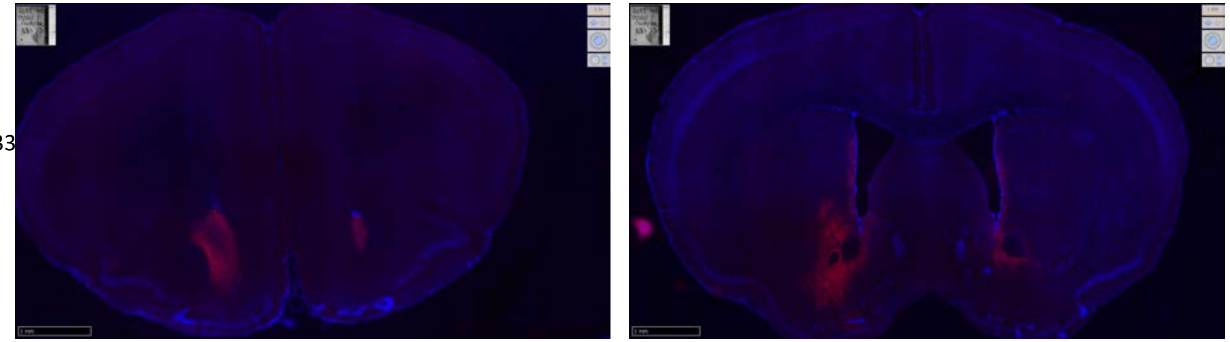

79130
D1gq

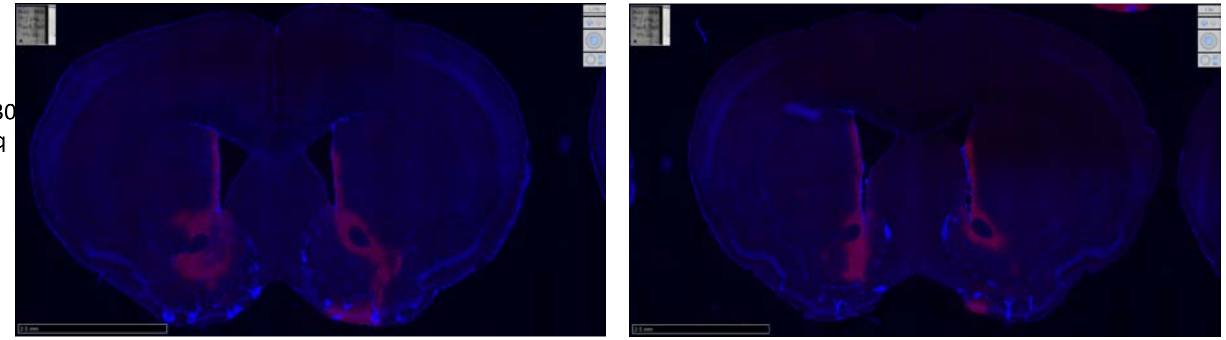

79003
D1gi

Missing image

83339
D1gq

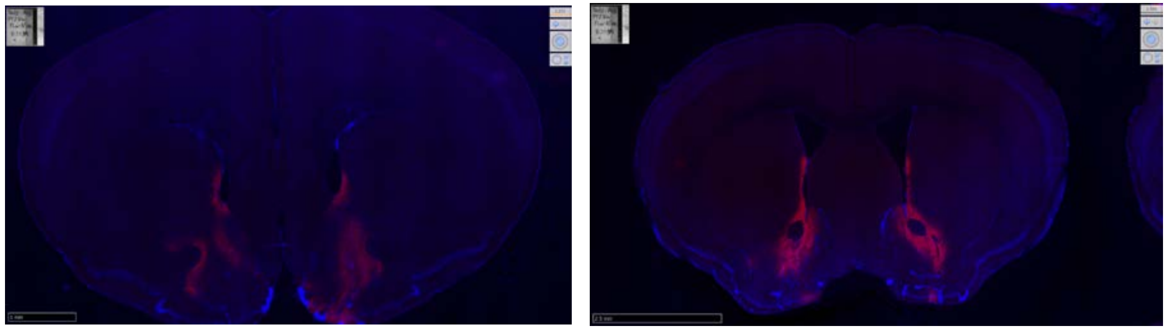

58734
D1gq

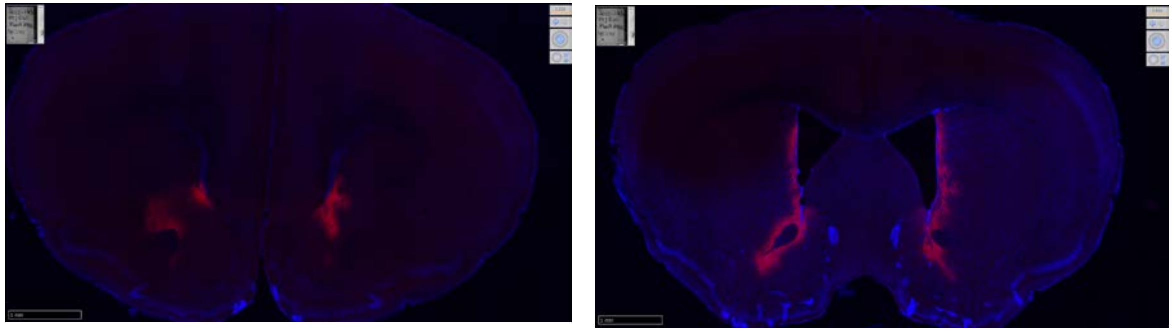

83748
D1gq

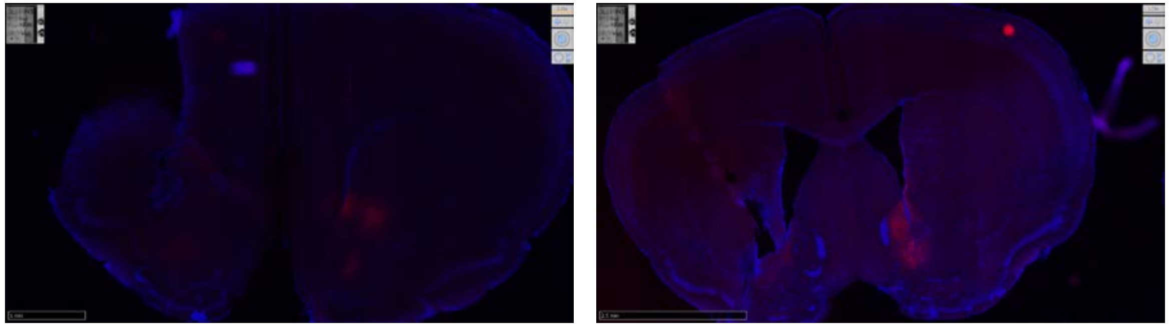

79113
D1gq

Missing
image

Used for pavlovian milk consumption (Fig 2B) and fiberphotometry (Fig 2E-F)

79041
D1gq
dlight

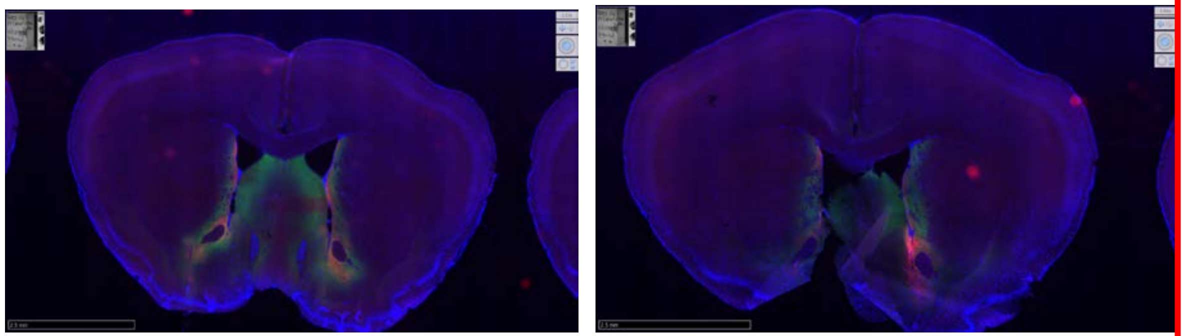

58695
D1gq
dlight

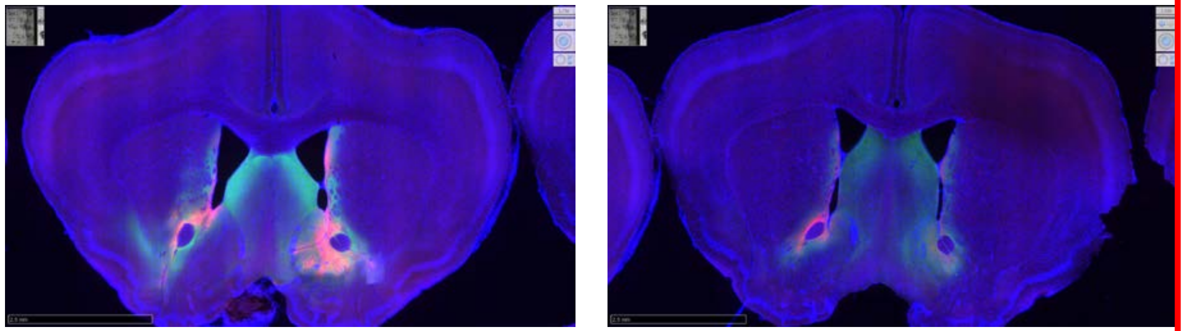

67134
D1gq
dlight

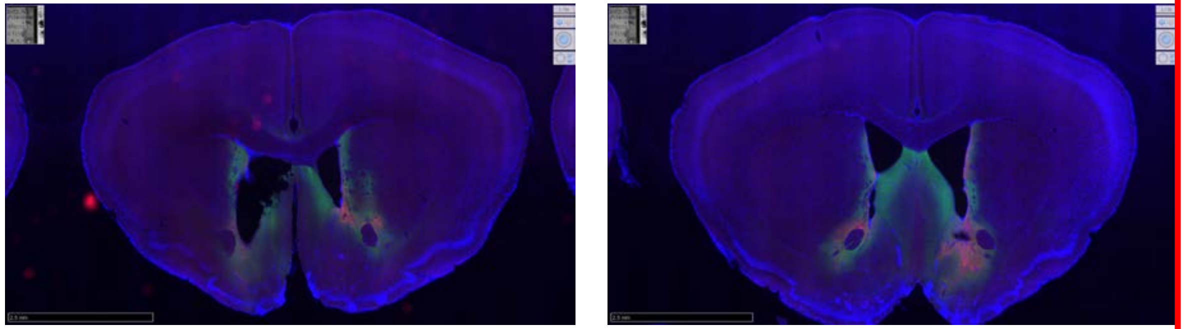

64219
D1gq
dlight

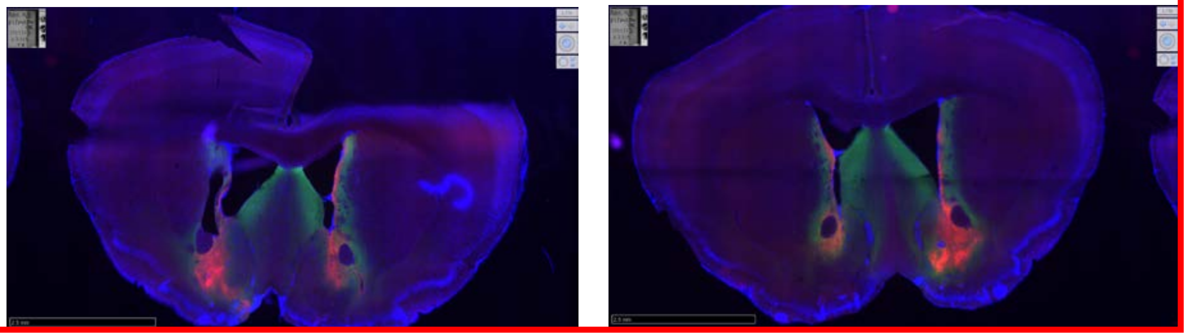

D2 cre mice (Fig 2B Gi=7, Gq=8)

Gq cohort include 3 animals implanted for fiberphotometry (Fig 2G-H)

78975
D2gi

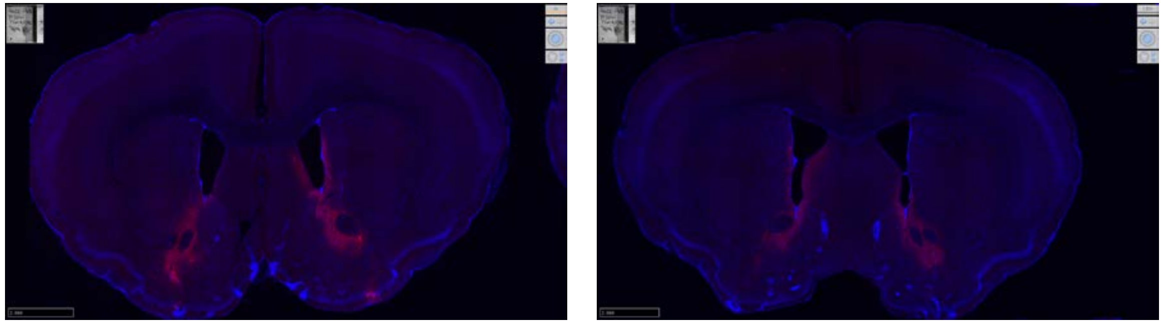

52515
D2gi

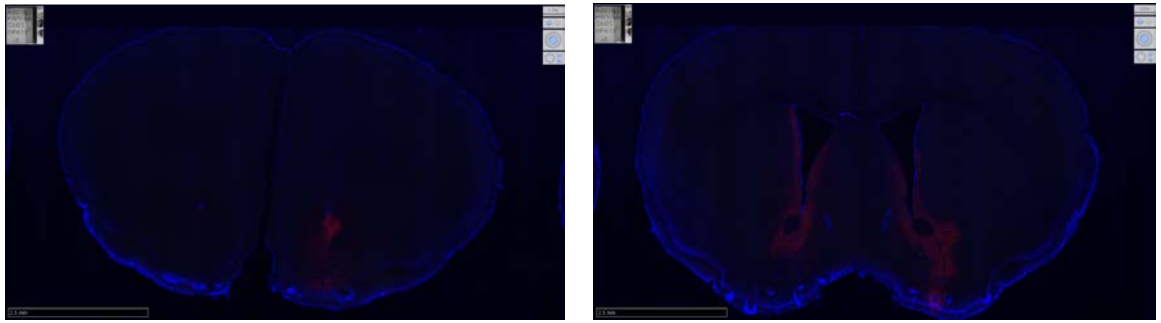

83546
D1gi

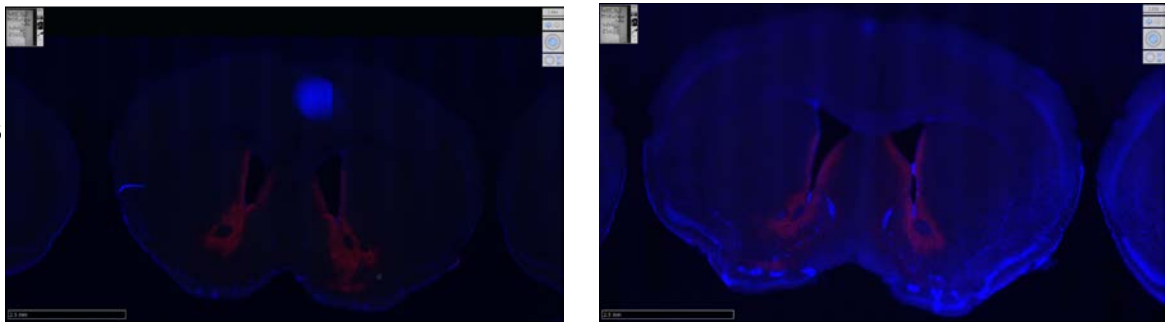

27079
D2gi

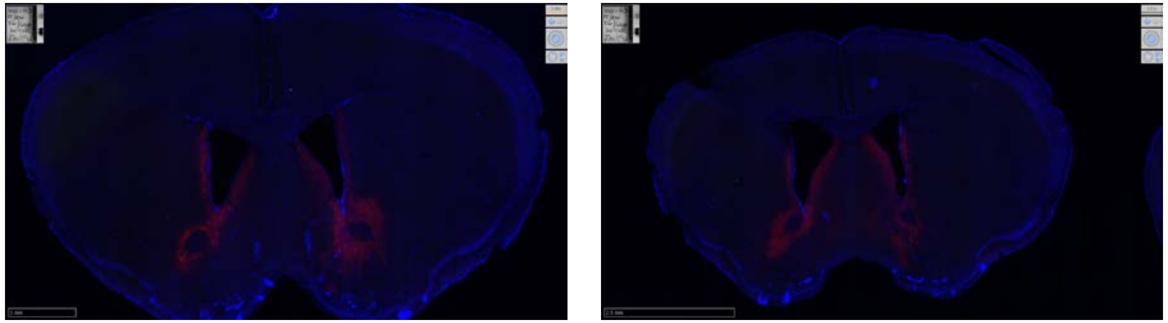

83629
D2gi

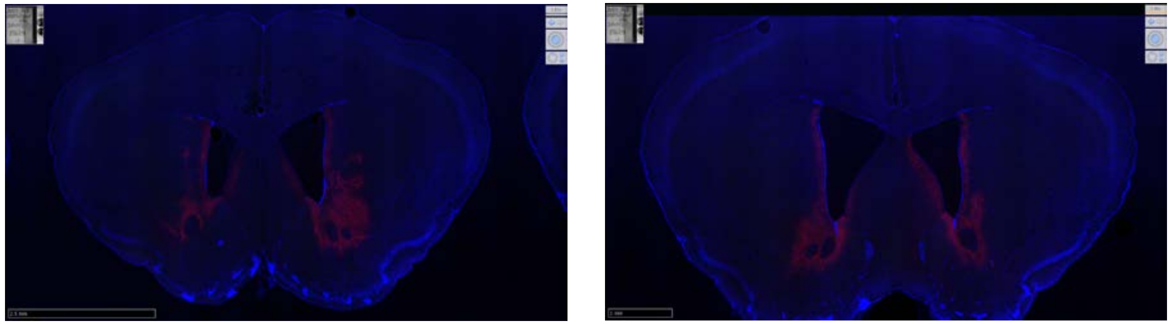

67215
D2gi

Missing image

67133
D2gi

Missing image

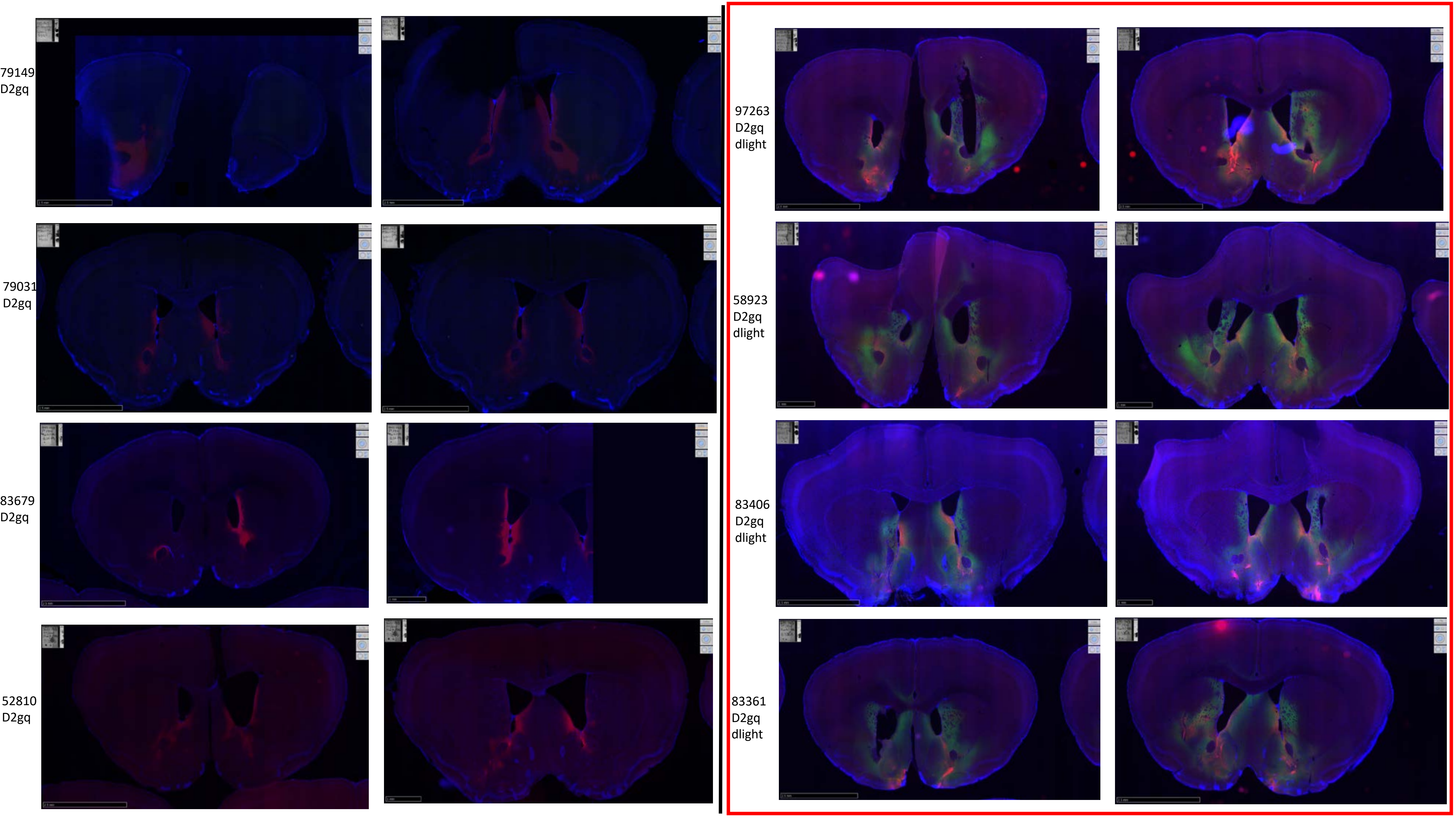
79149 D2gq
79031 D2gq
83679 D2gq
52810 D2gq
97263 D2gq dlight
58923 D2gq dlight
83406 D2gq dlight
83361 D2gq dlight

D1 cre mice (Fig 2F-H and Fig3C ; Gi=8, Gq=9)

10515
D1gq

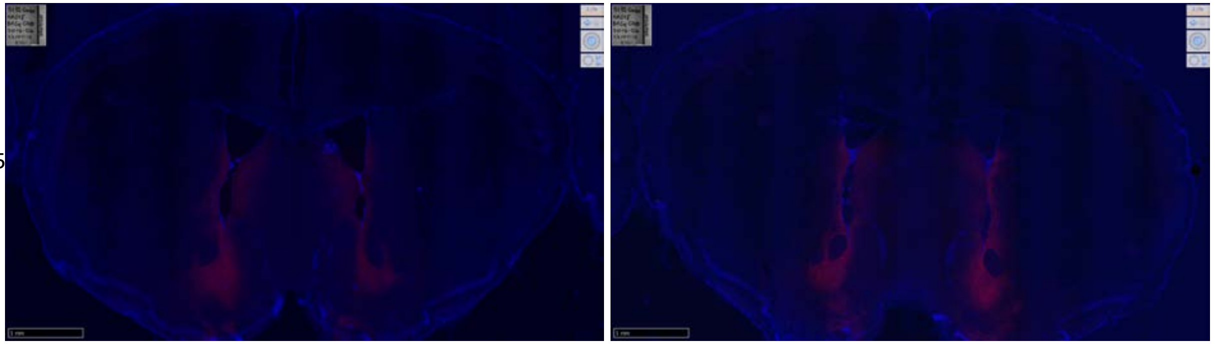

10961
D1gi

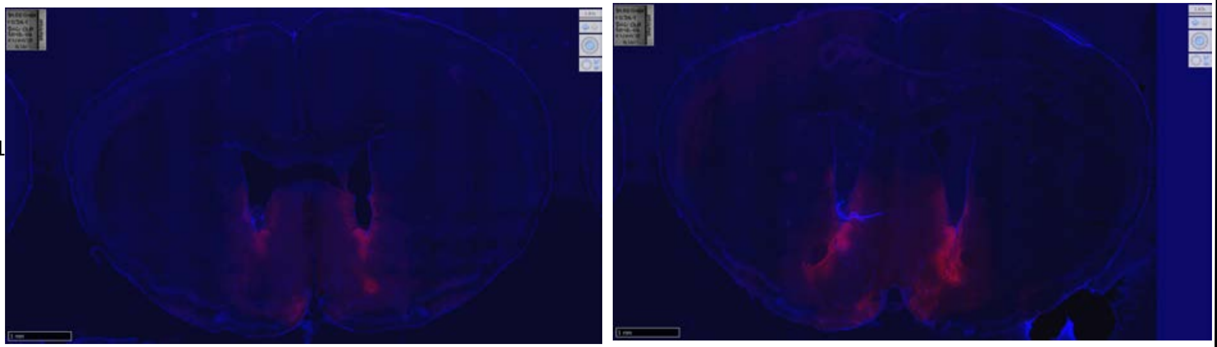

27381
D1gi

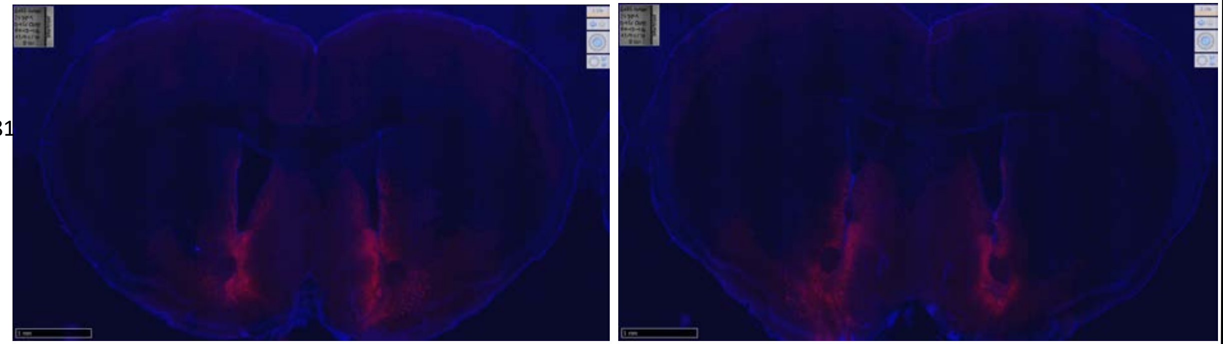

89928
D1gi

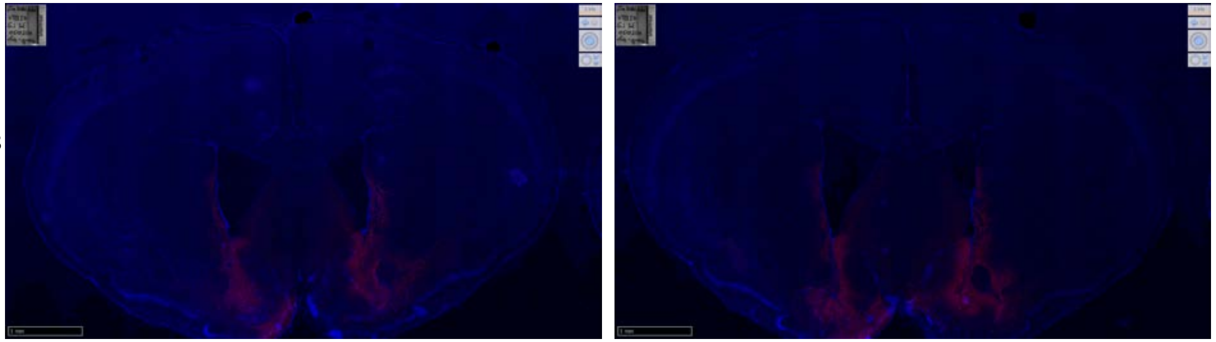

49131
D1gq

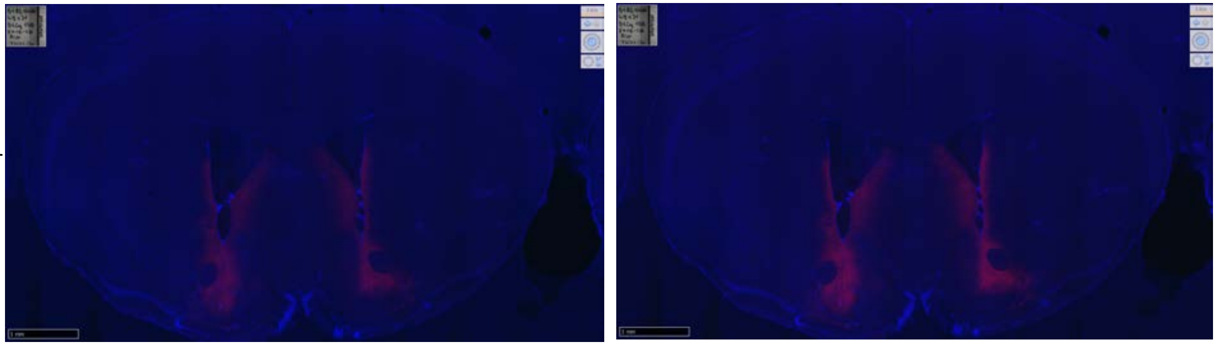

67063
D1gq

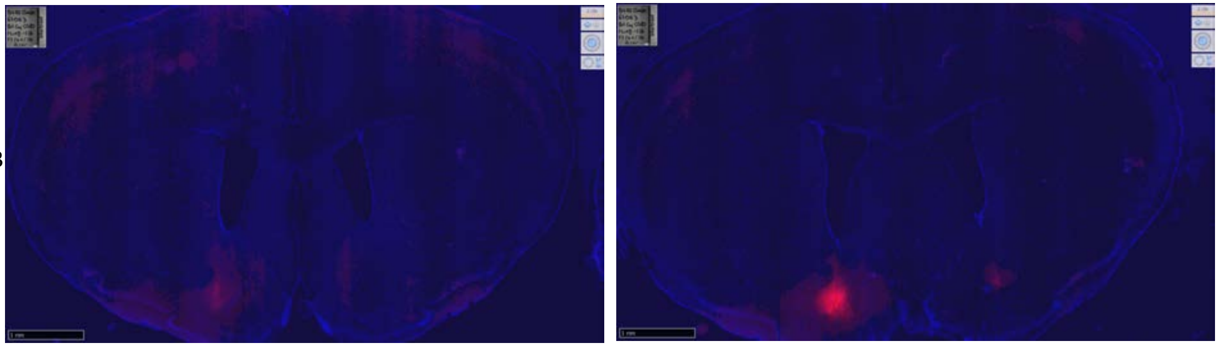

67075
D1gi

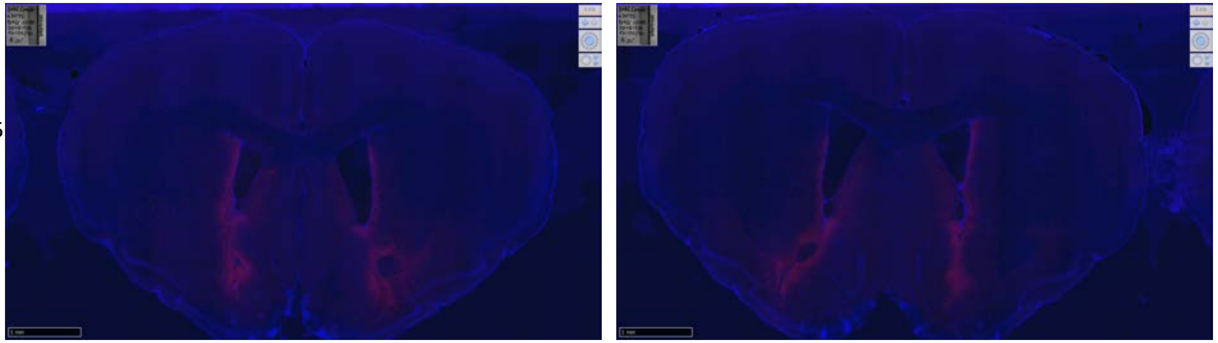

67171
D1gq

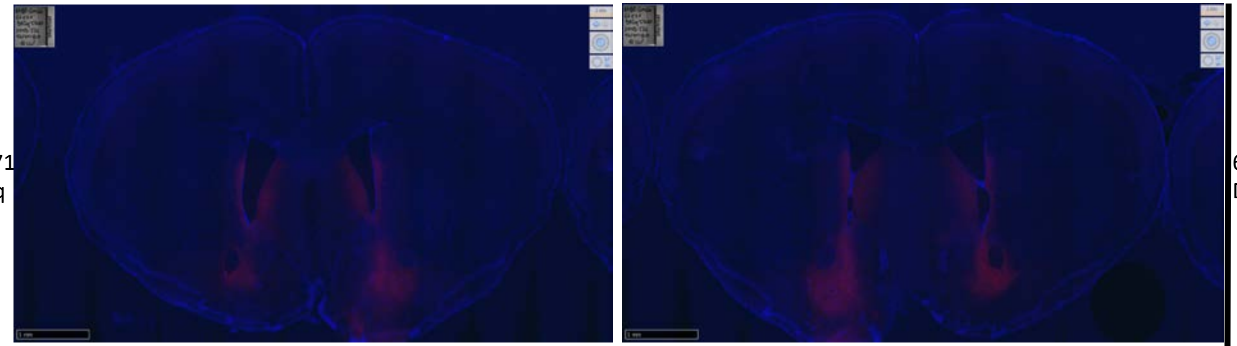

67328
D1gq

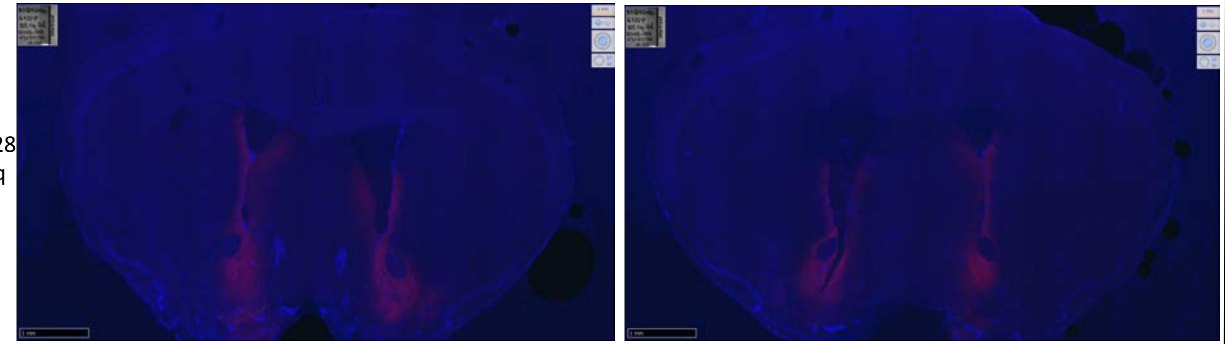

67375
D1gi

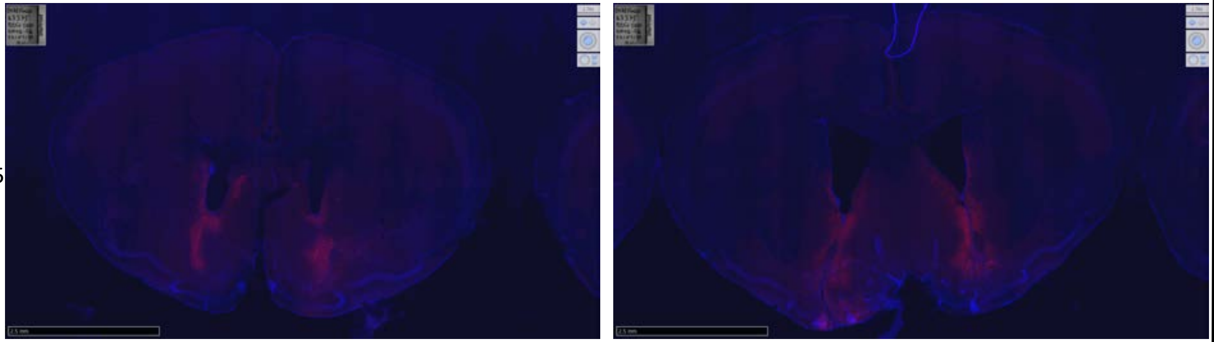

67397
D1gi

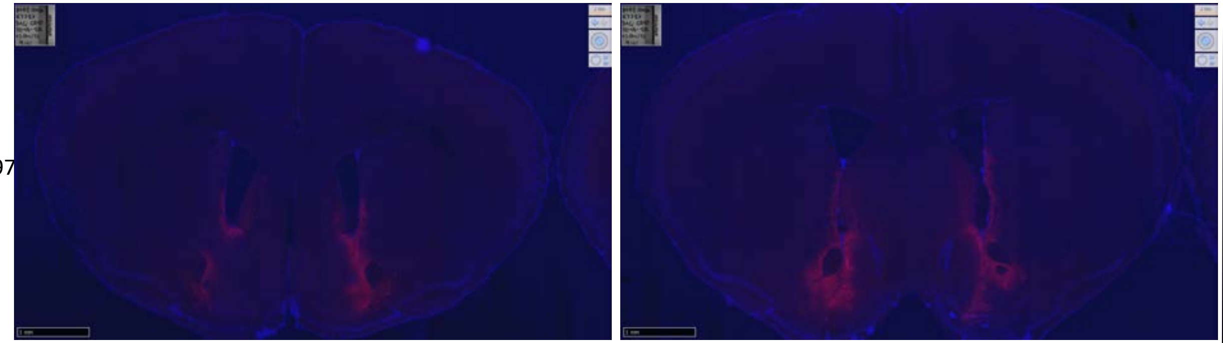

67414
D1gq

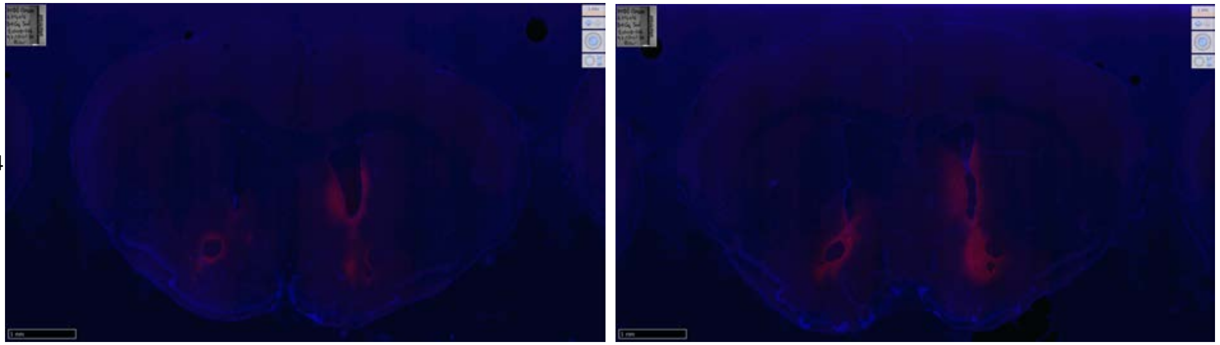

67454
D1gi

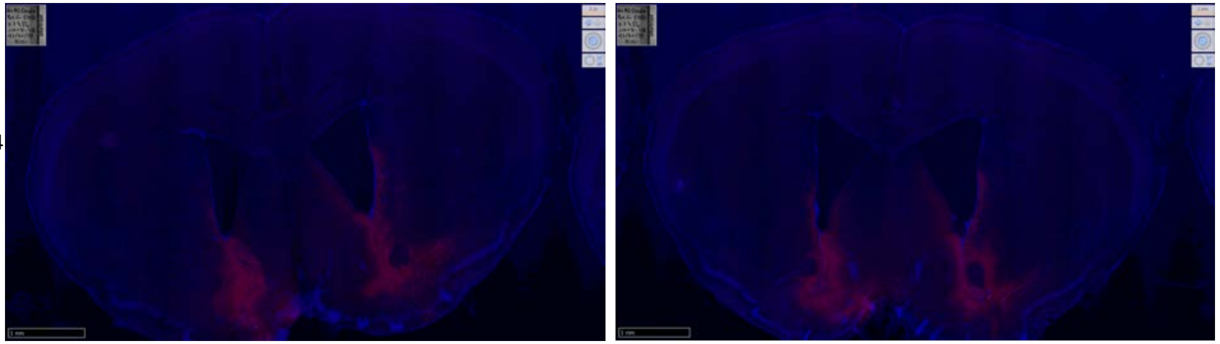

67488
D1gq

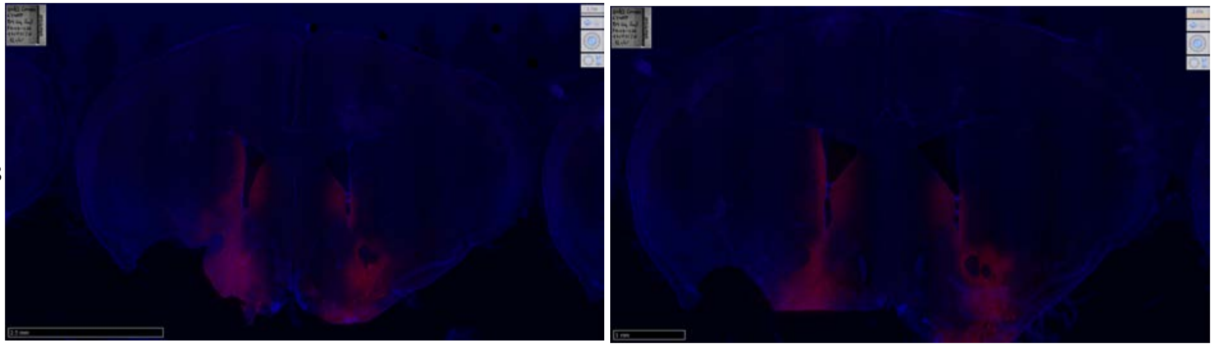

69872
D1gi

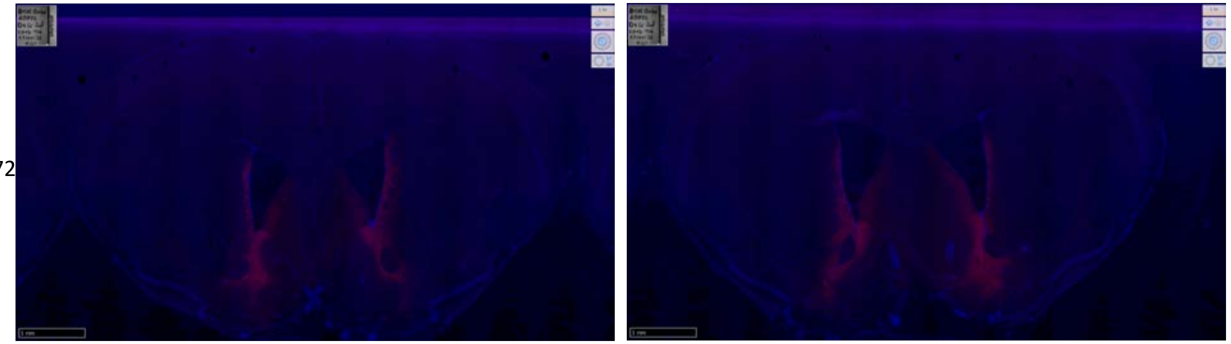

79156
D1gq

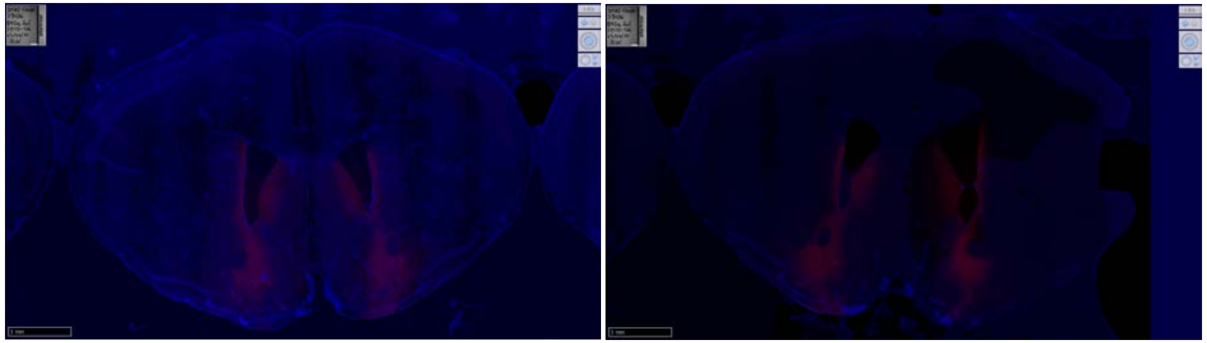

79327
D1gq

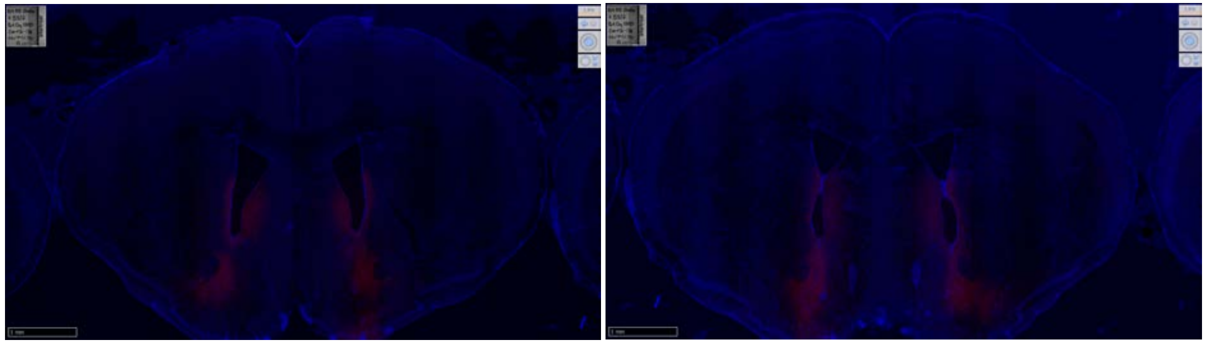

79494
D1gi

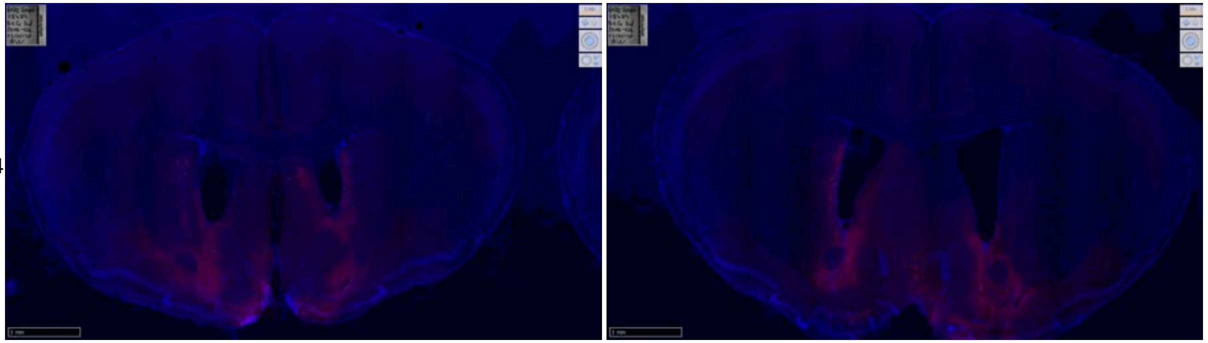

D2 cre mice (Fig 2B-D and Fig3B ; Gi=7 Gq=8)

2575
D2gi

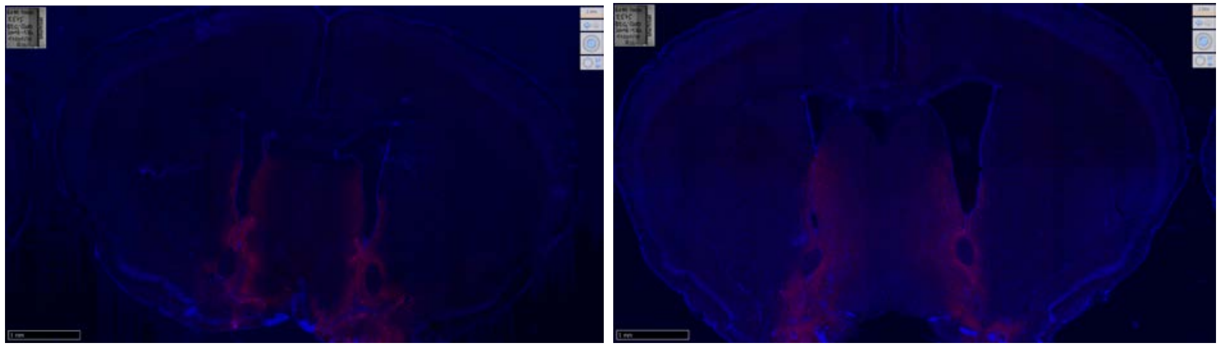

27066
D2gi

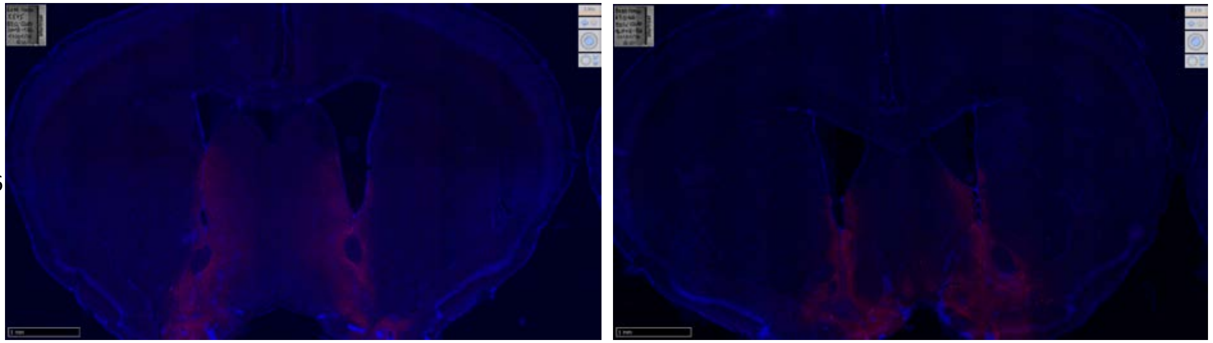

67113
D2gq

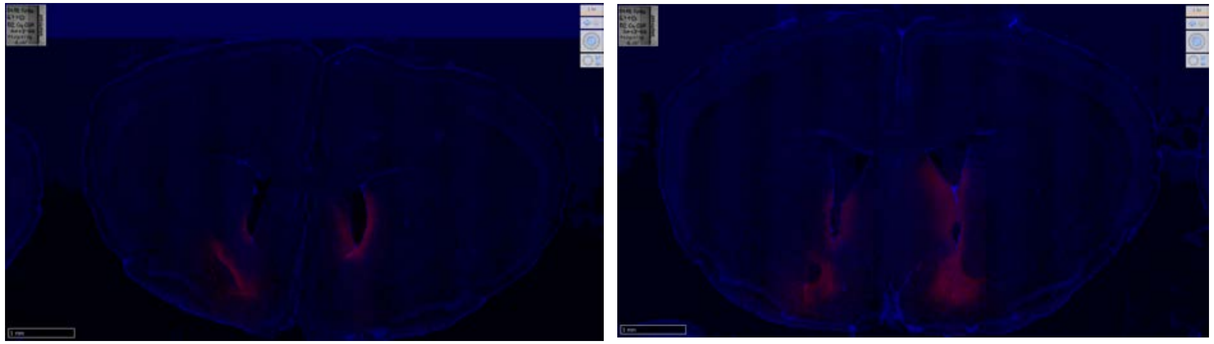

67123
D2gq

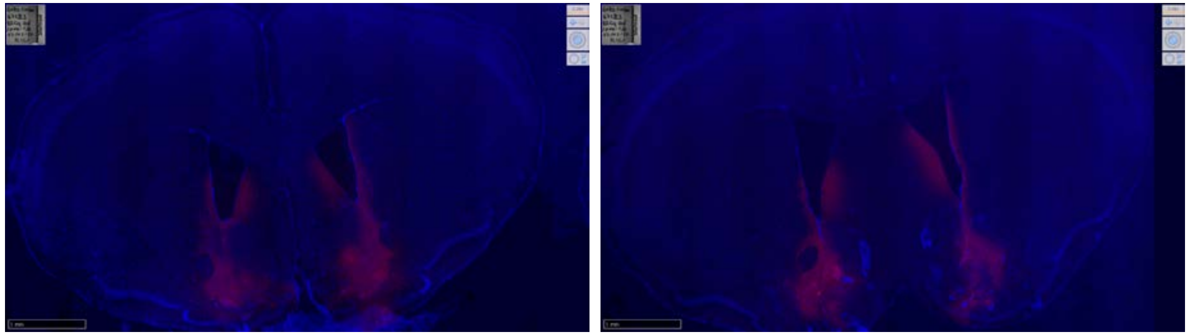

67233
D2gq

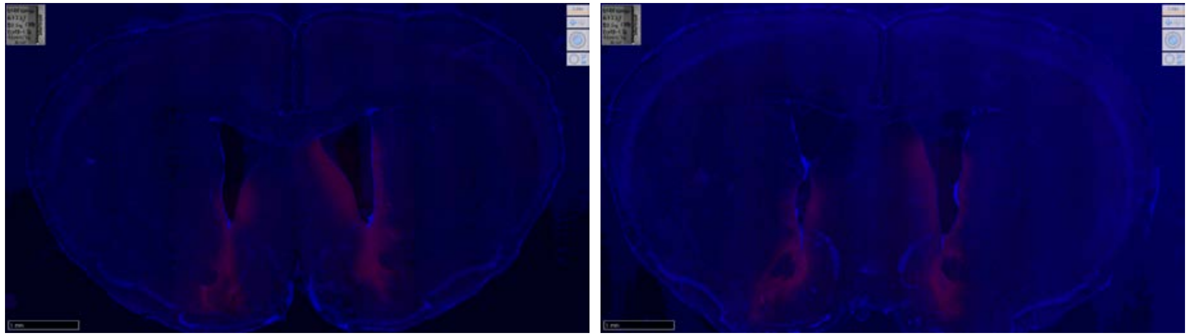

67246
D2gq

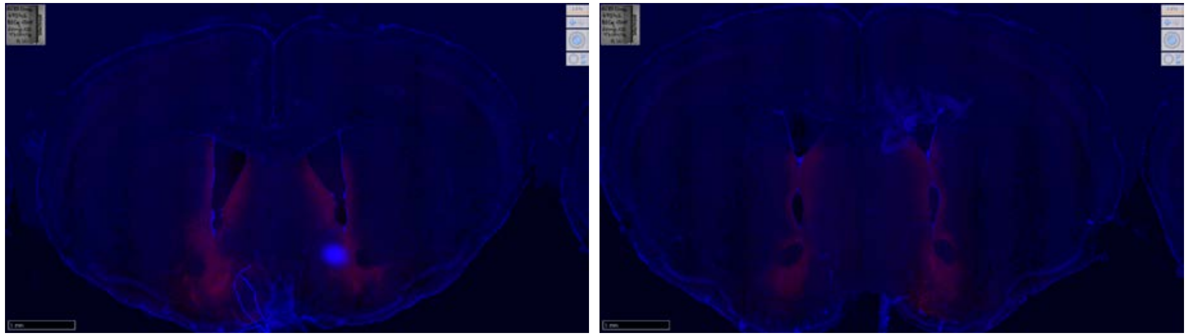

67249
D2gi

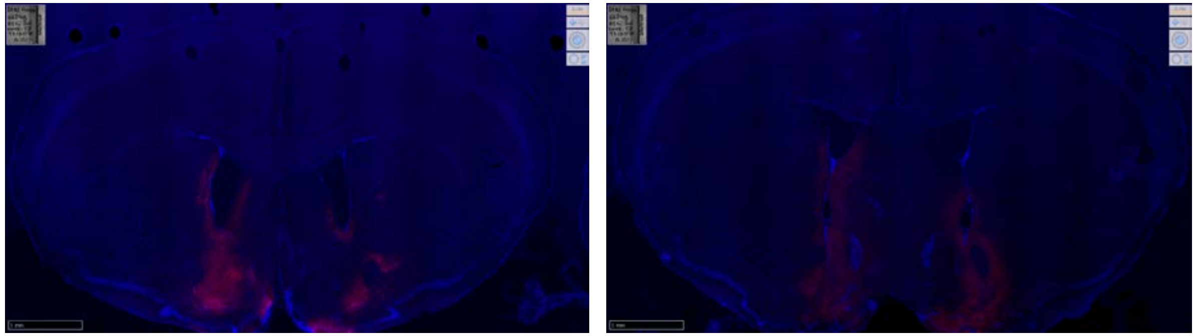

67255
D2gi

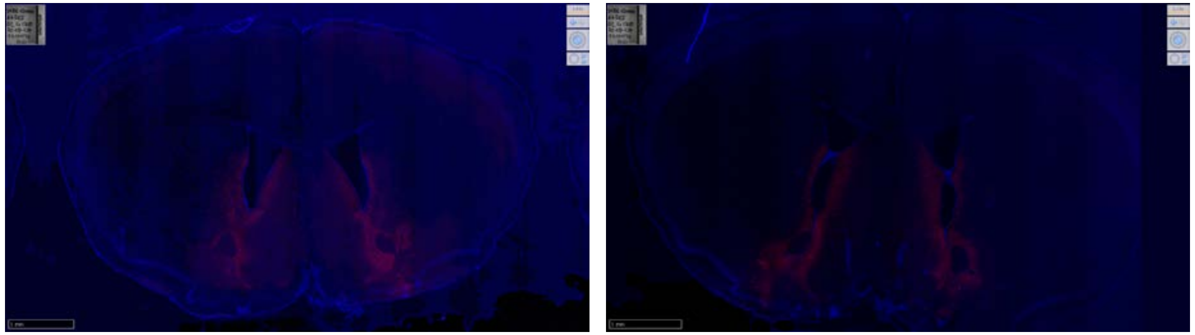

67319
D2gi

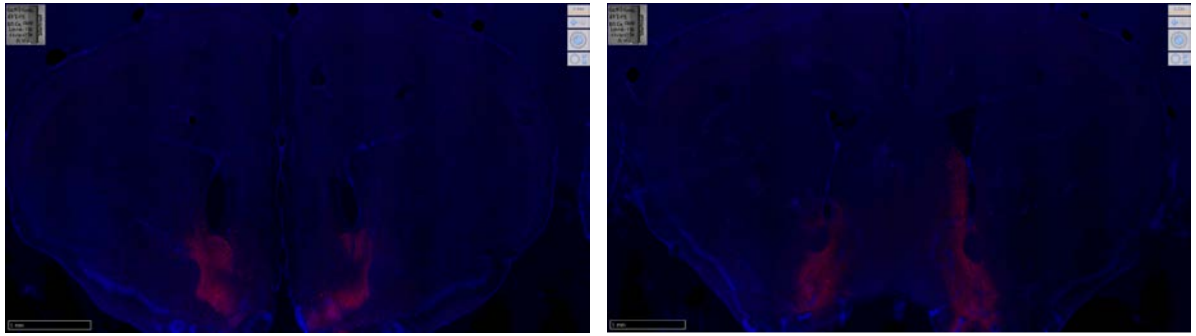

67331
D2gi

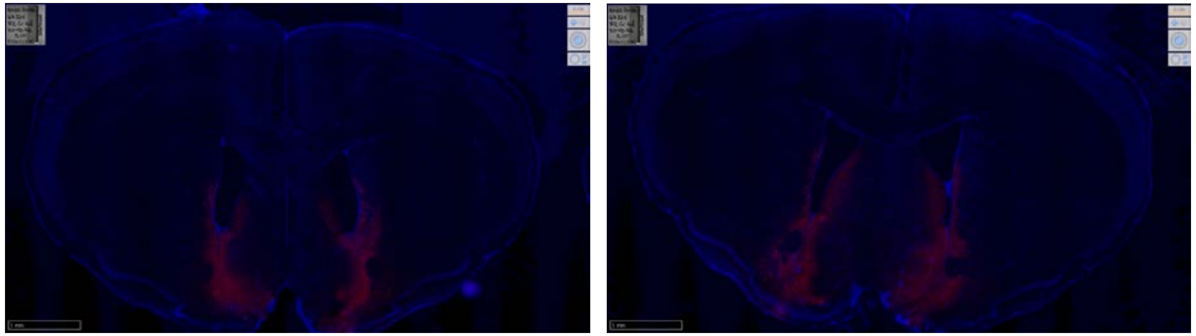

67456
D2gq

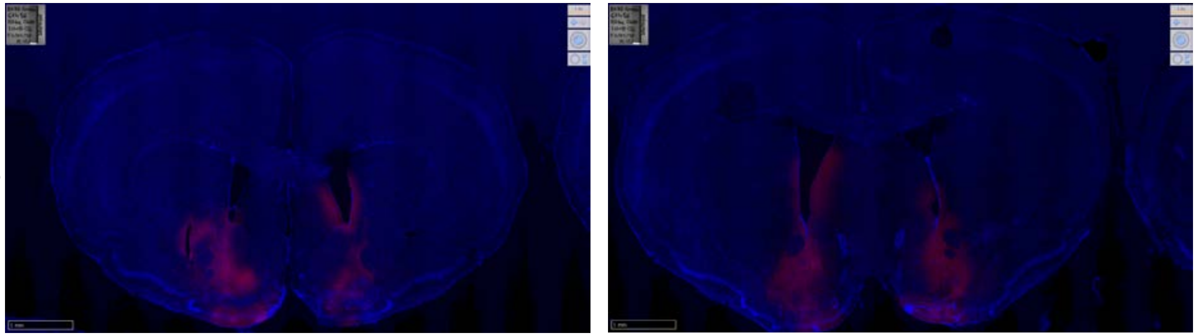

67496
D2gi

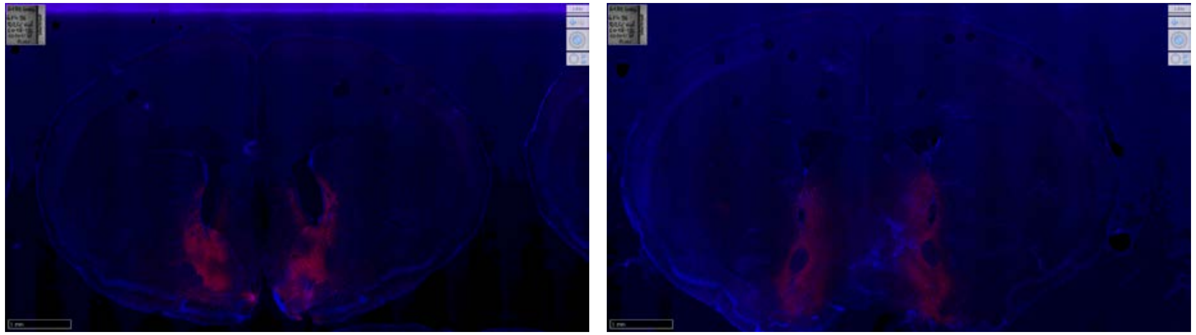

79062
D2gq

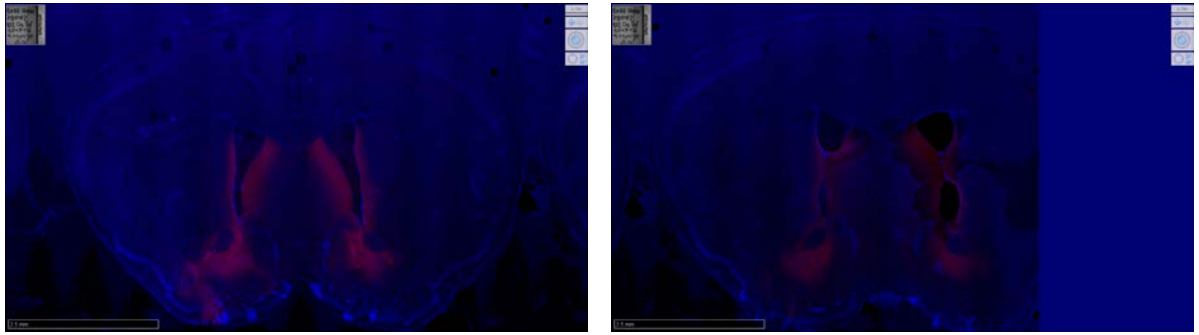

97084
D2gq

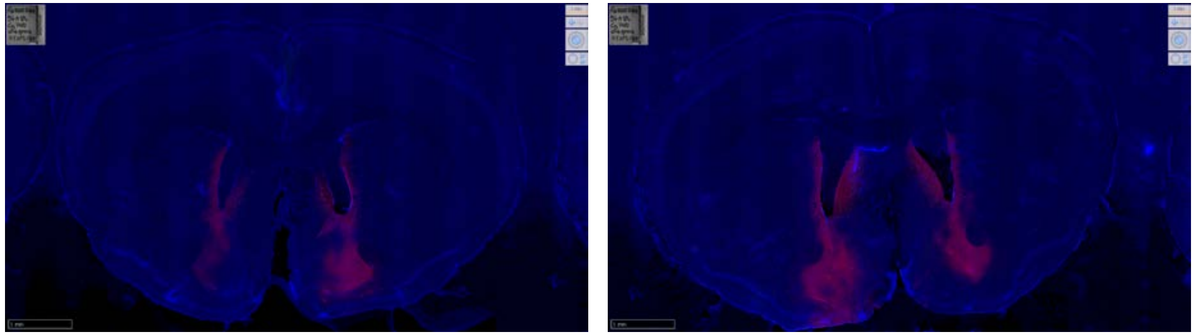

97491
D2gq

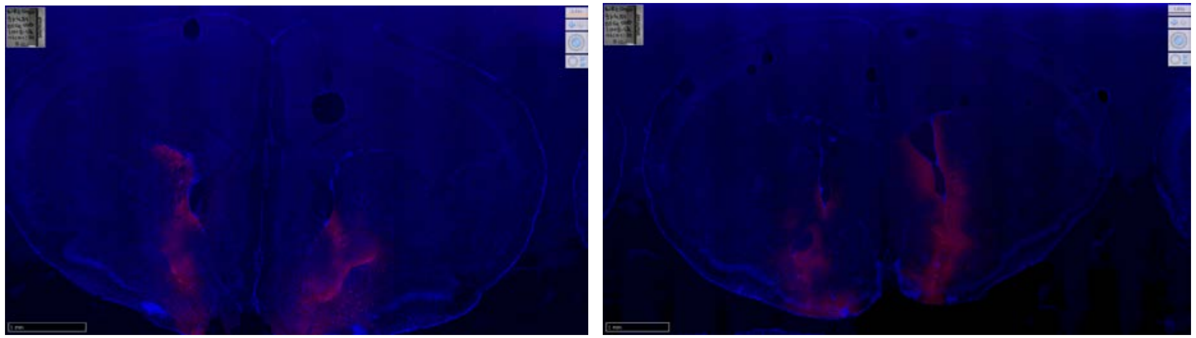

D1 cre mice (Fig 2 C-D Fig 3E-F)

58667
D1
Gcamp

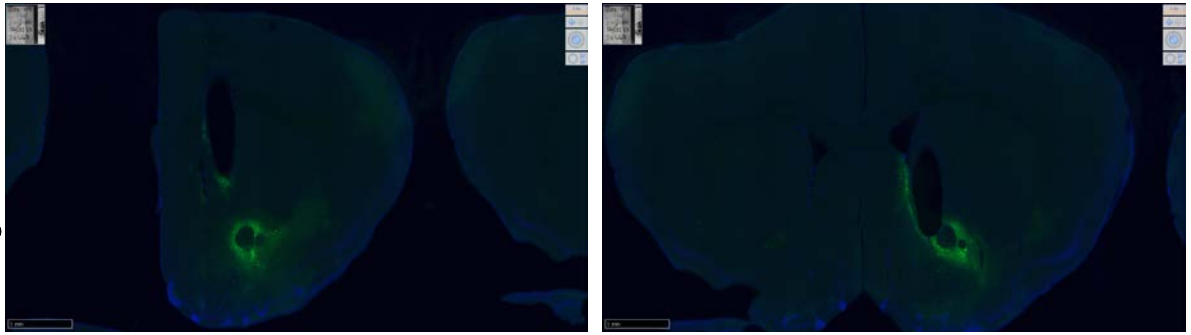

58817
D1
Gcamp

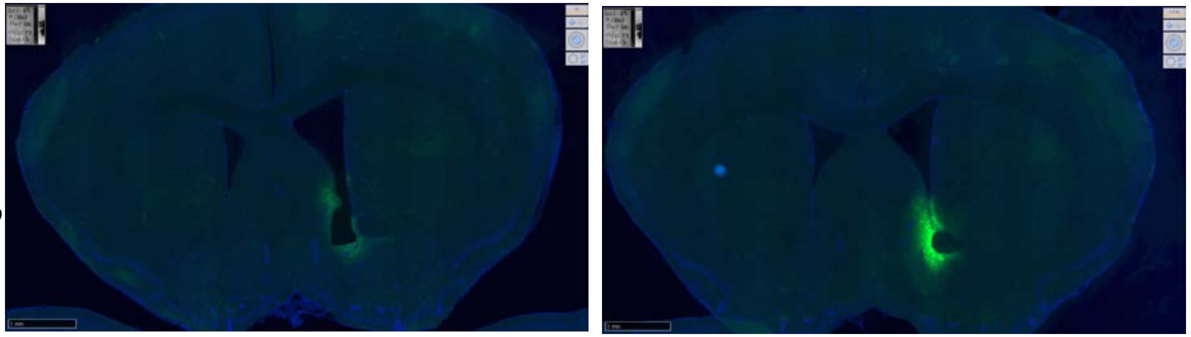

83436
D1
Gcamp

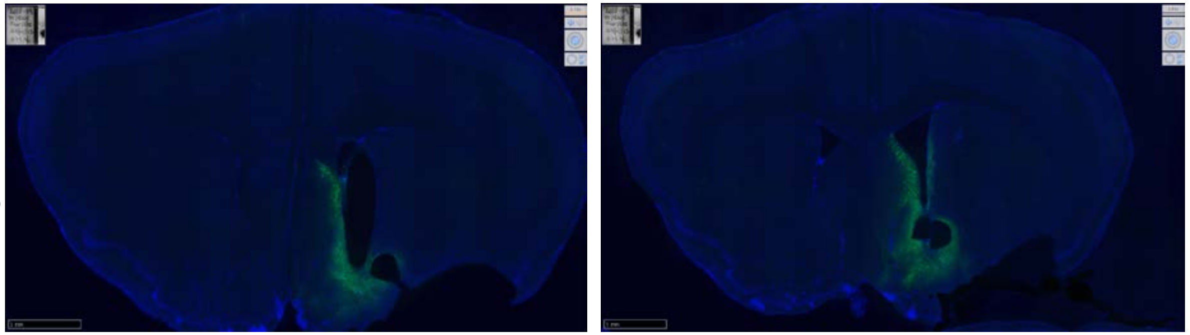

97484
D1
Gcamp

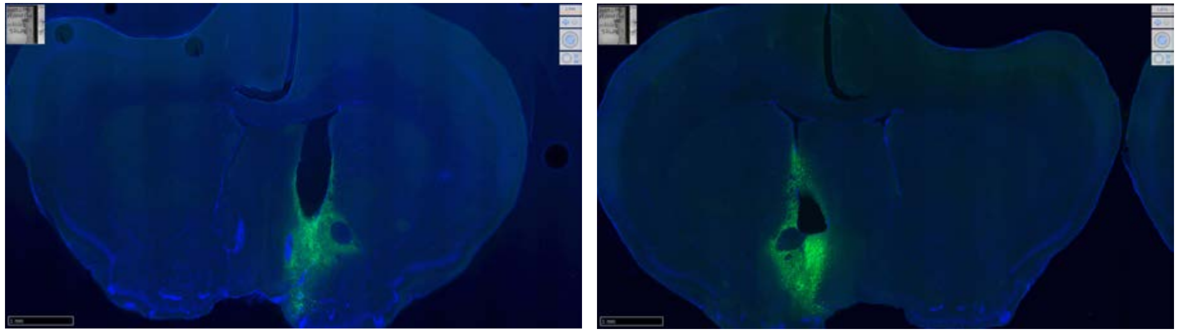

69738
D1
Gcamp

Missing
image

D2 cre mice (Fig 2 C-D Fig 3E-F)

58940
D2
Gcamp

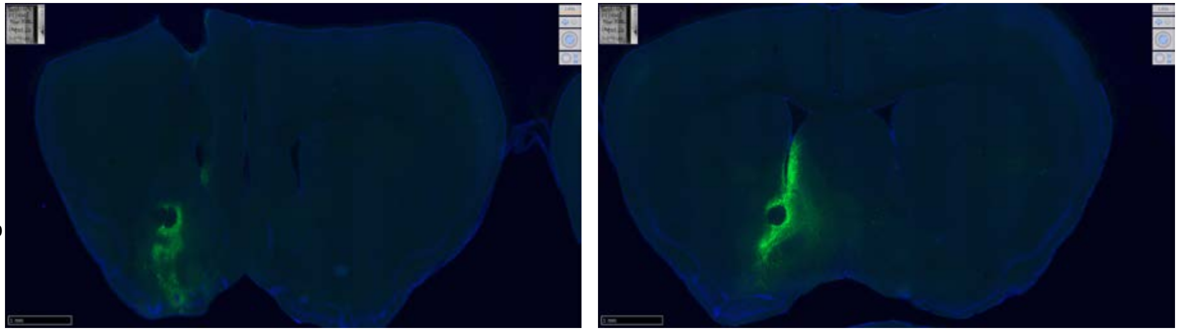

58640
D2
Gcamp

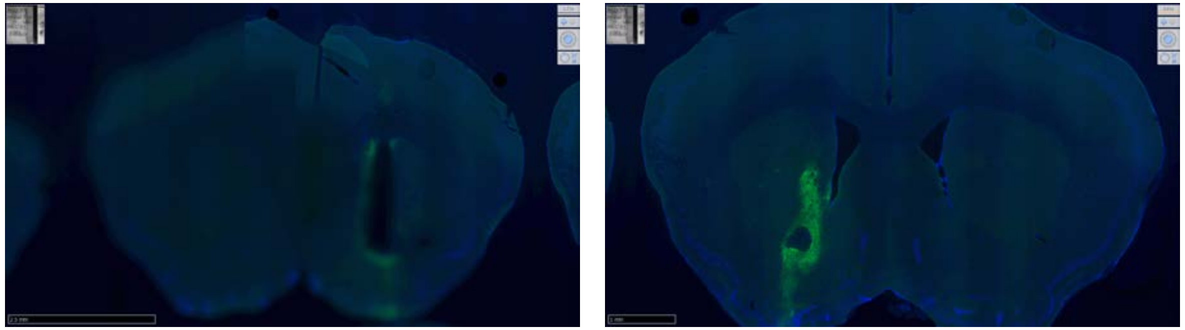

58717
D2
Gcamp

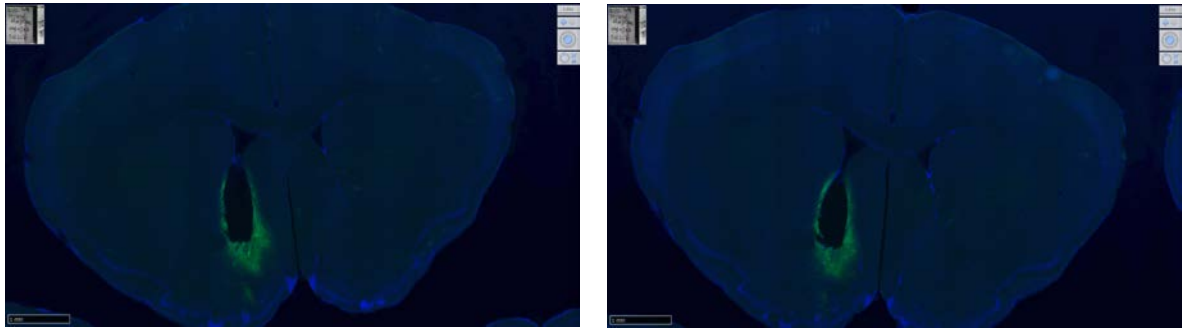

58768
D2
Gcamp

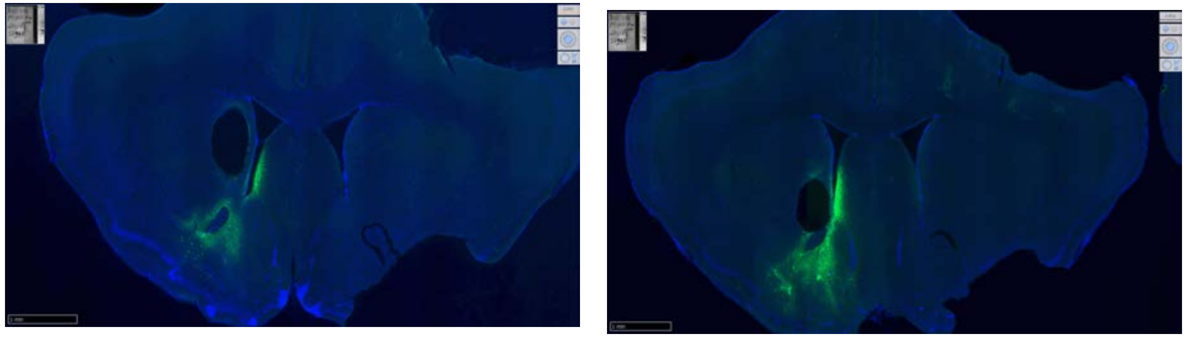

67335
D2
Gcamp

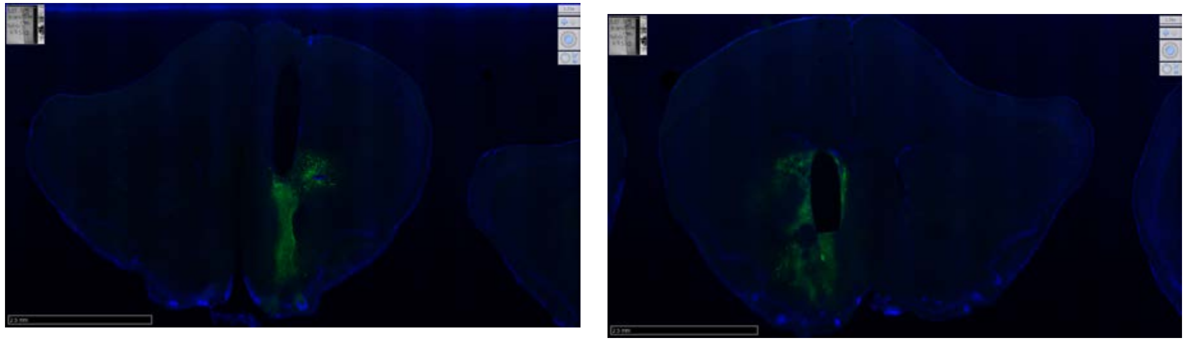

D1 cre mice (Fig 4E-G; Gi = 11 Gq=12)

27175
D1Gi

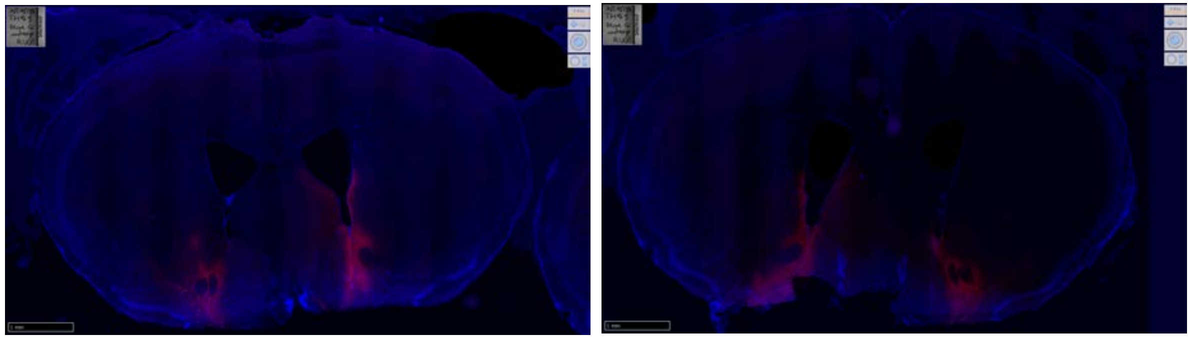

27191
D1Gq

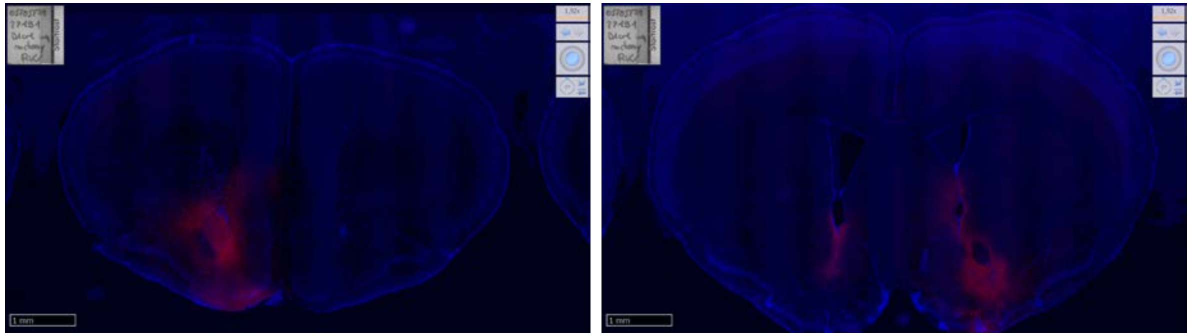

27183
D1Gi

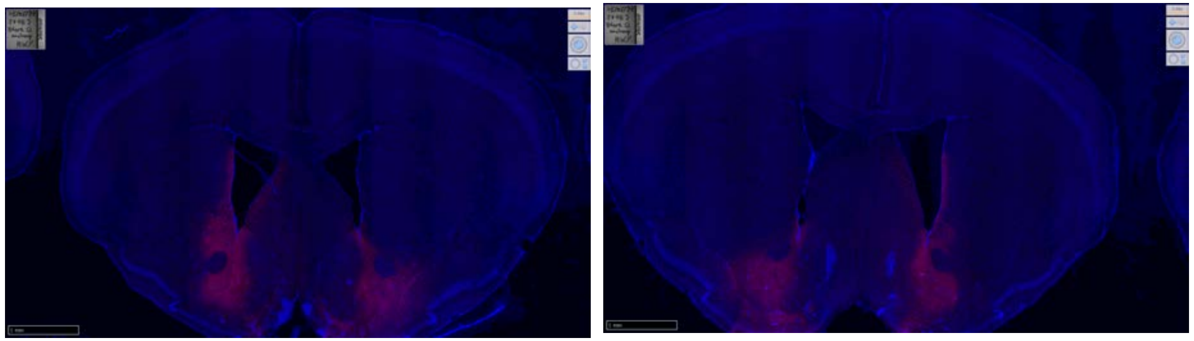

27343
D1Gi

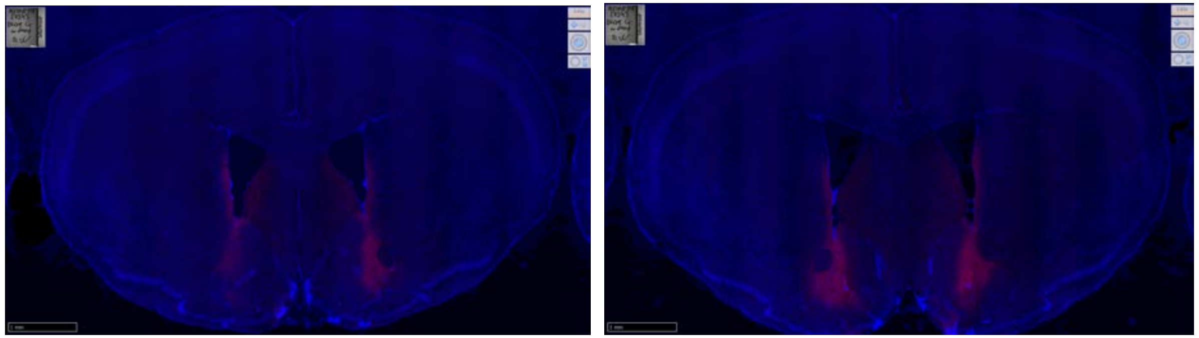

45320
D1Gq

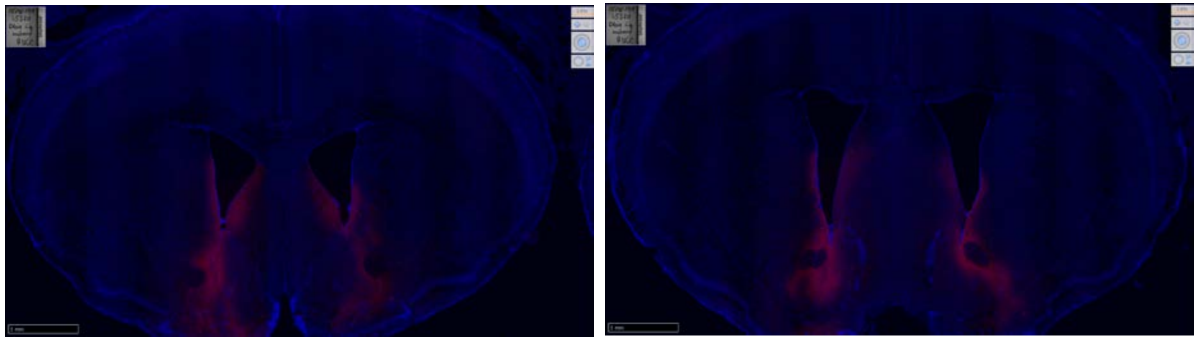

52486
D2Gi

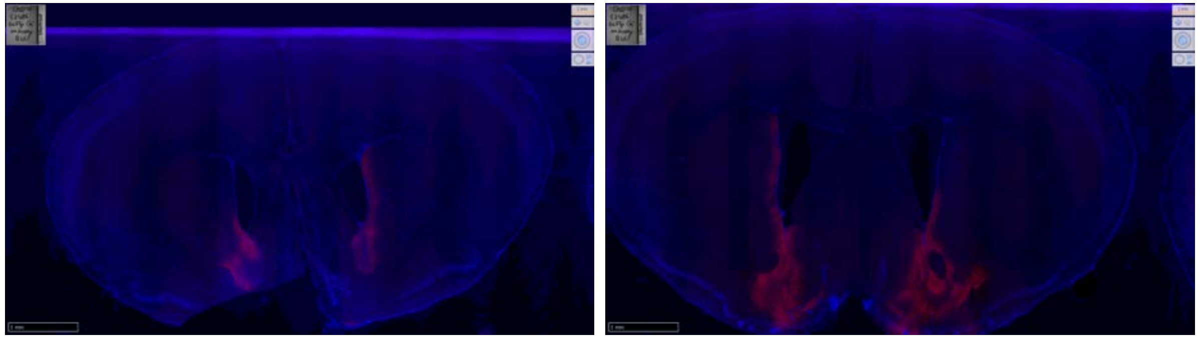

52489
D1Gi

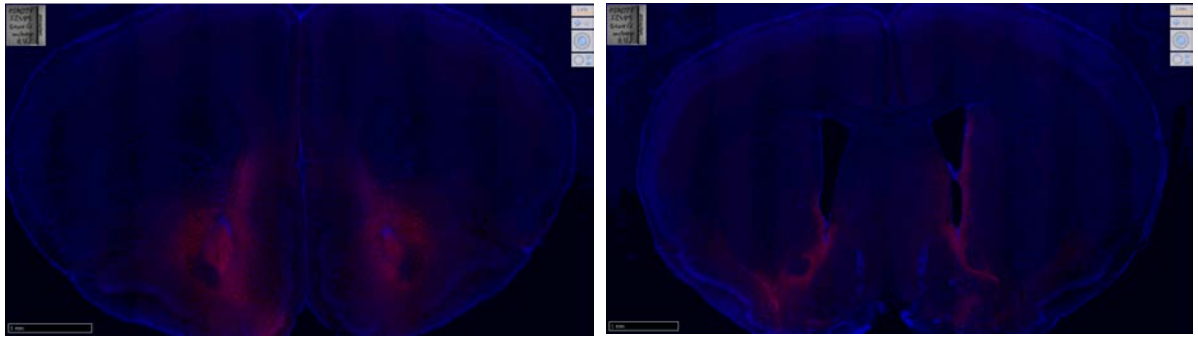

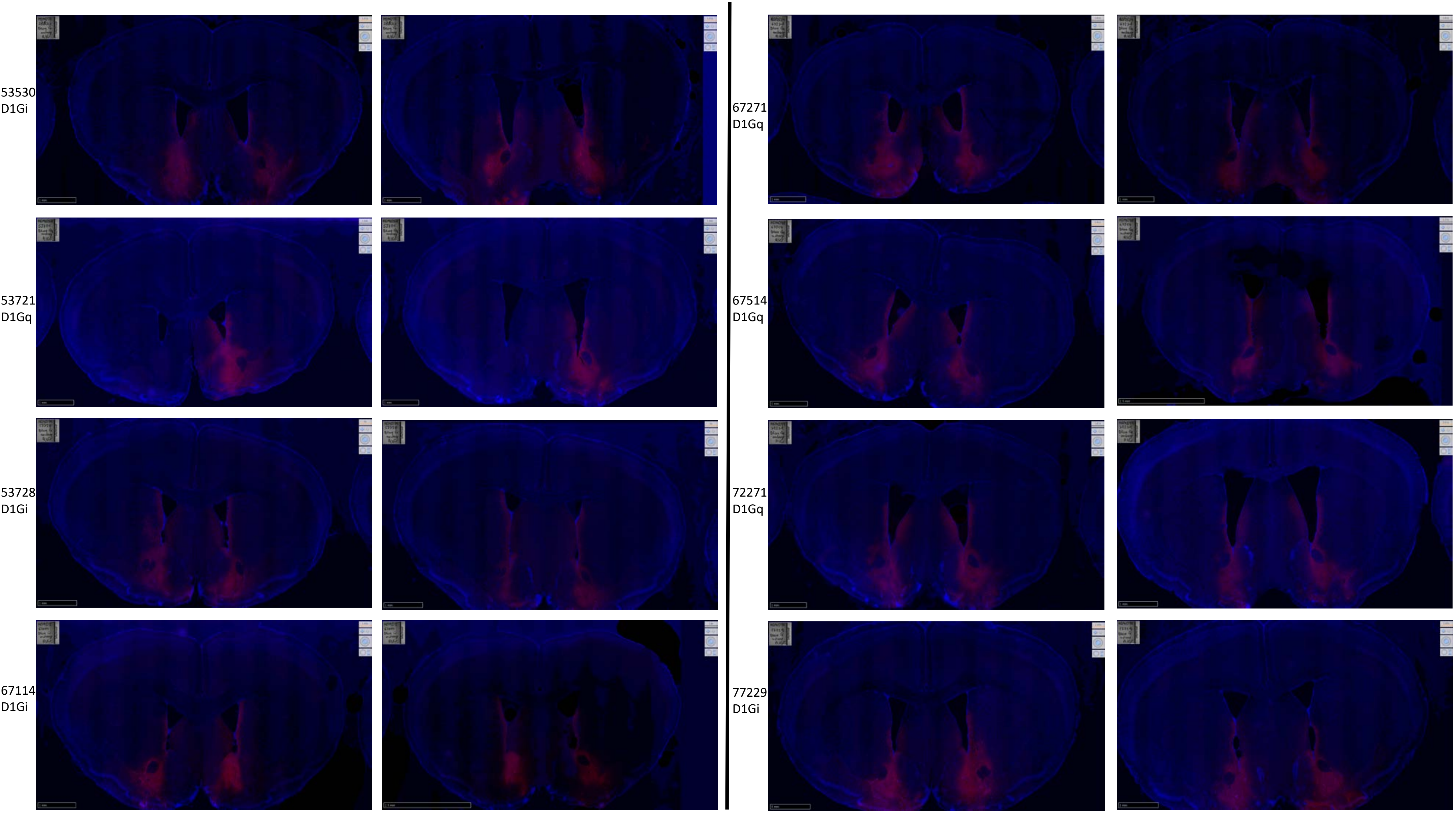
53530
D1Gi
53721
D1Gq
53728
D1Gi
67114
D1Gi
67271
D1Gq
67514
D1Gq
72271
D1Gq
77229
D1Gi

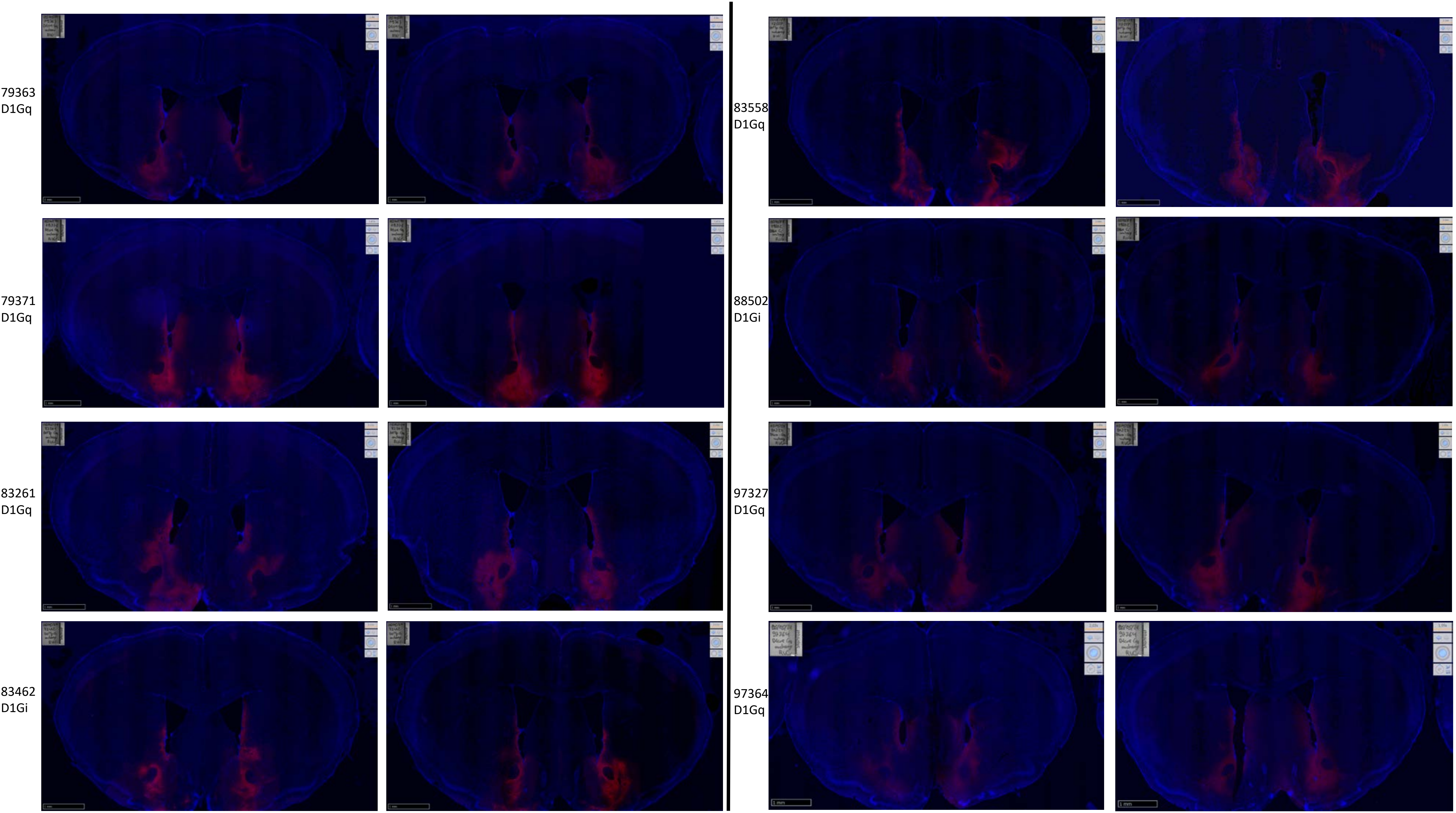
79363 D1Gq
79371 D1Gq
83261 D1Gq
83462 D1Gi
83558 D1Gq
88502 D1Gi
97327 D1Gq
97364 D1Gq

D2 cre mice (Fig 4E-G ; Gi = 8 Gq=9)

45423
D2Gq

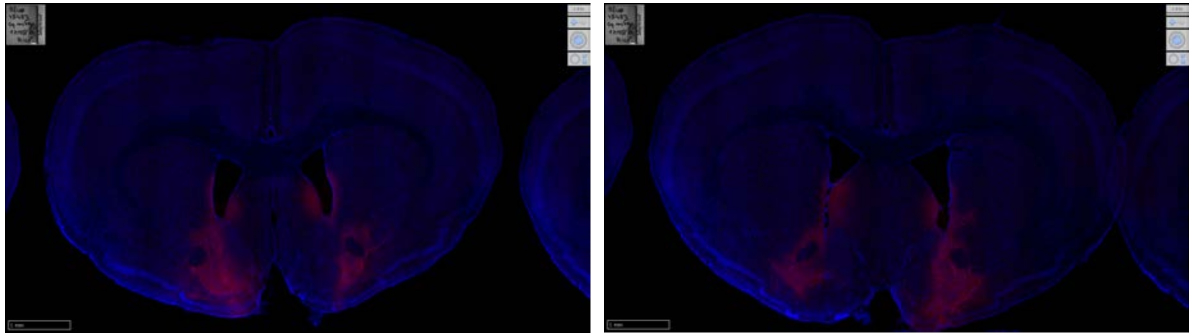

46967
D2Gi

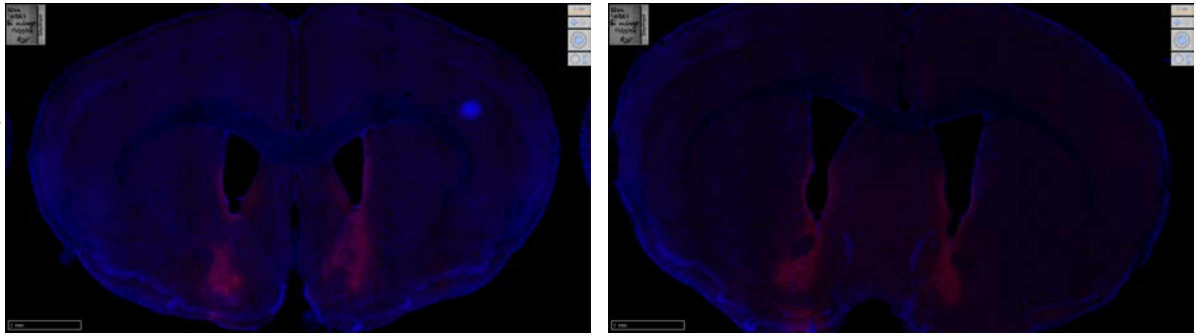

52944
D2Gq

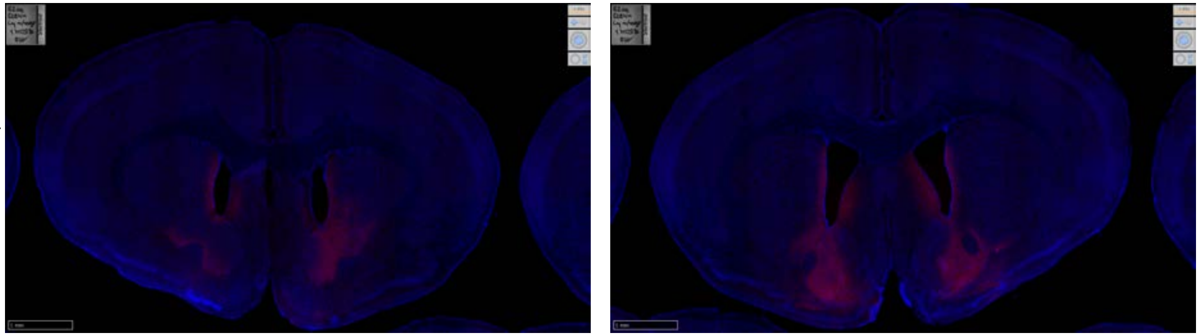

53570
D2Gi

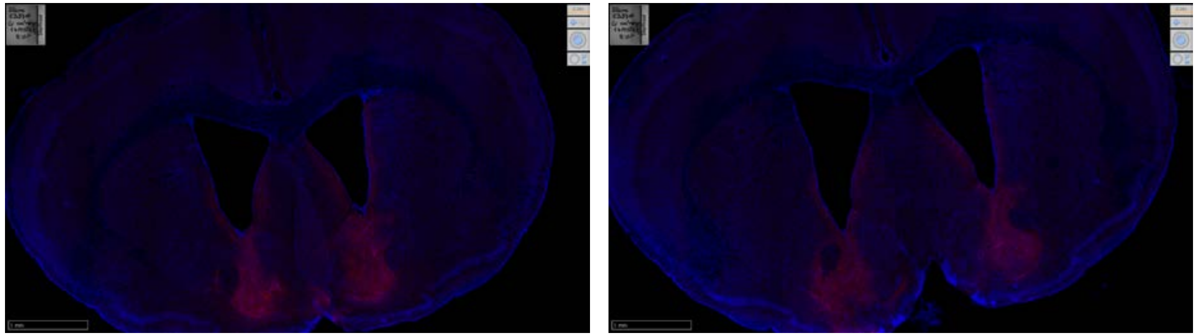

53571
D2Gi

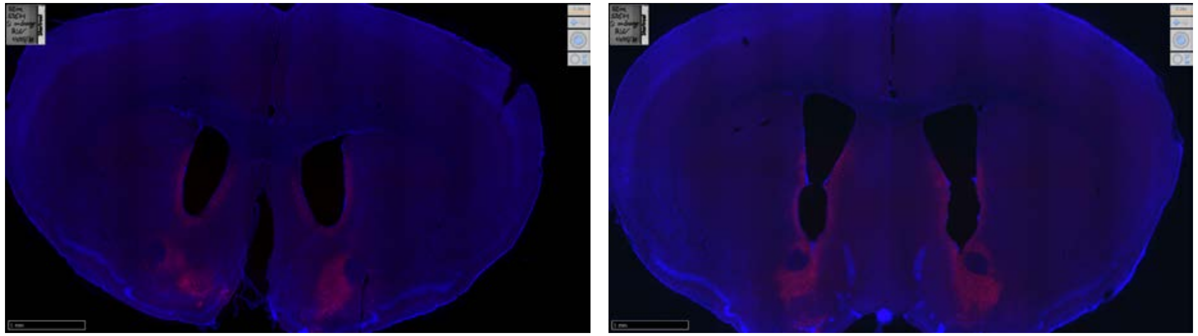

67133
D2Gq

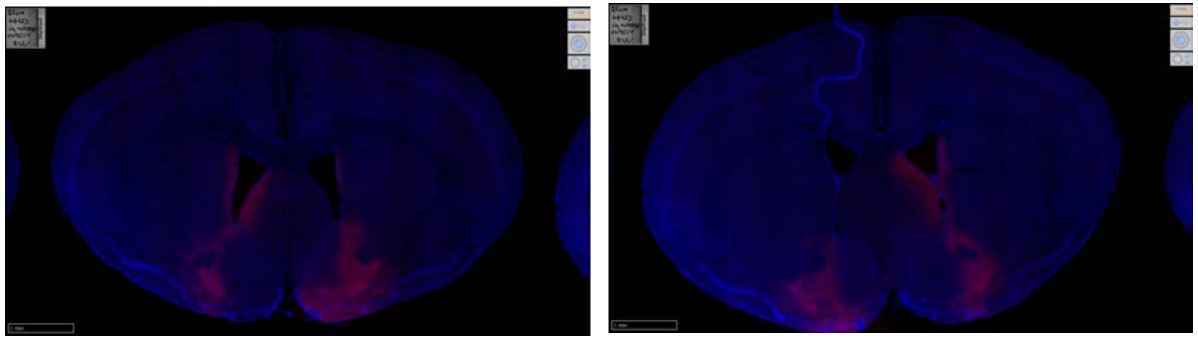

67268
D2Gi

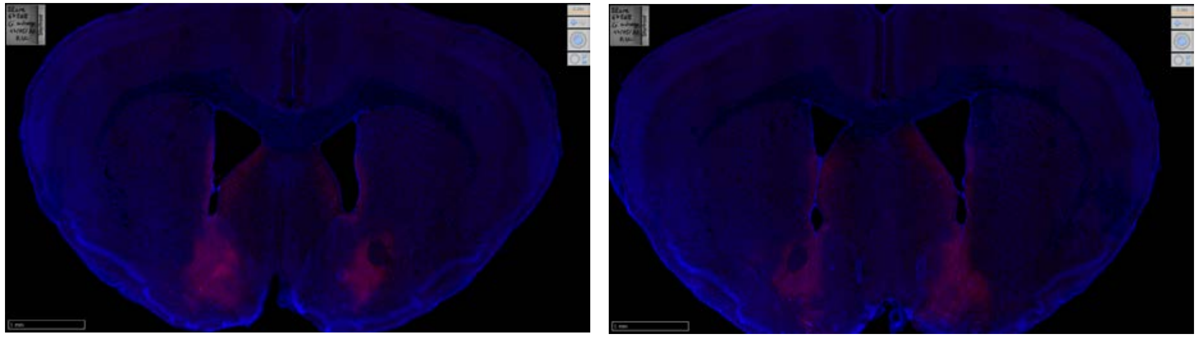

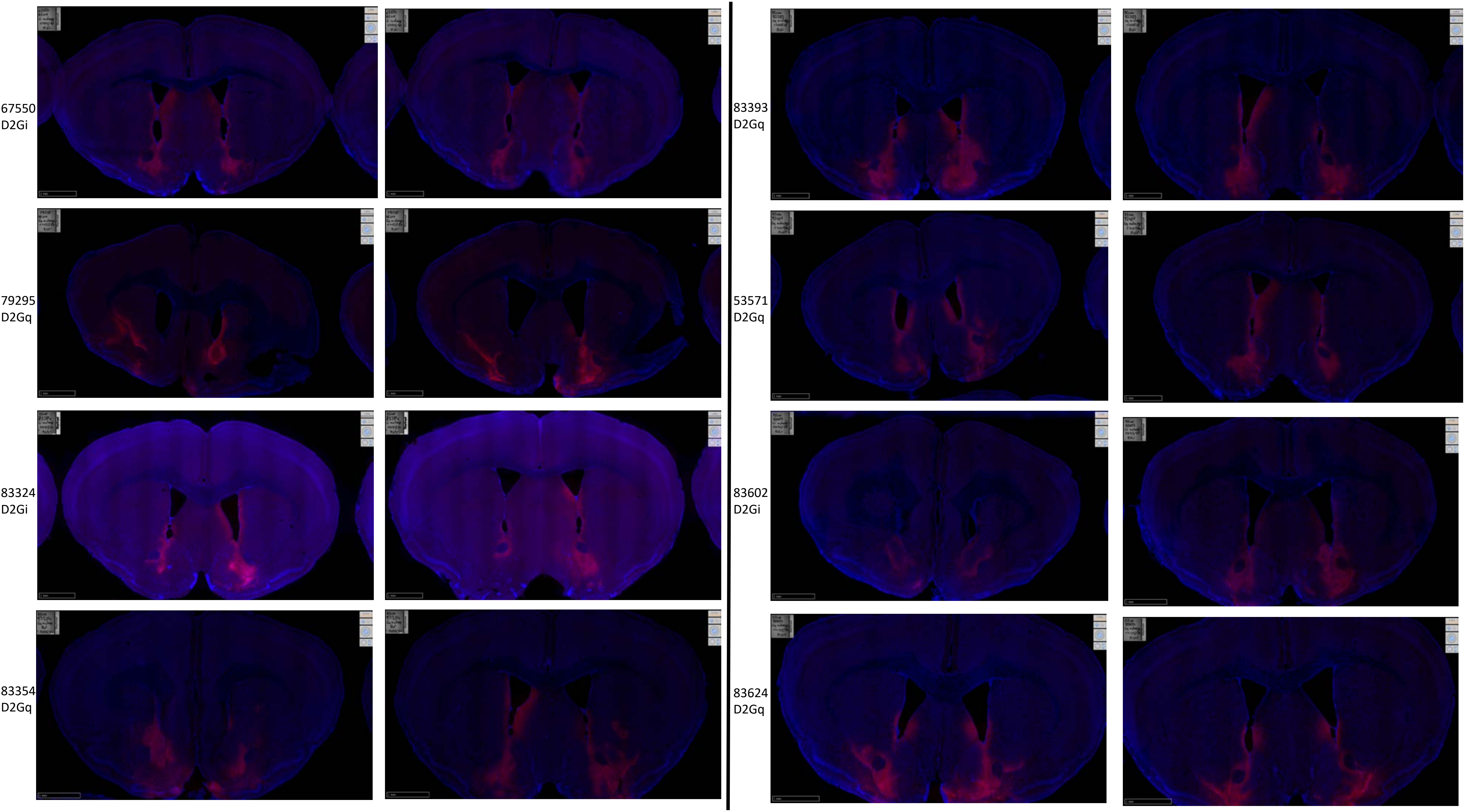
67550
D2Gi
79295
D2Gq
83324
D2Gi
83354
D2Gq
83393
D2Gq
53571
D2Gq
83602
D2Gi
83624
D2Gq

87637
D2Gi

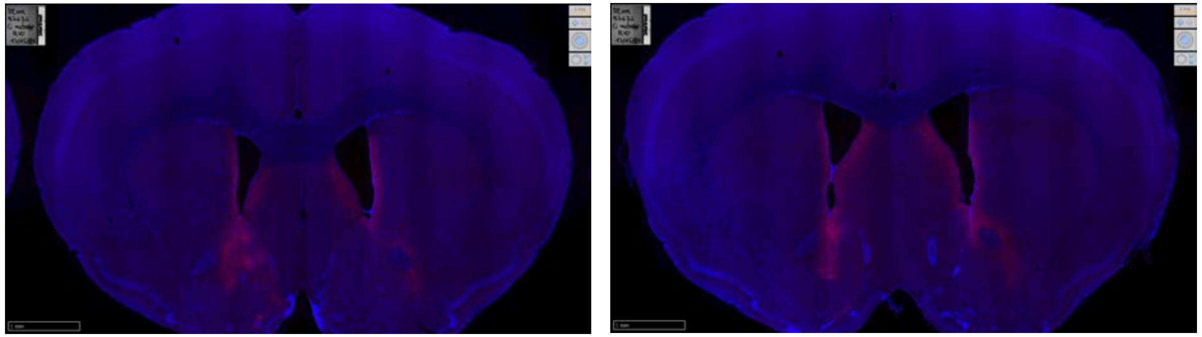

89164
D2Gq

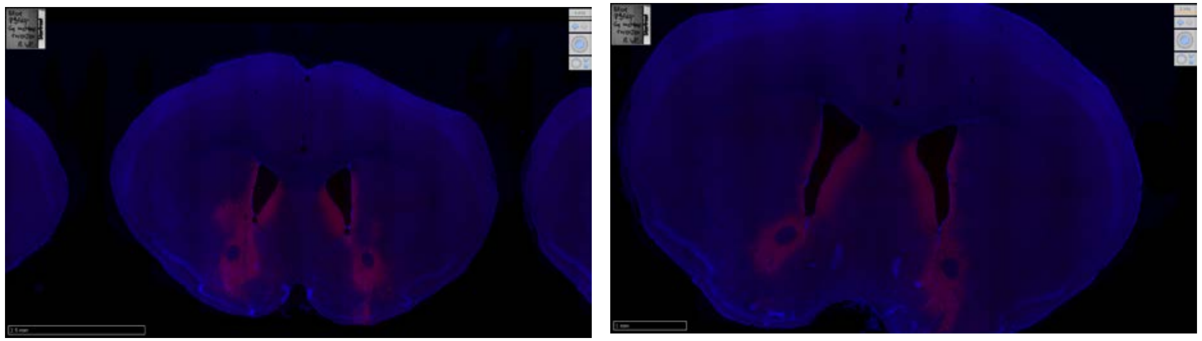

D1Flp/D2cre mice (Fig 5 ; D1GiD2Gq = 11, D1GiD2Gq = 8)

10650
D1Gq
D2Gi

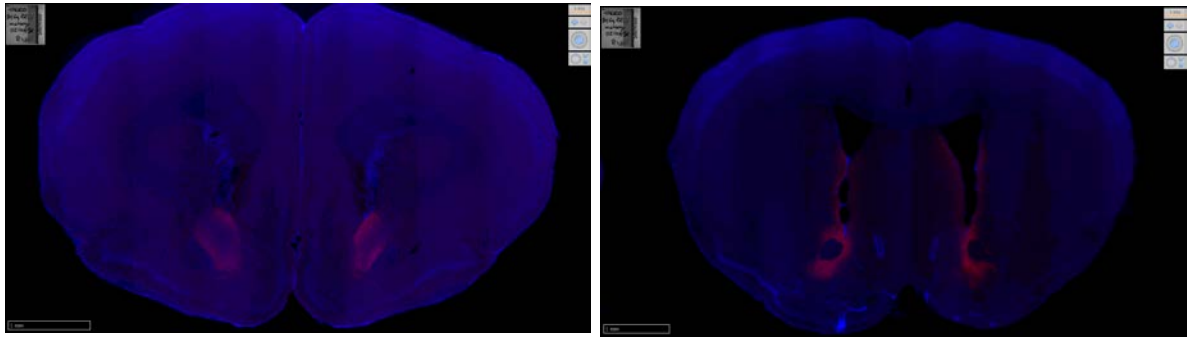

24147
D1Gq
D2Gi

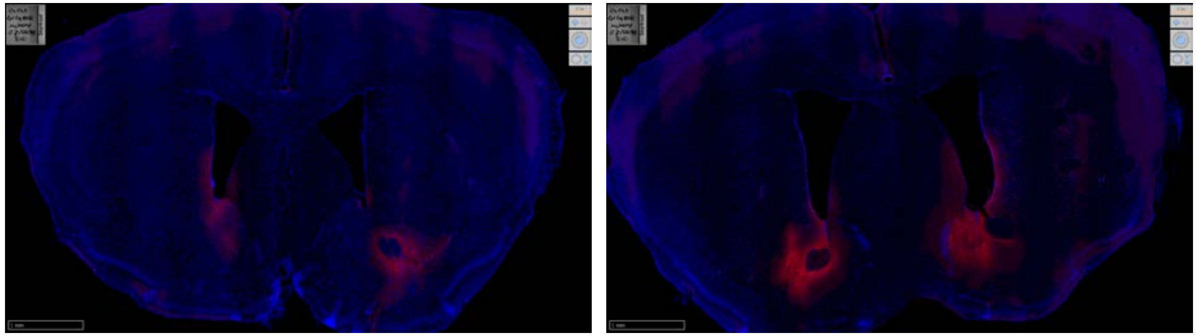

27091
D1Gq
D2Gi

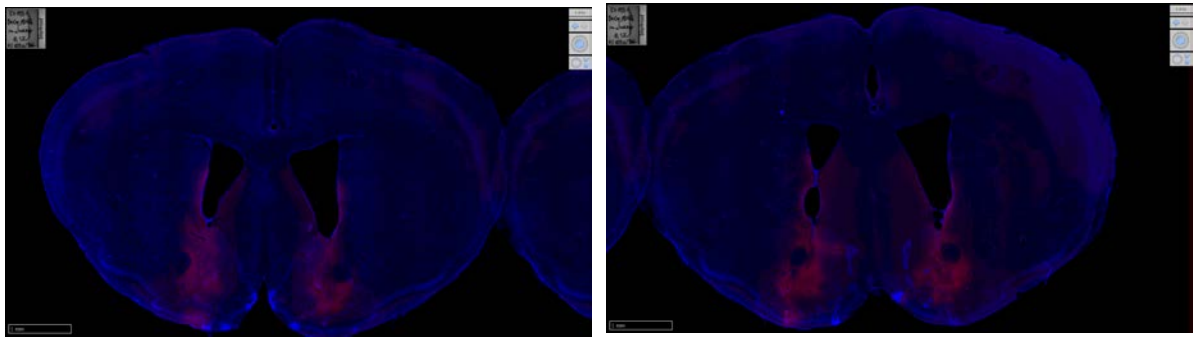

27503
D1Gq
D2Gi

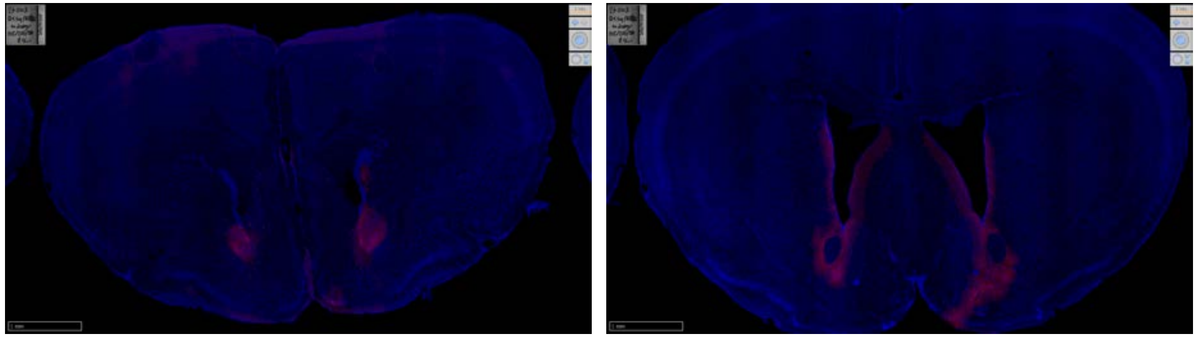

67094
D1Gi
D2Gq

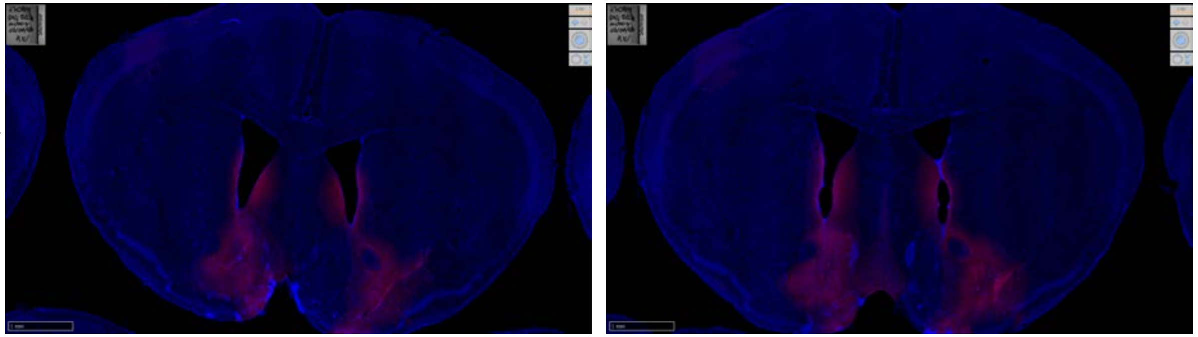

79218
D1Gq
D2Gi

79387
D1Gq
D2Gi

83383
D1Gi
D2Gq

83380
D1Gi
D2Gq

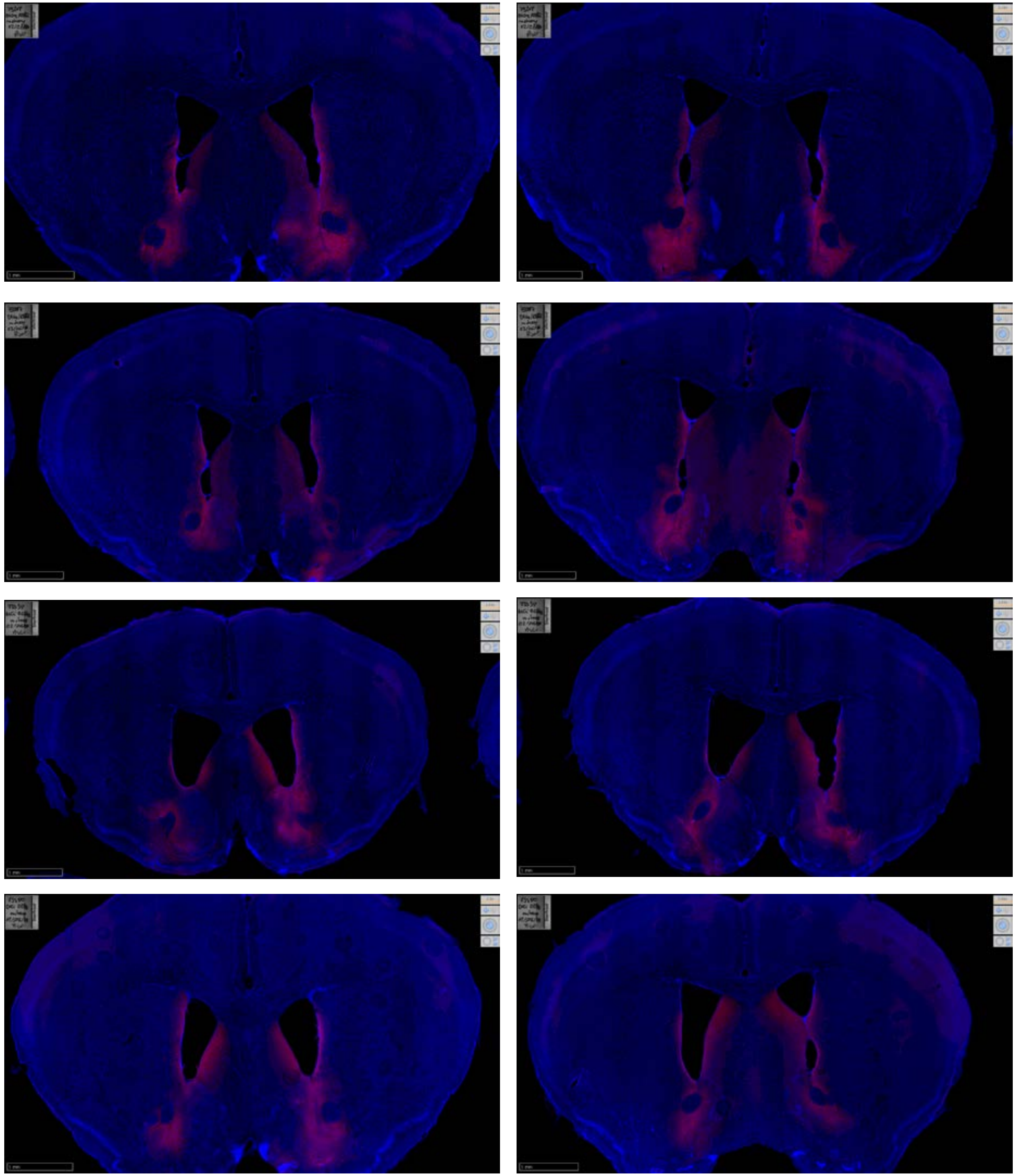

83545
D1Gi
D2Gq

83513
D1Gi
D2Gq

83582
D1Gi
D2Gq

83606
D1Gq
D2Gi

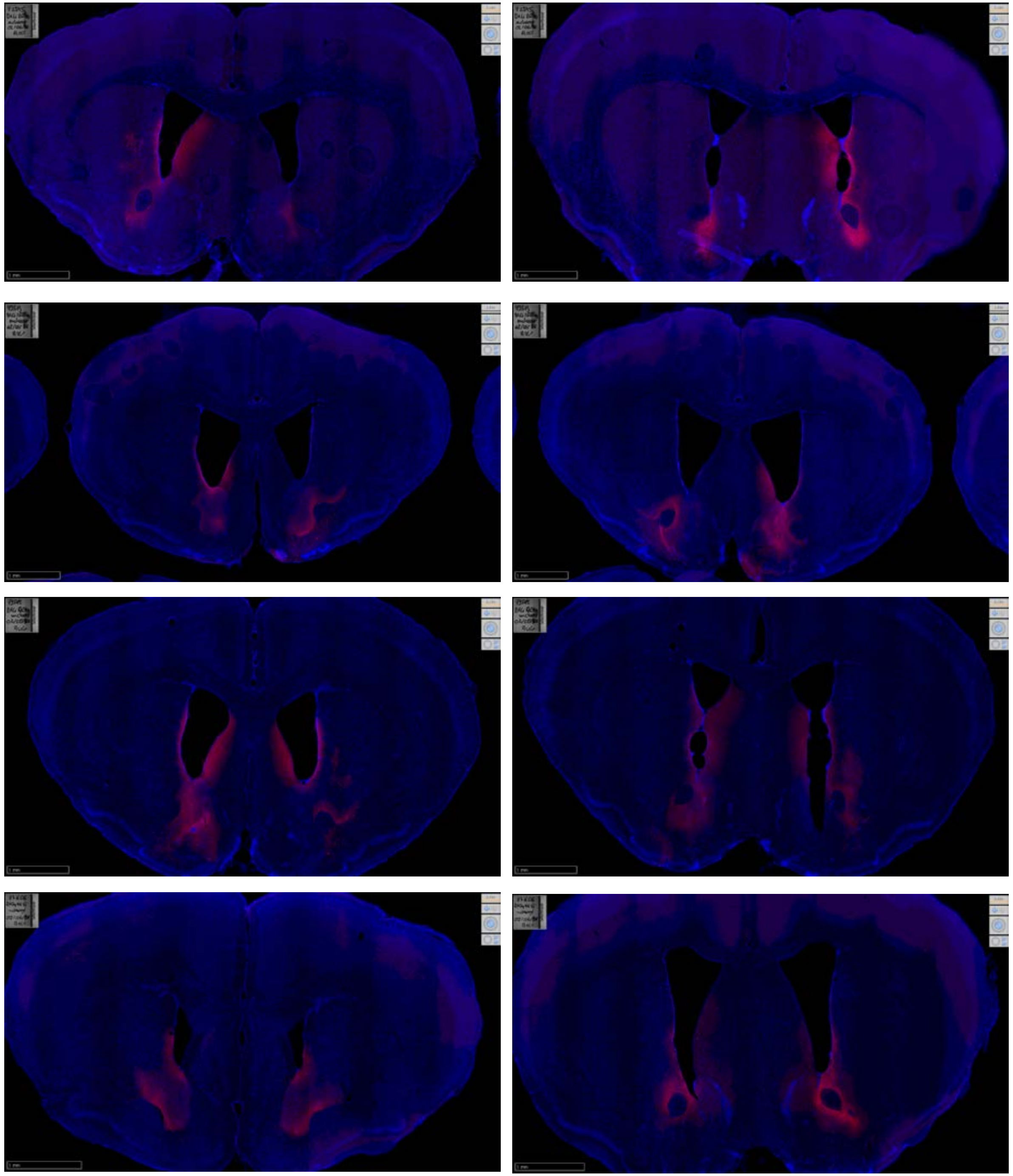

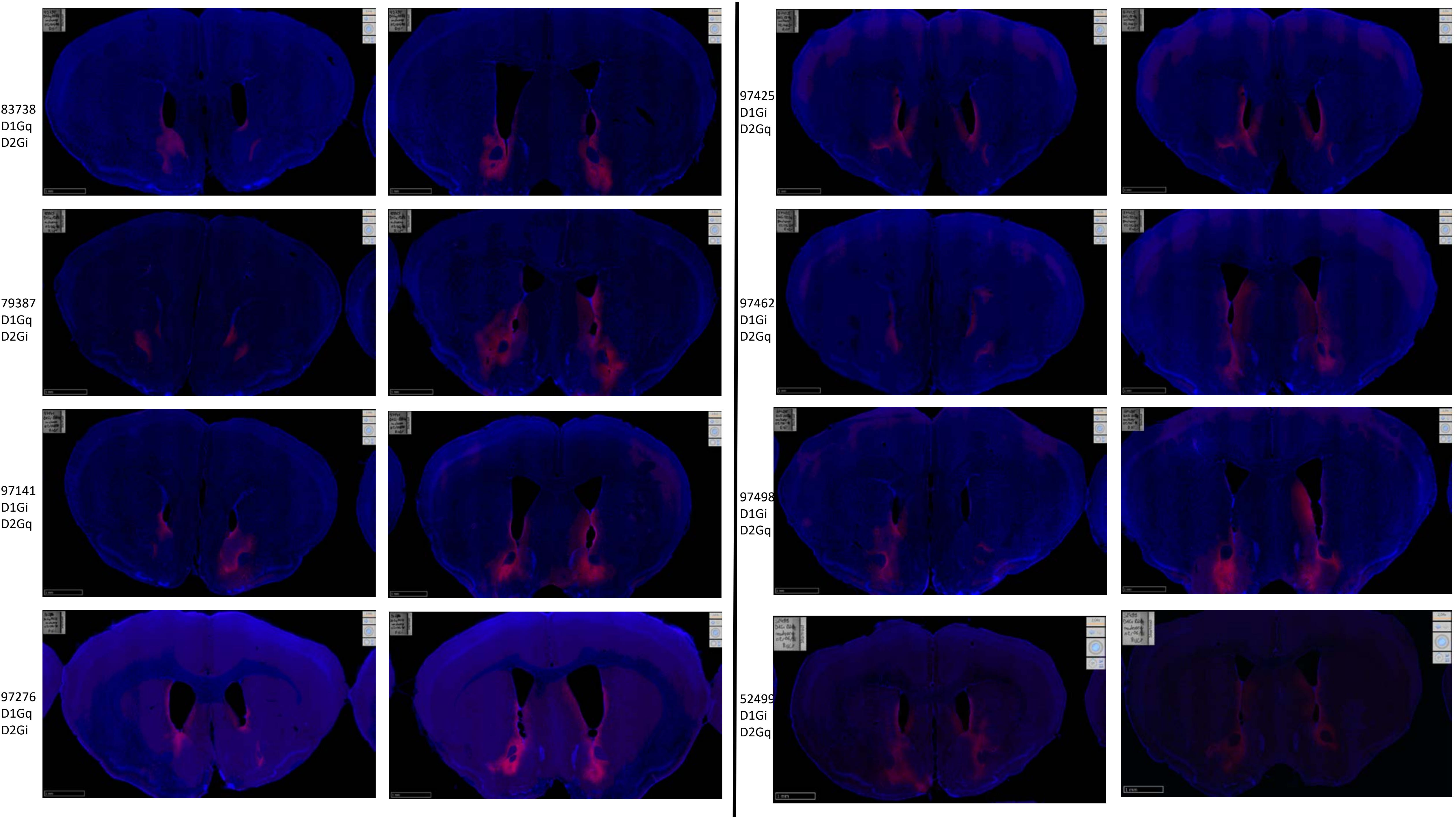
83738
D1Gq
D2Gi
79387
D1Gq
D2Gi
97141
D1Gi
D2Gq
97276
D1Gq
D2Gi
97425
D1Gi
D2Gq
97462
D1Gi
D2Gq
97498
D1Gi
D2Gq
52499
D1Gi
D2Gq

D1flpD1cre mice (Fig 6; D1GiD2Gq = 9, D1GiD2Gq = 8)

3658
D1Gi
D2Gq

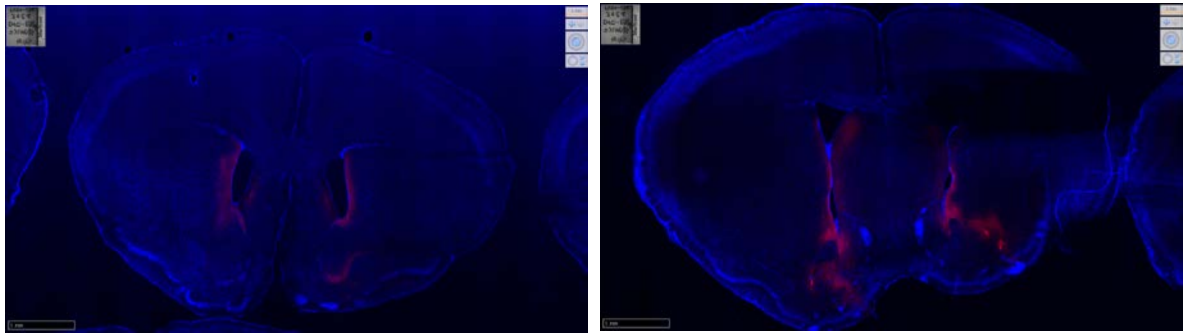

45272
D1Gi
D2Gq

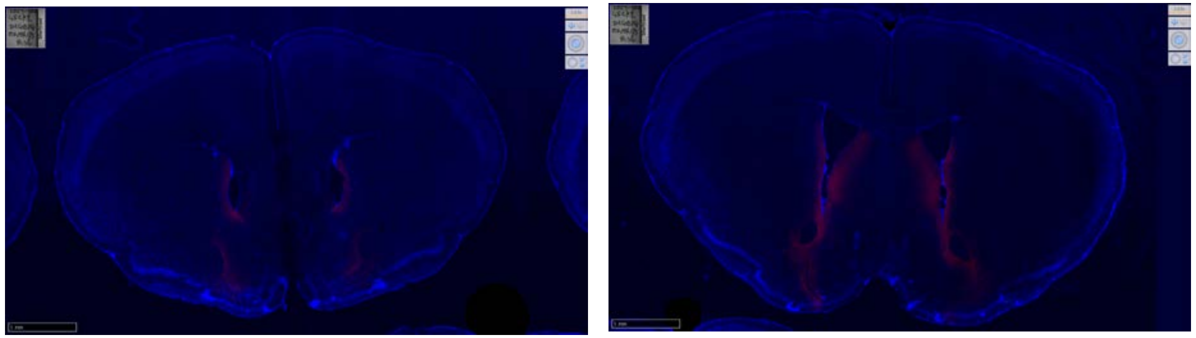

53742
D1Gq
D2Gi

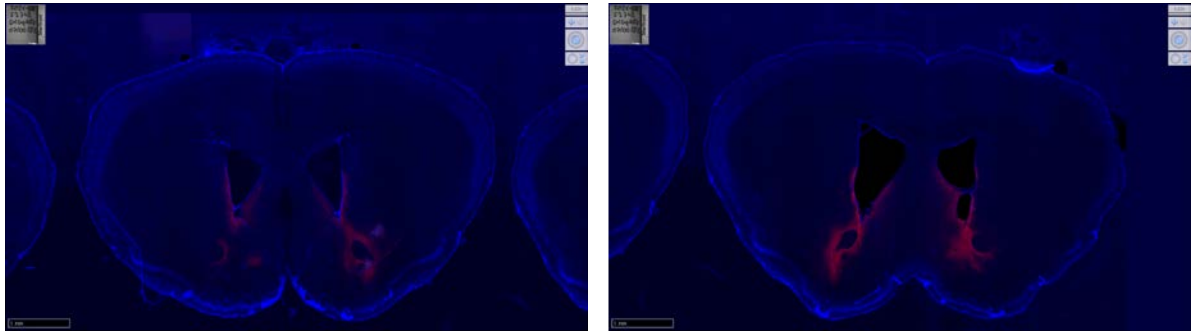

67143
D1Gi
D2Gq

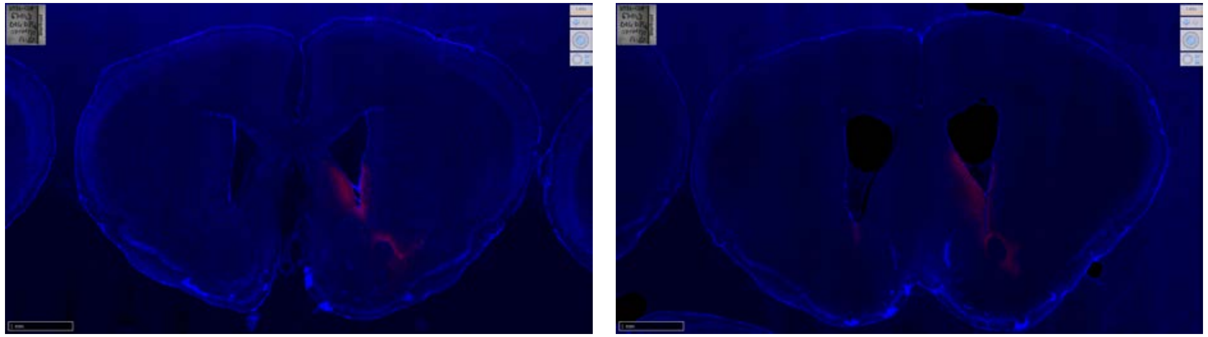

67470
D1Gi
D2Gq

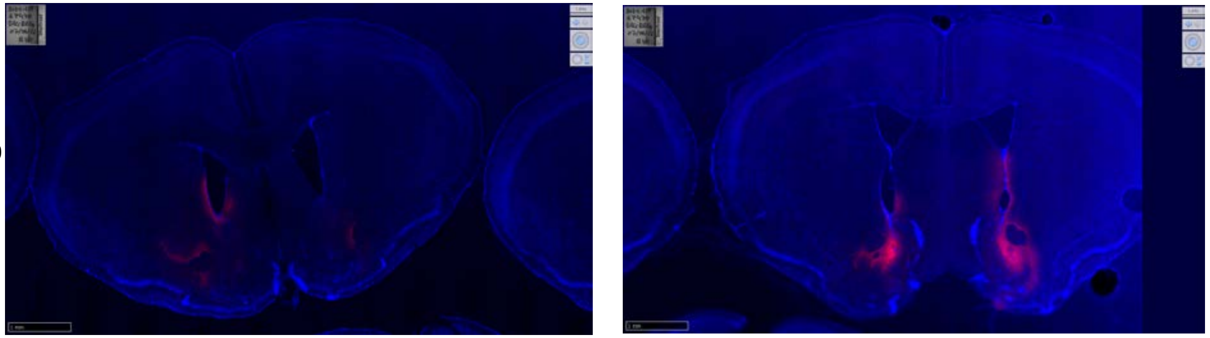

67476
D1Gq
D2Gi

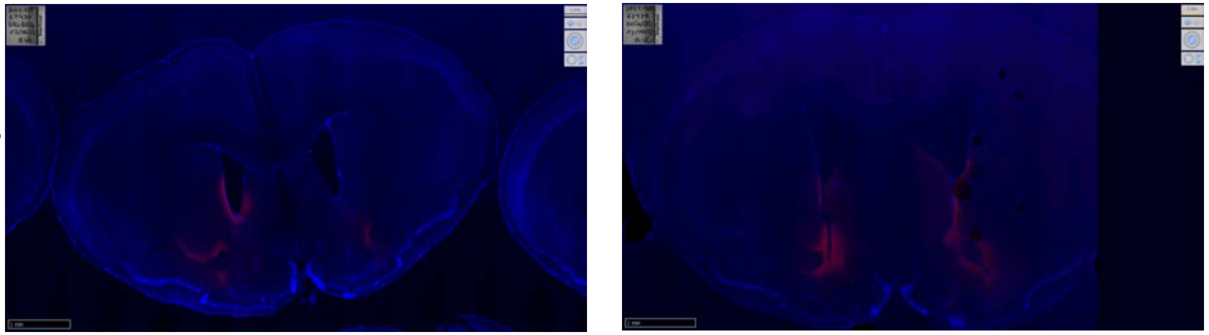

67536
D1Gq
D2Gi

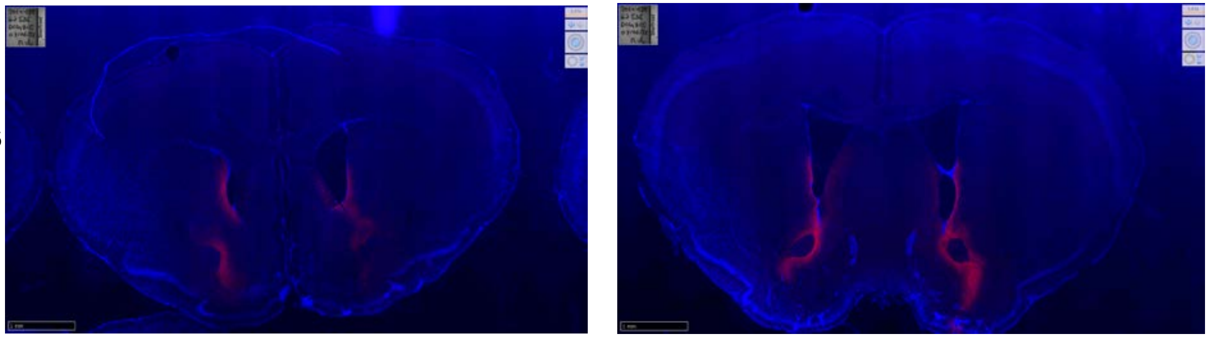

67967
D1Gq
D2Gi

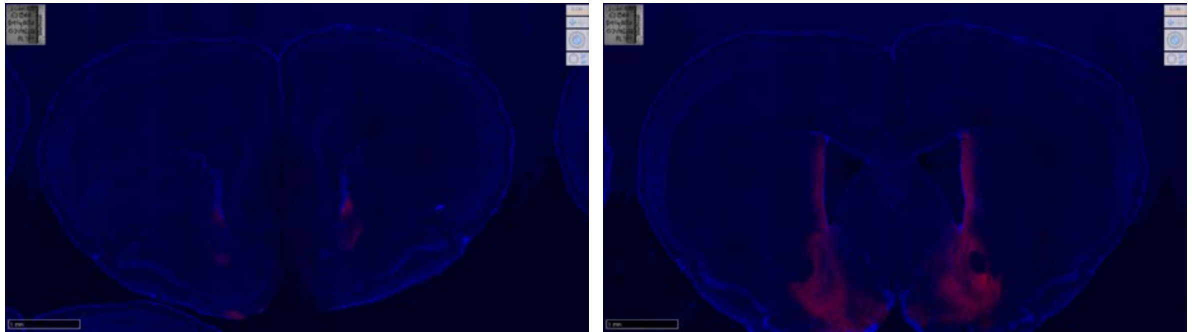

77256
D1Gi
D2Gq

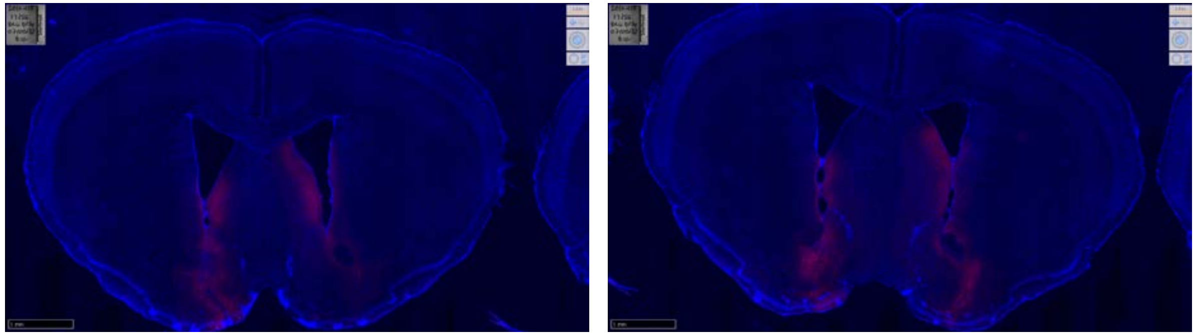

68971
D1Gq
D2Gi

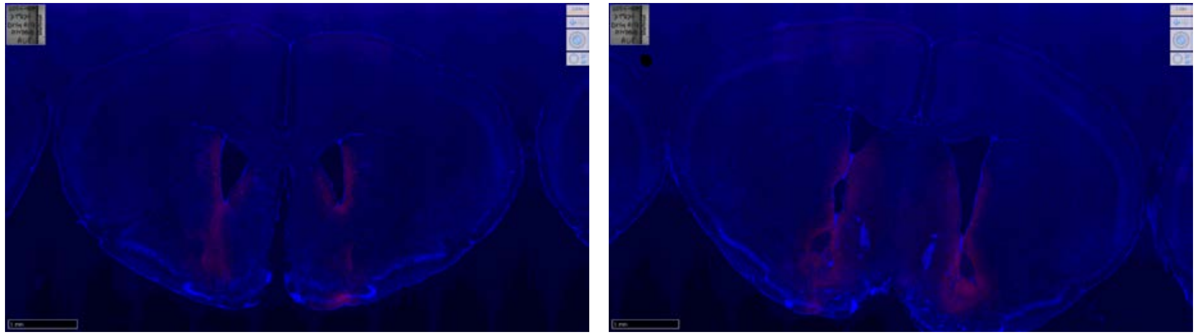

67110
D1Gi
D2Gq

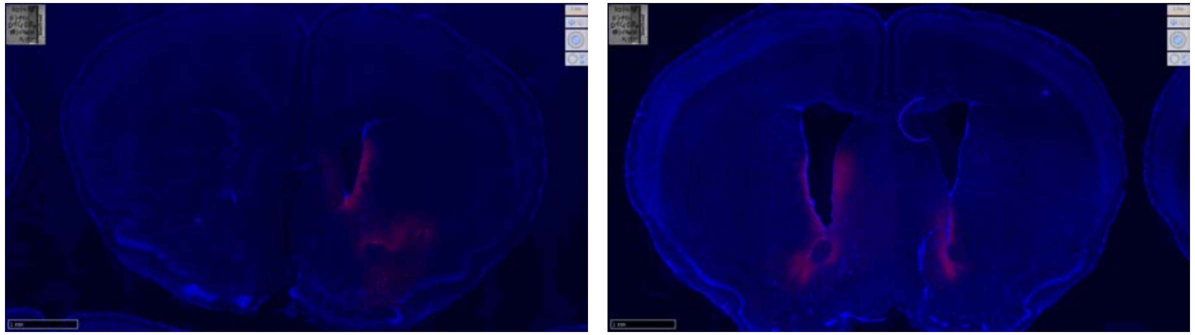

79373
D1Gi
D2Gq

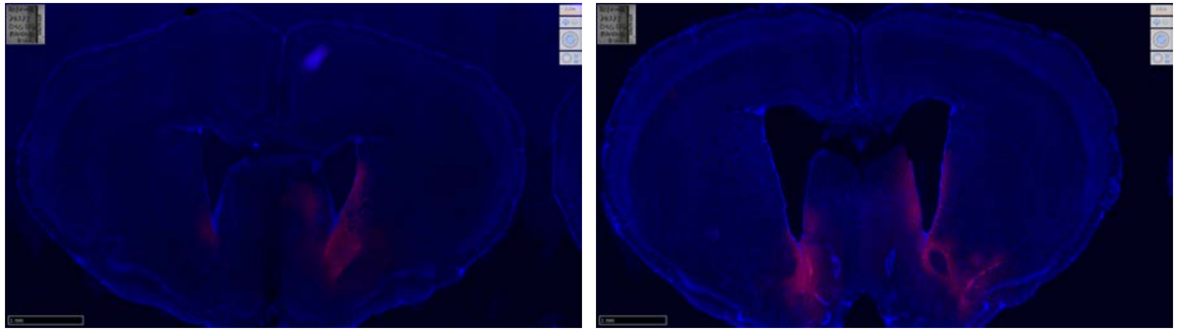

79375
D1Gq
D2Gi

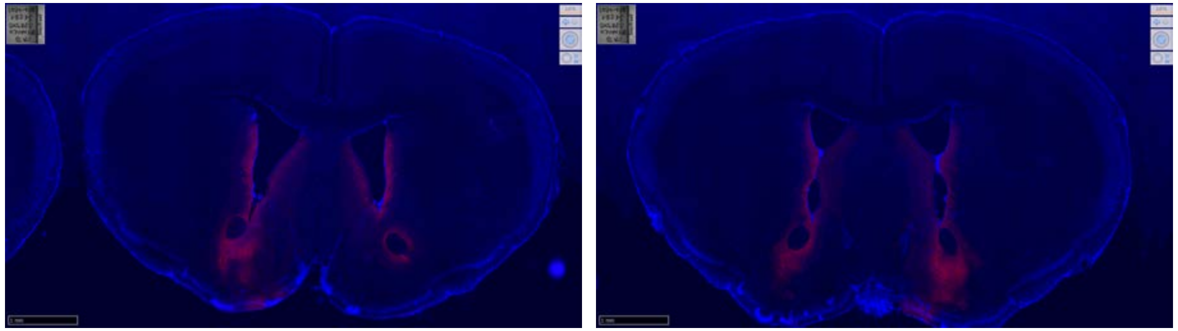

83470
D1Gi
D2Gq

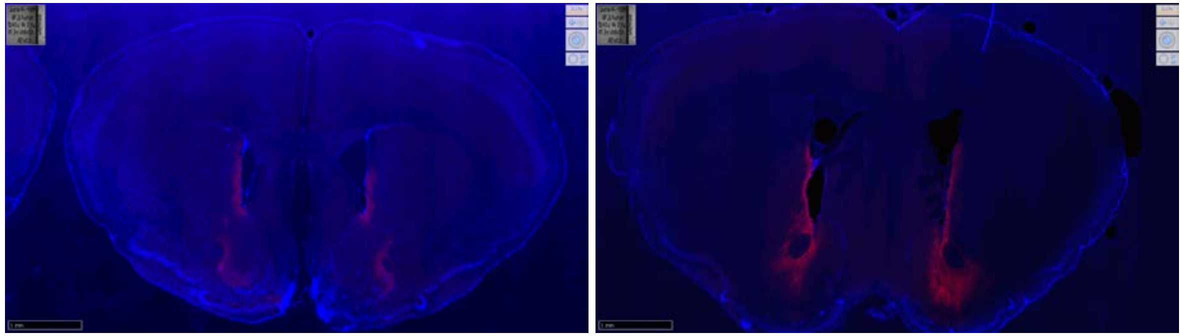

83530
D1Gq
D2Gi

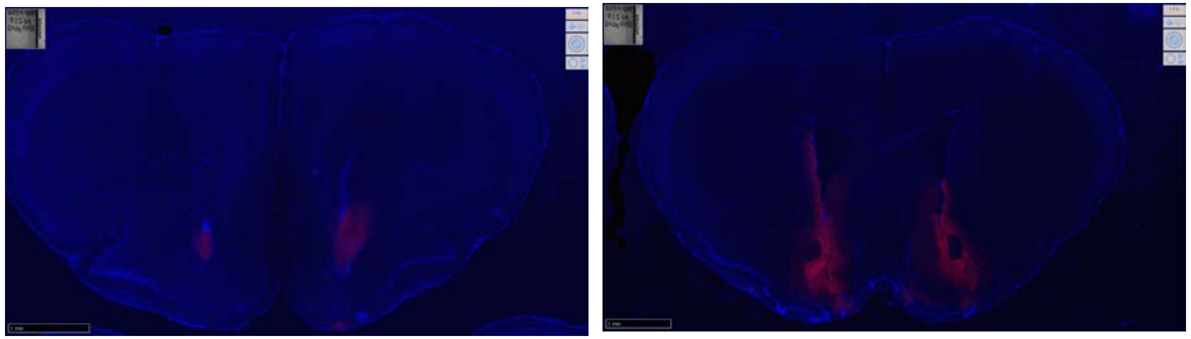

83755
D1Gq
D2Gi

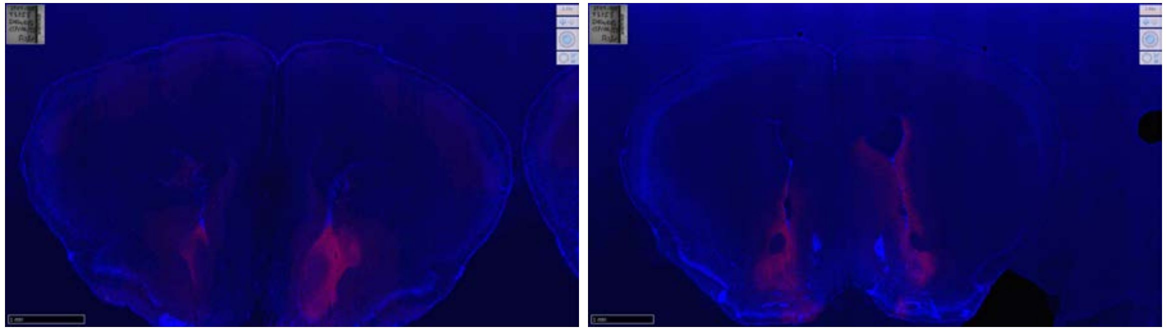

97276
D1Gi
D2Gq

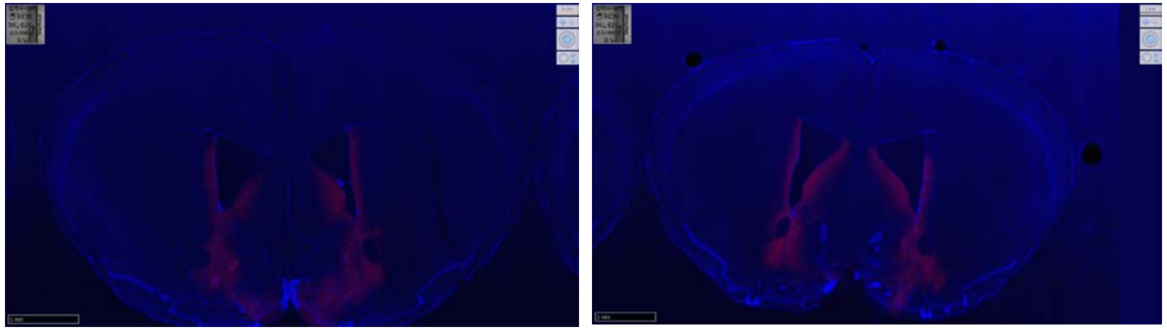

Supplement: Supplementary file 1 — Supplementary Information [file 41467_2024_46874_MOESM1_ESM.pdf]
