# Supplementary material for: Nucleus accumbens D1- and D2-expressing neurons control the balance between feeding and activity-mediated energy expenditure
Source: Nat Commun. 2024 Mar 21;15:2543. doi: 10.1038/s41467-024-46874-9 (PMC10958053; doi:10.1038/s41467-024-46874-9)
Supplement: Supplementary file 2 — Reporting Summary [file 41467_2024_46874_MOESM2_ESM.pdf]

nature portfolio

Corresponding author(s): Pierre Trifilieff; Roman Walle

Last updated by author(s): Feb 22, 2023

Reporting Summary

Nature Portfolio wishes to improve the reproducibility of the work that we publish. This form provides structure for consistency and transparency in reporting. For further information on Nature Portfolio policies, see our Editorial Policies and the Editorial Policy Checklist.

Statistics

For all statistical analyses, confirm that the following items are present in the figure legend, table legend, main text, or Methods section.

n/a	Confirmed	
☐	☒	The exact sample size (*n*) for each experimental group/condition, given as a discrete number and unit of measurement
☐	☒	A statement on whether measurements were taken from distinct samples or whether the same sample was measured repeatedly
☐	☒	The statistical test(s) used AND whether they are one- or two-sided<br>*Only common tests should be described solely by name; describe more complex techniques in the Methods section.*
☐	☒	A description of all covariates tested
☐	☒	A description of any assumptions or corrections, such as tests of normality and adjustment for multiple comparisons
☐	☒	A full description of the statistical parameters including central tendency (e.g. means) or other basic estimates (e.g. regression coefficient) AND variation (e.g. standard deviation) or associated estimates of uncertainty (e.g. confidence intervals)
☐	☒	For null hypothesis testing, the test statistic (e.g. *F*, *t*, *r*) with confidence intervals, effect sizes, degrees of freedom and *P* value noted<br>*Give P values as exact values whenever suitable.*
☒	☐	For Bayesian analysis, information on the choice of priors and Markov chain Monte Carlo settings
☒	☐	For hierarchical and complex designs, identification of the appropriate level for tests and full reporting of outcomes
☒	☐	Estimates of effect sizes (e.g. Cohen's *d*, Pearson's *r*), indicating how they were calculated

Our web collection on statistics for biologists contains articles on many of the points above.

Software and code

Policy information about availability of computer code

Data collection	Operant conditioning data were collected with the Poly software (Imétronic), fiber photometry data were acquired with Doric Neuroscience Studio software, fiber photometry signals were analyzed using MATLAB R2021b (MathWorks), electrophysiological data were collected with SPIKE 2 software.
Data analysis	Custom codes used for this study are available here: https://github.com/Trifilieff-Lab/Walle_2024_NatCommun with DOI: https://doi.org/10.5281/zenodo.10669866

For manuscripts utilizing custom algorithms or software that are central to the research but not yet described in published literature, software must be made available to editors and reviewers. We strongly encourage code deposition in a community repository (e.g. GitHub). See the Nature Portfolio guidelines for submitting code & software for further information.

Data

Policy information about availability of data

All manuscripts must include a data availability statement. This statement should provide the following information, where applicable:

- Accession codes, unique identifiers, or web links for publicly available datasets
- A description of any restrictions on data availability
- For clinical datasets or third party data, please ensure that the statement adheres to our policy

The data that support the findings of this study are available within this article and its Supplementary Materials and are available from the corresponding authors upon request. Source data are provided with this paper.

Research involving human participants, their data, or biological material

Policy information about studies with human participants or human data. See also policy information about sex, gender (identity/presentation), and sexual orientation and race, ethnicity and racism.

Reporting on sex and gender	N/A
Reporting on race, ethnicity, or other socially relevant groupings	N/A
Population characteristics	N/A
Recruitment	N/A
Ethics oversight	N/A

Note that full information on the approval of the study protocol must also be provided in the manuscript.

Field-specific reporting

Please select the one below that is the best fit for your research. If you are not sure, read the appropriate sections before making your selection.

☒ Life sciences ☐ Behavioural & social sciences ☐ Ecological, evolutionary & environmental sciences

For a reference copy of the document with all sections, see nature.com/documents/nr-reporting-summary-flat.pdf

Life sciences study design

All studies must disclose on these points even when the disclosure is negative.

Sample size	No statistical measure was used to determine sample size. Sample size for behavioral experiments was chosen based on comparable experiments previously performed by our lab (Ducrocq et al., 2020 Cell Metab.; Ducrocq et al., 2019, Obesity). For fiber photometry experiments, size was consistent with reports with similar experiments (Patriarchi et al., 2018, Science; Salinas et al., 2023, Nat. Comm.)
Data exclusions	Some animals were excluded based on statistical analysis for outliers (Grubb's test), or due to off-target expression of viral vectors.
Replication	For behavioral experiments, free-running experiments, and free-feeding experiments with chemogenetic manipulations of dopaminoceptive neurons, number of mice used for each treatments and number of trials are indicated in the method section and table 1 of supplementary information. Operant conditioning experiments using CNO as a DREADD ligand were replicated twice in independent cohorts. The effect of chronic concomittant manipulation of both MSN subpopulations on food intake and wheel running, in ad libitum conditions and under food deprivation was confirmed in a second cohort using regular chow diet. For fiber photometry recording in pavlovian conditioning using the dLight sensor, experiments were reproduced in 4 D1-cre mice (CS: 252 trials under saline, 168 trials under CNO; lick: 271 trials under saline, 186 trials under CNO) and 3 D2-cre mice (CS: 170 trials under saline, 105 trials under CNO; lick: 192 trials under saline, 112 trials under CNO).For fiber photometry recording in pavlovian conditioning using the GCaMP sensor, experiments were reproduced in 5 D1-cre mice (CS: 528 trials ; lick: 593 trials) and 5 D2-cre mice (CS: 591 trials; lick: 660 trials). For fiber photometry recording during feeding and running using the GCaMP sensor, experiments were reproduced in 5 D1-cre and 5 D2-cre mice (12 sessions per animal; 59 trials for D1-cre mice, 60 trials for D2-cre mice).
Randomization	animals were randomly assigned to the various groups.
Blinding	For most behavioral procedures, two experimenters were involved, one in charge of injections (CNO/saline), the other performing the behavioral tasks. The latter was blind of animals’ genotype, viral vector and injection. Primary analyses (behaviors identification and extraction of fiber photometry signal) were performed blind to the experimental conditions.

Reporting for specific materials, systems and methods

We require information from authors about some types of materials, experimental systems and methods used in many studies. Here, indicate whether each material, system or method listed is relevant to your study. If you are not sure if a list item applies to your research, read the appropriate section before selecting a response.

Materials & experimental systems

n/a	Involved in the study
☐	☒ Antibodies
☒	☐ Eukaryotic cell lines
☒	☐ Palaeontology and archaeology
☐	☒ Animals and other organisms
☒	☐ Clinical data
☒	☐ Dual use research of concern
☒	☐ Plants

Methods

n/a	Involved in the study
☒	☐ ChIP-seq
☒	☐ Flow cytometry
☒	☐ MRI-based neuroimaging

Antibodies

Antibodies used

all the antibodies used in this study are listed in table 1 with provider and identifier.
Primary antibody anti-DS red (rabbit) Takara Bio 632496
Primary antibody anti-GFP (mouse) Clontech 632280
Primary antibody anti-Choline acetyl transferase (goat) Merck Millipore AB144P
Secondary antibody anti-rabbit alexa 568 (donkey) ThermoFisher A-10042
Secondary antibody anti-rabbit alexa 488 (donkey) ThermoFisher A-21206
Secondary antibody anti-goat alexa 568 (donkey) ThermoFisher A-11057
Secondary antibody anti-goat alexa 488 (donkey) ThermoFisher A-11055

Validation

anti-DS red: https://www.takarabio.com/products/antibodies-and-elisa/fluorescent-protein-antibodies/red-fluorescent-protein-antibodies
Anti-GFP: not available
Anti-ChAT : https://www.merckmillipore.com/FR/fr/product/Anti-Choline-Acetyltransferase-Antibody,MM_NF-AB144P?ReferrerURL=https%3A%2F%2Fwww.google.com%2F

Animals and other research organisms

Policy information about studies involving animals; ARRIVE guidelines recommended for reporting animal research, and Sex and Gender in Research

Laboratory animals

12-28 week old male C57Bl6J mice were used.
The following strains were used:
- Tg(Drd1-cre)FK150Gsat/Mmucd GENSAT RRID: MMRRC_029178-UCD
- Tg(Drd2-cre)ER44Gsat/Mmucd GENSAT RRID: MMRRC_017263-UCD
- D1Flp (D1 expressing flippase and YFP) Produced by the Laboratory of B. Giros

Wild animals

The study did not involve wild animals

Reporting on sex

Study was only performed in male mice

Field-collected samples

The study did not involve samples collected from the field.

Ethics oversight

All animal care and experimental procedures were in accordance with the INRAE Quality Reference System and to French legislations (Directive87/148, Ministère de l'Agriculture et de la Pêche) and European (Directive 86/609/EEC). They followed ethical protocols approved by the Region Aquitaine Veterinary Services (Direction Départementale de la Protection des Animaux, approval ID: B33-063-920) and by the animal ethic committee of Bordeaux CEEA50. Every effort was made to minimize suffering and reduce the number of animals used.

Note that full information on the approval of the study protocol must also be provided in the manuscript.

Plants

Seed stocks	N/A
Novel plant genotypes	N/A
Authentication	N/A
